# Genetic landscape of T cells identifies synthetic lethality for T-ALL

**DOI:** 10.1038/s42003-021-02694-x

**Published:** 2021-10-20

**Authors:** Connor P. O’Meara, Lucia Guerri, Divine-Fondzenyuy Lawir, Fernando Mateos, Mary Iconomou, Norimasa Iwanami, Cristian Soza-Ried, Katarzyna Sikora, Iliana Siamishi, Orlando Giorgetti, Sarah Peter, Michael Schorpp, Thomas Boehm

**Affiliations:** 1 Department of Developmental Immunology, Max Planck Institute of Immunobiology and Epigenetics, 79108 Freiburg, Germany; 2 Bioinformatics Unit, Max Planck Institute of Immunobiology and Epigenetics, 79108 Freiburg, Germany; 3 Faculty of Medicine, University of Freiburg, Freiburg, Germany; 4 Present Address: Laboratory of Neurogenetics, National Institute of Alcohol Abuse and Alcoholism (NIAAA), National Institutes of Health (NIH), Bethesda, MD USA; 5 Present Address: Institute of Zoology, Developmental Biology Unit, University of Cologne, Cologne, Germany; 6 Present Address: Center for Bioscience Research and Education, Utsunomiya University, Utsunomiya, Japan; 7 Present Address: Fundacion Oncoloop & Center for Nuclear Medicine, Santiago, Chile; 8 Present Address: Luxembourg Centre for Systems Biomedicine, University of Luxembourg, Esch-sur-Alzette, Luxembourg

**Keywords:** Genetics, Immunology

## Abstract

To capture the global gene network regulating the differentiation of immature T cells in an unbiased manner, large-scale forward genetic screens in zebrafish were conducted and combined with genetic interaction analysis. After ENU mutagenesis, genetic lesions associated with failure of T cell development were identified by meiotic recombination mapping, positional cloning, and whole genome sequencing. Recessive genetic variants in 33 genes were identified and confirmed as causative by additional experiments. The mutations affected T cell development but did not perturb the development of an unrelated cell type, growth hormone-expressing somatotrophs, providing an important measure of cell-type specificity of the genetic variants. The structure of the genetic network encompassing the identified components was established by a subsequent genetic interaction analysis, which identified many instances of positive (alleviating) and negative (synthetic) genetic interactions. Several examples of synthetic lethality were subsequently phenocopied using combinations of small molecule inhibitors. These drugs not only interfered with normal T cell development, but also elicited remission in a model of T cell acute lymphoblastic leukaemia. Our findings illustrate how genetic interaction data obtained in the context of entire organisms can be exploited for targeted interference with specific cell types and their malignant derivatives.

## Abstract (web summary)

O’Meara et al. utilize a panel of zebrafish mutants to perform a whole organism genetic interaction screen, examining the network regulating T cell differentiation. The authors use a T cell acute lymphoblastic leukemia (T-ALL) model to integrate the effects of small molecule inhibitors of the T cell differentiation pathway and establish a combination therapy for T-ALL in juvenile zebrafish.

## Introduction

Exhaustive pairwise combinatorial screens of genetic variants in unicellular organisms, such as *E. coli*^1^, *S. cerevisiae*^2^, and *S. pombe*^3^, and in cell lines of multicellular organisms, such as *D. melanogaster*^4^, and *H. sapiens*^5–7^ have illuminated the fundamental structure of genetic networks regulating cell fitness. These studies have also unravelled the molecular basis of general molecular rules underlying genetic interaction and resolved previously undefined gene functions in complex regulatory networks. Additional multiparametric phenotyping studies^4,8^, albeit focusing on more manageable collections of genes, have added additional complexity, revealing sub-networks regulating phenotype-specific interactions (e.g., cell growth versus cell division). However, by design, approaches using cell lines fall short of capturing the physiology of an entire organism. In contrast, a genetic interaction screen focusing on one or few cell types in the context of a living vertebrate organism has the potential to reveal cell non-autonomous and tissue-specific components of genetic networks, an approach that has yet to be fully explored^9^. Here, we describe our efforts to establish the genetic network governing the differentiation of T cells using the zebrafish as a vertebrate model organism.

Genetic interaction is said to occur when the phenotype of a double-mutant organism deviates from the expected neutral phenotype. It can be positive (alleviating), when the phenotype is less severe than expected, or negative (synthetic) when the combination of two individually benign gene mutations into a single genetic background results in a more severe phenotype, such as loss of cell viability^10^. The latter outcome is particularly attractive from the viewpoint of cancer therapy. In this context, synthetic lethality screens seek to identify and perturb genes that are required for the survival of a target cancer cell carrying a specific oncogenic mutation, as exemplified by the success of small molecule PARP inhibitors in patients with *BRCA1*-deficient tumours^11^. Nonetheless, the known tissue-dependence of genetic interactions^12^ and unexpected collateral damage outside the target tissue suggest that such synthetic lethality screens are ideally conducted in the context of a whole organism, thereby also incorporating potentially important non-cell-autonomous modulatory effects mediated by the tumour microenvironment^12^.

T-cell acute lymphoblastic leukaemia (T-ALL) arises from an early T cell progenitor in the thymus that somatically acquired a stemness phenotype as a result of ectopic gene activation following chromosomal translocations often involving T cell receptor genes^13^, and/or genetic and epigenetic defects in key lineage determinants, such as *NOTCH1*^14^. Interestingly, inhibiting the activities of genes highly expressed during early T cell differentiation, such as *BCL-2* or *JAK1*/*JAK2*, significantly reduced leukaemic burden in mouse T-ALL xenografts, regardless of their mutation status^15–17^, suggesting that interference with the function of genes expressed in a tissue-specific or tissue-restricted fashion offers an opportunity for targeted tumour therapy.

Here, we delineated the genetic network underlying the development of T cells in zebrafish, established the nature of synthetic lethal interactions, and exploited this information for combinatory inhibitor treatments that proved effective in preventing tumour progression in an in vivo model of T-ALL.

**Fig. 1:**
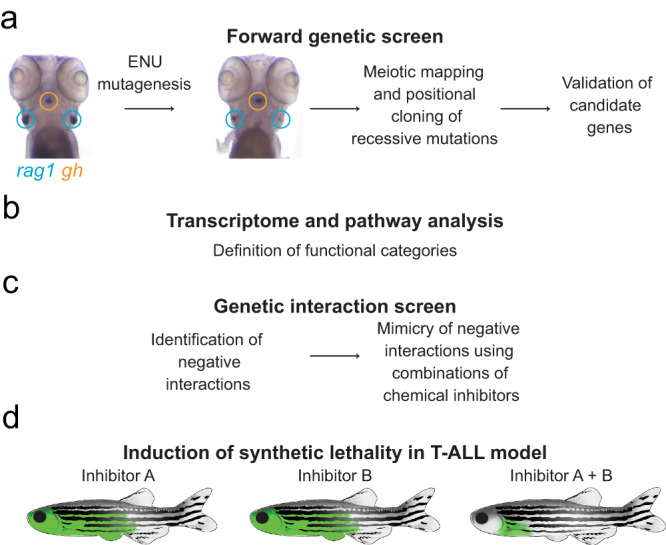
Outline of the four major components of the current study. **a** Identification of genetic variants perturbing T cell development through large-scale forward genetic screens in zebrafish larvae. **b** Delineation of the genetic network underlying the development of T cells by pairwise interaction analyses. **c** Phenocopy of a subset of genetic interactions using small molecule inhibitors. **d** Tumour treatment in an in vivo model of T-ALL based on the information obtained on synthetic lethal interactions.

The present study encompassed four steps (Fig. 1). In step 1, genetic variants perturbing T cell development were identified through large-scale forward genetic screens in zebrafish larvae (Fig. 1a); in step 2, the genetic network underlying the development of T cells was delineated by pairwise interaction analyses (Fig. 1b); in step 3, some of the genetic interactions were phenocopied using small molecule inhibitors (Fig. 1c); in step 4, the information obtained on synthetic lethal interactions was exploited for tumour treatment in an in vivo model of T-ALL (Fig. 1d).

Our results illustrate how the structure of a genetic network, established in the context of an entire organism rather than in a particular cell line, guides the selection of drug combinations that selectively interfere with the function and/or viability of a specific cell type in vivo.

## Results

### T lymphocyte-focused forward genetic screens

To establish the key nodes in the genetic network regulating the differentiation of immature T cells, we conducted two forward ENU mutagenesis screens^18,19^ to identify recessive genes regulating developing T cells in the thymus of zebrafish in an unbiased manner. Using whole-mount RNA in situ hybridization at 5 days post fertilization (d.p.f.) as a read-out, we identified mutations that impaired larval T cell development (defined as absence or reduction in recombination activating gene [*rag1*] expression), but spared the hypophysis (defined as unchanged growth hormone gene [*gh*] expression) and lacked craniofacial defects as assessed by microscopic inspection. Thus, by way of design, the initial selection focused on the identification of genetic variants without overt pleiotropic effects. In order to quantify the extent of thymopoiesis, the ratio of *rag1/gh* hybridization signals was calculated from their two-dimensional projections after whole mount RNA in situ hybridization^18–20^, and the distribution of values of *rag1/gh* ratios in mutant crosses was compared to that of control embryos. In the gynogenetic screen (for details, see ref. ^18^), 281 mutagenized genomes were analysed; in 25 instances, clutches were observed, in which about 50% of the embryos exhibited abnormal *rag1* signals with normal *gh* gene expression pattern (that is, a statistically significant reduction of *rag1/gh* ratios when compared to wild-type embryos) and normal craniofacial structures. Ultimately, three mutant lines could be established, corresponding to ~1% of the number of genomes screened. In a subsequent F3 screen (for details, see ref. ^19^), F_3_ clutches of 4584 F_2_ families, representing 4253 mutagenized haploid genomes, were screened. A total of 141 mutants with reduced *rag1* signals but without severe craniofacial defects and normal *gh* expression patterns were detected in the primary analysis. Ultimately, 42 mutant lines could be established, again corresponding to ~1% of the number of genomes screened. One allele each of the *top3a* gene was identified in both the F3 and gynogenetic screens^21^; two alleles of *ikzf1* were identified in the F3 screen^19,20^ (Table 1). This observation indicates that the two screens combined can be expected to cover a large fraction of genes/pathways regulating T cell development in zebrafish larvae.

**Table 1:** Genetic variants identified in ENU forward genetic screen.

Mutant	Allele^a^	Affected gene	ENSEMBL Gene ID^b^	Molecular defect^c^	Reference
*Haematopoietic development*					
IP109	t25127	*myb*	ENSDARG00000053666	p.I181N	^68^
II032	t25880	*ikzf1*^**d**^	ENSDARG00000013539	p.R489X	^20^
HY022	t21380	*il7r*	ENSDARG00000078970	p.L124FfsX5	^21^
HX157	t22598	*jak1*	ENSDARG00000020625	p.R580X	^21^
IP045	t25078	*jak3*	ENSDARG00000010252	p.Q336X	^21^
JZ061	t26394	*fli1a*	ENSDARG00000054632	p.Q246X	This paper
HK017	t20463	*zbtb17*	ENSDARG00000074548	p.Q562K	^89^
*DNA replication/repair*					
HG010	t20320	*pole1*	ENSDARG00000058533	p.I633K	^20^
IG335	t23336	*mcm10*	ENSDARG00000045815	p.L248R	^20^
HU319	t24593	*atad5a*	ENSDARG00000070568	p.L430X	This paper
WW20/12	fr17	*top3a*^**e**^	ENSDARG00000052827	p.I531S	^90^
IY071	t25501	*dnmt1*	ENSDARG00000030756	p.N1391K	^20^
*Cell cycle regulation*					
JM087	t26113	*anapc1*	ENSDARG00000075687	p.Y86X	This paper
IT429	t25333	*nek7*	ENSDARG00000056966	p.Q117X	This paper
*mRNA processing*					
KW059	t26426	*snapc3*	ENSDARG00000101474	p.C297X	^20^
WW18/10	fr100	*lsm8*	ENSDARG00000091656	p.E72X	^20^
KL069	t26393	*gemin5*	ENSDARG00000079257	p.Y437X	^20^
IU191	t25877	*cstf3*	ENSDARG00000018904	p.D313VfsX7	^20^
HJ028	t20450	*upf1*	ENSDARG00000016302	p.Y163X	^91^
HA343	t22074	*tnpo3*	ENSDARG00000045680	p.R203X	^20^
*Ribosome*					
IG438	t22881	*spata5*	ENSDARG00000104869	p.R679X	This paper
HP327	t24596	*nol9*	ENSDARG00000077751	p.Q162X	This paper
JI065	t26214	*pnrc1*	ENSDARG00000043904	p.R91H	This paper
JM052	t26337	*fcf1*	ENSDARG00000102333	p.R44G	This paper
*Chaperone & protein transport/stability*					
HI020	t22231	*tbcb*	ENSDARG00000068404	p.Y182X	This paper
IL015	t23758	*unc45a*	ENSDARG00000103643	IVS1-1G>A (1-1G>A)	This paper
IM087	t24920	*ube3d*	ENSDARG00000026178	p.L352P	This paper
*Phosphoinositol metabolism*					
HG002	t20082	*pi4kaa*	ENSDARG00000076724	p.Y800X	This paper
IG447	t23755	*pip5k1ba*	ENSDARG00000044295	p.T139M	This paper
*Miscellaneous*					
HY062	t24600	*mat2aa*	ENSDARG00000040334	p.Y101X	This paper
JI073	t26215	*naa50*	ENSDARG00000027825	1922-4099del	This paper
KH025	t26216	*eif5*	ENSDARG00000003681	p.Y52X	This paper
JZ007	t25773	*yeats2*	ENSDARG00000078767	IVS25+1G>A (4247+1G>A)	This paper

^a^Isolated in the Freiburg gynogenetic screen (allele designation: frx); all other lines originate from the Tübingen 2000 screen (allele designation: t); see ref. ^19^ for details.

^b^Zv10.

^c^Nomenclature according to ref. ^92^

^d^A second mutant allele of *ikzf1* was identified (p.Q360X [t24980]; ref. ^19^).

^e^A second mutant allele of *top3a* was identified (p.E331X [t22046]; ref. ^90^).

The genomic localizations of zebrafish mutations were determined by meiotic recombination mapping using individual F_3_ fish arising from crosses of F_2_-mutant carriers and the genetically distant WIK wild-type strain^18–20^ and/or whole genome sequencing (see the “Methods” section). After assignment of mutants to particular linkage groups, complementation analysis was carried out for those mutations that mapped to the same chromosomal region to determine whether two mutations causing similar phenotypes reside in the same or in two different genes. To this end, a heterozygous fish carrying one mutation was crossed with a heterozygous fish carrying the other mutation. In general, allelic mutations fail to complement each other in trans-heterozygous embryos, which exhibit the mutant phenotype like homozygotes of either allele. If, however, the mutations are in different genes, the double heterozygous offspring are expected to exhibit a wild-type phenotype, unless epistatic interactions modify the phenotype. The critical intervals identified by high-resolution meiotic recombination mapping often contained less than a dozen candidate genes. Their coding exons (including flanking regions) were then sequenced after PCR amplification from genomic DNA of phenotypically wild-type (that is, a mixture of wild-types and heterozygous fish) and mutant embryos, which were identified by prior RNA in situ hybridization with the *rag1* probe; alternatively, whole genome sequencing was employed to establish the structure of the relevant genomic region. Once the identities of the mutated genes (Table 1) had been established, they were subsequently validated by at least one of several techniques, including CRISPR/Cas9-mediated mutagenesis, knock-down using antisense morpholino oligonucleotides, and mRNA-mediated or BAC DNA-mediated phenotypic rescue (Supplementary Fig. 1 and Supplementary Tables 1–4). In total, 33 candidate genes were successfully validated. Whereas about one third of the identified alleles exhibit deleterious missense mutations, the majority of alleles are predicted to encode truncated proteins (Table 1). Based on their gene ontologies and the information obtained from literature surveys, the candidate genes were tentatively grouped into several functional categories (Table 1), although some of the genes function in more than one pathway. For example, the *dnmt1* gene is listed here in the DNA repair and replication group, which clearly corresponds to its general functional properties; however, the particular mutant allele described here specifically impairs haematopoietic development by primarily affecting the lymphocyte lineage^22,23^. With these caveats in mind, we derived the following categories. Haematopoietic regulators, *c-myb*, *ikzf1, il7r*, *jak1*, *jak3, fli1a, and zbtb17*; DNA repair and replication processes, *pole1*, *mcm10*, *atad5a*, *dnmt1, and top3a*; cell cycle regulation, *anapc1* and *nek7*; mRNA processing, *snapc3*, *lsm8*, *gemin5*, *cstf3*, *upf1, and tnpo3*; ribosome biogenesis, *spata5, nol9, pnrc1*, and *fcf1*; protein folding and stability, *tbcb*, *unc45a*, and *ube3d*; phosphoinositol metabolism, *pi4kaa* and *pip5k1ba*. The four genes belonging to miscellaneous pathways included, *mat2aa* (S-adenosylmethionine synthesis), *naa50* (N-terminal protein acetylation), *eif5* (protein translation), and *yeats2* (histone H3K27ac reader).

### Transcriptional landscapes of mutants

**Fig. 2:**
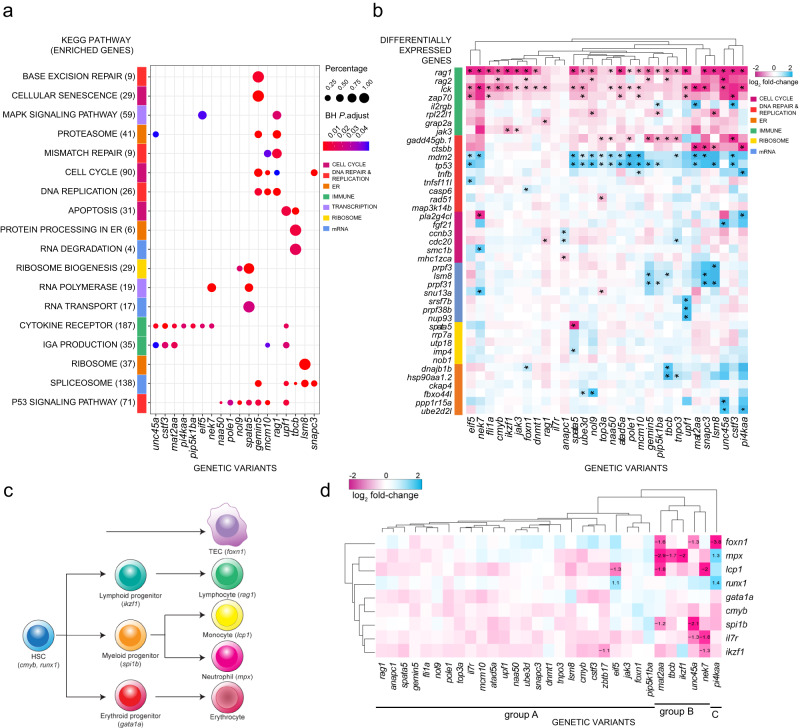
Phenotyping genetic variants identified from ENU screen, grouped by biological function. **a** Transcriptome analysis and ClusterProfiler pathway enrichment of top 1500 differentially expressed genes (DEG) (FDR ≤ 0.05) from each genetic variant. Genetic variants are shown in columns and enriched KEGG pathways in rows, with brackets indicating the number of enriched genes from each pathway; only 18 of 30 genetic variants exhibited significant deregulation of KEGG pathways. Row side colours identify pathways in similar biological functional categories. Point sizes represent the percentages of genes enriched from each pathway for each genetic variant; point colours specify Benjamini–Hochberg (BH)-adjusted *P* values. **b** Top DEG in genetic variants. Genetic variants are shown in columns and DEG in rows, grouped by biological functional categories. The following KEGG identifiers were included. T cell development (T cell receptor—mmu04660, primary immunodeficiency—mmu05340, Notch signaling—mmu04330), DNA synthesis (DNA replication—mmu03030, MMR—dre03430, BER—dre03410, HR—dre03440, p53 signaling—dre04115), cell cycle (apoptosis—dre04210, cell cycle—dre04110, cellular senescence—dre04218), mRNA processing (spliceosome—dre03040, mRNA surveillance—dre03015, nonsense-mediated decay—dre03015), ribosome function (ribosome biogenesis—dre03008, ribosome—dre03010) and endoplasmic reticulum (ER) (protein processing in ER—dre04141, proteasome—dre03015). Asterisks define the top six DEG (|log_2_ fold change| ≥ 0.5) per genetic variant. **c** Schematic of haematopoietic differentiation pathways. Each cell type is marked with a signature gene(s), whose expression levels are taken as indicative of their presence. Note that the thymic epithelium represents a separate lineage and originates from pharyngeal endoderm. At the time of analysis (5 d.p.f.), B cell development has not yet started, so that *rag1* expression levels are indicative of developing T cells. **d** Expression levels of signature genes (excluding *rag1*; see Fig. 2b, and text) as determined by RNA-seq at 5 d.p.f. for all genetic variants. The log_2_ fold changes are indicated. Some of the gene expression changes detected in the RNA-seq analysis were confirmed by RNA in situ hybridization at different stages of development (see Supplementary Fig. 3).

To gain further insight into the functional consequences of the different mutations, we compared the transcriptomes of whole mutant fish larvae and their wild-type siblings at 5 d.p.f. To this end, individual fish were genotyped and wild-type (+/+) and homozygous mutant (m/m) individuals selected for RNA sequencing. We determined the differentially expressed genes in the transcriptomes of 28 mutants from the ENU screen; we also included fish with deleterious mutations in *rag1* (ref. ^24^) and *foxn1* genes. This collection of mutants (Fig. 2a) represents the core functional categories identified in the genetic screens. For each mutant, we subjected their 750 most significantly up- and down-regulated genes, respectively, to KEGG pathway enrichment analysis (Fig. 2a). For 17 ENU mutants and *rag1*-deficient fish, this analysis recovered at least one, but in most cases, several significantly enriched pathways; the most frequently flagged pathways were cytokine receptor (dre04060, dre64274), spliceosome (dre03040), and cell cycle (dre04110) (Fig. 2a). The results of additional analyses confirmed the congruency between KEGG assignment and functional outcome for the mutants grouped into cell cycle, spliceosome, endoplasmic reticulum, and ribosome biogenesis categories (Supplementary Fig. 2a–d). For example, using animals treated with nocodazole as a positive control, and the *fli1a* haematopoietic mutant as a negative control, we found subtle changes of the fractions of cells in G2/M phase in *anapc1* and *nek7* mutants; this was accompanied by evidence for developmental retardation of craniofacial cartilage, a rapidly proliferating tissue that is a sensitive indicator of perturbations of cell proliferation in zebrafish larvae (Supplementary Fig. 2a). Likewise, we confirmed that the patterns of mRNA splicing was perturbed for mutants assigned to the category of mRNA processing mutants (*snapc3*, *lsm8*, *tnpo3*, gemin5) (Supplementary Fig. 2b); a notable exception to this general phenomenon is the *upf1* mutant, affecting the degradation of mRNAs by the non-sense-mediated decay machinery (Table 1). By contrast, mRNA splicing was found to be normal in representatives of other categories (*pole1*, *mcm10*, *zbtb17*, and *tbcb*). Mutants assigned to the category of protein folding and stability were found to cause ER stress, as revealed by upregulation of several indicator proteins (Supplementary Fig. 2c). Finally, mutations in genes associated with ribosome biogenesis (*spata5*, and *nol9*) were found to exhibit significantly altered ratios of 18S and 28S mature ribosomal RNAs, indicative of abnormal processing of rRNA precursors; this phenotypic aspect was normal in other mutants (Supplementary Fig. 2d). Collectively, these data support the assignment of mutants to particular functional categories.

A detailed analysis of the transcriptional landscape of all mutants—focussing on the expression levels of genes representing the KEGG pathways (Fig. 2a) concordant with the known gene ontology of each mutant—revealed uniformly reduced transcripts for *rag1*, albeit to different extents (Fig. 2b). This finding is consistent with the primary selection criterion of mutants, namely reduced signals in the whole mount RNA in situ hybridization. Of note, the expression levels of the growth hormone gene, *gh*, were not changed in any of the mutants, in keeping with  the primary selection criterion for the RNA in situ hybridization screening. These findings indicate that the altered *rag1/gh* ratios observed in the mutants are due to reduced *rag1* expression levels rather than increased *gh* signals (cf., Supplementary Figs. 1 and 3). Expression levels of several T cell-associated genes, such as *rag2*, *lck*, and *zap70*, were also reduced, reinforcing the notion of a severely impaired T cell development in all mutants; in 25 of the 30 mutants analysed, *rag1* belongs to the group of their six most deregulated genes (asteriks in Fig. 2b), with the *lck* gene (encoding a critical regulator of T cell development) being included in this category in 20 mutants. Importantly, similar patterns of transcriptional deregulation (Fig. 2b) were found among mutants within individual gene ontology groups as defined in Table 1; the largely overlapping downstream effects of the individual mutants support their functional association. Collectively, these features define a transcriptional landscape of mutant larval T cell development. For instance, mutations in genes regulating pre-mRNA processing (*lsm8*, *snapc3*, *gemin5*, *tnpo3*, and *upf1*) shared increased expression of spliceosome components (*lsm8*, *prpf3*, and *prpf31*); likewise, *unc45a* and *tbcb* mutations both induced higher levels of genes required in protein homeostasis *(dnajb1b* and *hsp90aa*).

Dysregulation of the p53 signalling pathway occurs in many mutants, except for those of the haematopoietic group (Fig. 2a,b, and Supplementary Fig. 4a). Interestingly, although the developing nervous system in zebrafish embryos is known to be highly susceptible to p53-mediated apoptosis^25^, elevated levels of neuronal apoptosis, defined by double-strand breaks (DSB), were only found in the *mcm10* mutant (Supplementary Fig. 4b); previous findings indicated that inactivation of *mcm10* leads to increased generation of DSB^26^. Hence, although the transcriptome data are indicative of activation of the p53 pathway, this response—with the exception of that in *mcm10* mutants—must be restricted to fewer neuronal and/or non-neuronal cell types in the other mutants. Developing T cells are a prime candidate cell type contributing to the *p53* activation signature in transcriptomes of whole larvae; indeed, in most of the cases examined, their demise can be rescued in the *p53*-deficient background (Supplementary Fig. 4c); failing T cell development cannot be reversed in *dnmt1/p53* and *mat2aa/p53* double mutants and thus most likely is due to aberrations other than p53-mediated apoptosis (Supplementary Fig. 4d).

Next, we examined the impact of the identified mutations on haematopoietic development. To this end, we determined the expression profiles of signature genes that characterize various intermediate steps of haematopoietic differentiation (Fig. 2c). The expression levels of *rag1* were not considered in this analysis, because the detrimental effects of mutations all converge on early T cell differentiation (identified by low expression levels of *rag1* [Fig. 2b]), and hence are not informative in this regard. This analysis partitioned the mutations into three groups (Fig. 2d). The largest group of genes appears to predominantly act in the T cell differentiation pathway with little impact on haematopoietic precursor stages (group A). A smaller group of mutations, such as those affecting *mat2aa*, *tbcb*, *ikzf1*, *unc45a*, and *nek7* impairs the differentiation of both myeloid and lymphoid lineages (group B). The substructures within groups A and B (which is apparent from the clustering shown to the left of the panel) highlight unique aspects of each mutant, and provide the starting point for future in-depth functional analyses. Finally, *foxn1* expression is particularly diminished in *pi4kaa* mutants (C), indicating that the malfunction of the thymic microenvironment underlies failing T cell differentiation in this mutant.

### Tissue-restricted effects of mutants

**Fig. 3:**
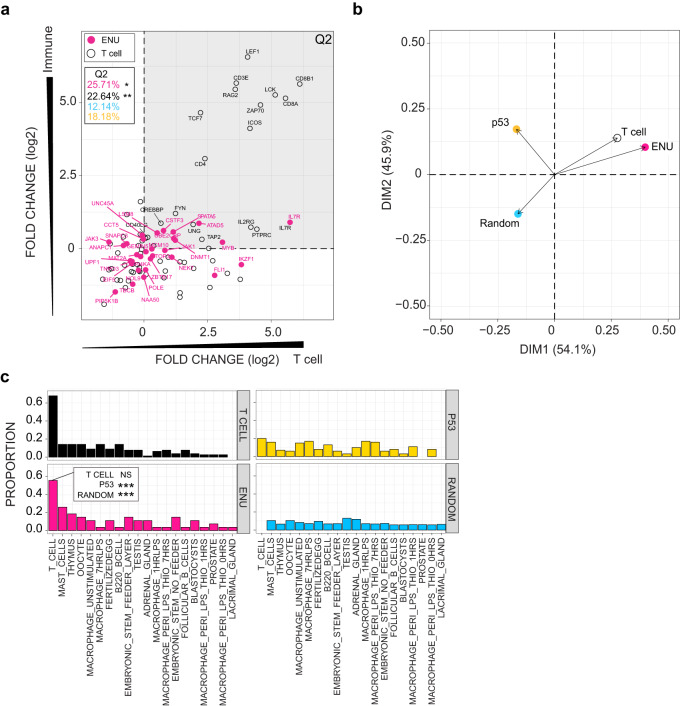
Concordant tissue expression signatures between genes identified in the ENU screens and genes regulating T cell receptor signalling. **a** Expression patterns of mouse homologs of genes identified in the ENU forward genetic screen are compared to genes listed in KEGG pathways designated T cell receptor (mmu04660; T cell), p53 signalling (mmu04115; p53), and a random selection of genes from the mouse genome. BioGPS microarray data were partitioned into four categories (see the “Methods” section); the *y*-axis depicts expression bias according to *immune* and *non-immune* categories, such that genes expressed at higher levels in immune-related cells and tissues have higher values; genes are also partitioned according to T cell and *non-T immune cells* categories on the *x*-axis, such that genes expressed at higher levels in T cells as compared to other immune-related cells receive higher values. Relative expression values (log_2_) for each gene were determined between mean expression values for *immune*/*non-immune* and *T cell/non-T immune cells* partitions. Hence, genes in the upper right quadrant (Q2) represent genes highly expressed by T cells. The percentage of genes in Q2 are depicted; red, genes identified in the forward genetic screen (*ENU* genes); black, *T cell* genes; blue, random gene set (genes not depicted in diagram); yellow; p53 signalling genes (genes not depicted in diagram). *P* values for accumulations was determined by Fisher’s exact test. *P < 0.05; **P < 0.01. **b** Principal component analysis (PCA) on expression data for the four groups of genes analysed in (**a**). **c** Proportion of genes from four groups of genes (T cell-related genes; *ENU* genes; p53 pathway-related genes; and a random selection of genes from the genome) expressed in various tissue and cells of the mouse. Genes were assigned to a specific origin, if their expression levels were significantly greater than background tissue expression (*z* score ≥ 1.96). Proportions of genes highly expressed by each tissue were normalized to the numbers of genes. *P* values for enrichments were determined by Fisher’s exact test. Note that the proportion of *ENU* genes assigned to T cells in the BioGPS list is indistinguishable from that of *T cell* genes. By comparison, the proportion of p53-signalling pathway genes and random gene sets are significantly underrepresented (***P < 0.001).

Our results indicate that T cells are particularly sensitive towards the impaired activities of genes identified in the screen, as haematopoietic cell types other than T cells (haematopoietic progenitors, erythroid and myeloid cells) and the thymic epithelium were largely unaffected in the majority of mutants (Fig. 2c, d). In order to substantiate the conclusion of T-cell bias of genes identified in the zebrafish screen, we analysed the tissue-specific expression patterns of their mouse homologs, and compared them to the expression patterns of genes assigned to KEGG pathways mmu04660 (T cell receptor) and mmu04155 (p53 signalling) in the BioGPS datasets which comprise more than 90 different tissues and cell types (see the “Methods” section). A substantial number of genes identified in the ENU screens exhibit high expression levels in the T cell subset of immune-related cell types (upper and lower right quadrants in Fig. 3a). This distinguishes them from the expression pattern of a random selection of genes from the genome or those associated with the p53 signalling pathway (Fig. 3b). Indeed, the patterns of expression of the genes identified in the ENU screens is comparable to the tissue-specific expression profile of genes involved in TCR signalling, yet significantly different from either p53-related or random collections of genes (Fig. 3c). Collectively, these analyses not only indicate a strong expression bias of mutant genes towards the T cell lineage, but also provide evidence for evolutionarily conserved functions of these genes, with potential relevance to the translation of our results to the mammalian T cell system.

### Cross-regulation of genes in different functional categories

**Fig. 4:**
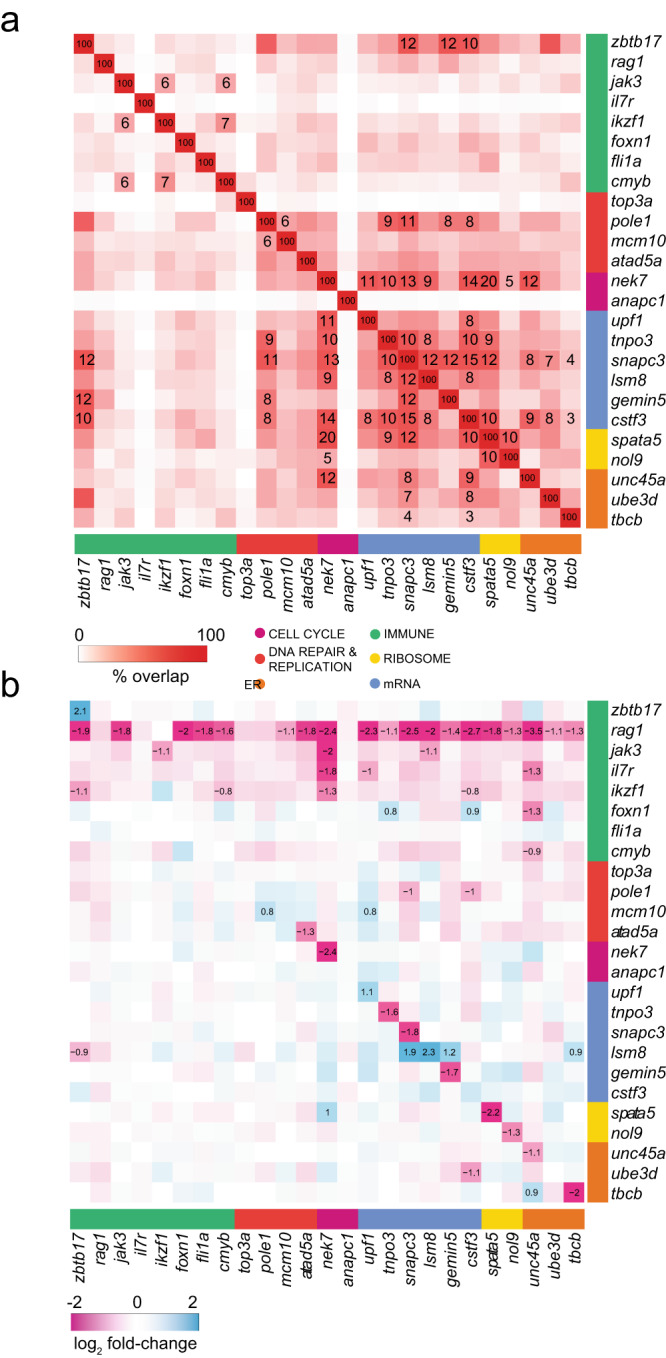
Transcriptional overlap between genetic variants and interconnected gene co-regulation. **a** Overlaps of top 1500 DEG (FDR ≤ 0.05) from each genetic variant grouped by functional categories. Only Jaccard indices with significant overlap (FDR ≤ 0.05), determined using the hypergeometric distribution, are shown; cell notes indicate the percentages of overlap. **b** Co-regulation of genes identified in the ENU screens. Genetic variants are shown in columns and their expression levels are depicted in rows; genes are grouped by functional categories. Cell notes identify genes with |log_2_ fold change | ≥ 0.5 and FDR ≤ 0.05.

Collectively, our results indicate that despite the diversity of functional categories represented in the collection of ENU mutants, the aberrations detectable in their transcriptomes converge on the T cell lineage, as read out by reduced *rag1* gene expression. We therefore examined the potential overlap of functional changes among the different variants as reflected in their transcriptomes. To this end, we determined in more detail the degrees of overlap between the 1500 most deregulated genes in the transcriptomes of 25 mutants. The resulting matrix (Fig. 4a) exhibits two key features. First, the transcriptomes of fish carrying mutations in genes assigned to the same functional category is readily apparent (see also Fig. 2b). Second, substantial similarities are revealed also among functional categories, suggesting that the genetically distinct pathways regulating T cell development may be functionally interconnected. An important prediction arising from this result is that the mutation in one gene affects the expression of (at least some) other genes identified in the screens. Our data provide evidence that this type of cross-regulation exists (Fig. 4b). For example, positive regulation of the spliceosome factor *lsm8* in the *tbcb* mutant could be the result of a feedback mechanism activated by an unfolded protein response; likewise, failure of proper mRNA processing in the case of mRNA processing mutants *snapc3* and *cstf3* could reduce expression of the gene encoding the catalytic subunit (*pole1*) of DNA polymerase epsilon. Collectively, the transcriptomes of individual mutants reveal the presence of pervasive cross-regulation both within and across functional categories, even when accounting for likely instances of destabilization of mRNAs arising from the particular structures of the mutant alleles (Fig. 4b).

### Small molecule pathway mimics

**Fig. 5:**
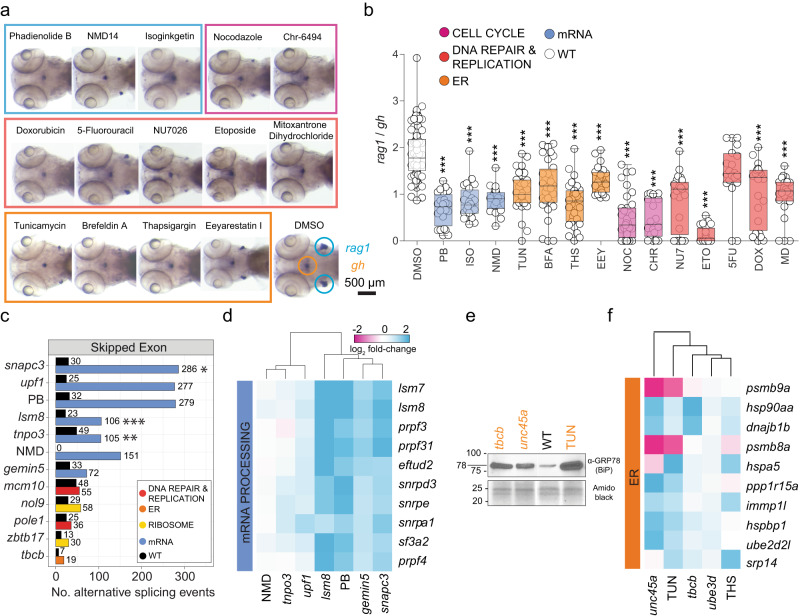
Small molecule inhibitors recapitulate genetic variant phenotypes. **a** Representative images at 5 d.p.f. after whole-mount RNA in situ hybridization with *rag1*- and *gh*-specific probes for wild-type fish treated with small molecule inhibitors targeting the major pathways impaired by mutant genes. Fish were exposed to drugs between 3 and 5 d.p.f.; DMSO-treated fish were used as negative controls. **b** *rag1*/*gh* ratios of untreated fish (DMSO) and those treated with small molecule inhibitors between 3 and 5 d.p.f. Significance was determined by one-way ANOVA with Dunnett’s post-test. *P < 0.05; **P < 0.01; ***P < 0.001. Abbreviations: 5-fluorouracil (5FU [200 μM]), brefeldin A (BFA [0.75 μM]), Chr-6494 (CHR [0.75 μM]), doxorubicin (DOX [0.75 μM]), eeyarestatin (EEY [800 μM]), etoposide (ETO [1.72 μM]), isoginkgetin (ISO [320 μM]), mitoxantrone dihydrochloride (MD [1.5 μM]), NMD14 (NMD [100 μM]), nocodazole (NOC [0.4 μM]), NU7026 (NU7 [7.5 μM]), pladienolide B (PB [0.2 μM]), thapsigargin (THS [0.5 μM]), and tunicamycin (TUN [1.5 μM]). The concentrations used are indicated in square brackets after the abbrevaitions. See source data file for b in Supplementary Data 2. **c** Effect of small molecule spliceosome inhibitor (PB) and an inhibitor of non-sense mediated decay (NMD14), on pre-mRNA processing, compared to defects in mutants. Numbers of significant skipped exon events are depicted atop each bar, as determined by reads covering exon boundaries; FDR ≤ 0.05, |inclusion level difference | ≥ 0.250) relative to wild-type siblings. *P  < 0.05; **P < 0.01; ***P < 0.001. **d** PB and NMD14 mimic the effects of mutants acting in pre-mRNA processing pathways. Gene expression patterns of top DEG known to regulate mRNA processing as defined by KEGG pathway IDs (spliceosome—dre03040, mRNA surveillance—dre03015, nonsense-mediated decay—dre03015) are depicted; genetic variants and treatment cohorts are shown in columns and DEG in rows. **e** The small molecule ER inhibitor, TUN, mimics the proteomic and transcriptional ER stress response observed in mutants. A Western blot of protein lysates of 5 d.p.f. zebrafish mutants/treatment cohorts were resolved with antibodies detecting the ER stress-related component GRP78 (BiP). Amido black staining of total protein was used as a loading control. Tunicamycin (TUN)-treated wild-type fish were included as positive control of ER stress activation. Sizes of markers are indicated in kDa. **f** Small molecule ER inhibitors, TUN and THS, mimic differential gene expression patterns in mutants affecting protein processing pathways. Gene expression patterns of top DEG known to regulate ER function as defined by KEGG pathway IDs (protein processing in ER—dre04141, proteasome—dre03015) are depicted; genetic variants and treatment cohorts are shown in columns and DEG in rows.

With a view to future therapeutic interference with the genetic pathways identified through the genetic screens, we aimed at mimicking the effects of mutations in certain ontology groups of genes by treatment of fish with known small molecule inhibitors (Supplementary Table 5). To this end, wild-type fish were treated during a 48 h-period (from 72 to 120 h.p.f.) (Fig. 5a, b), a period when the thymus is colonized by haematopoietic precursors and intrathymic T cell development begins^27,28^, to establish a dose–response relationship with respect to the thymopoietic index (*rag1/gh* ratio) (IC30 values for each small molecule inhibitor are listed in Supplementary Table 5). To examine the extent with which specific small molecule inhibitors recapitulate the mutant phenotypes, we examined two drugs in more detail. Given the prominent representation of genes important for mRNA processing, we chose pladienolide B (PB), an inhibitor of the mRNA splice regulator SF3B1, which interferes with proper recognition of intronic branch sites^29^; and NMD14, an inhibitor of nonsense-mediated decay^30^. Reassuringly, the incidence of aberrant alternative splicing events of PB-treated and NMD14-treated zebrafish is similar to those seen in the most relevant mRNA processing mutants (Fig. 5c). Moreover, the patterns of transcriptional deregulation seen in individual mutants affecting mRNA processing match the corresponding inhibitor profiles; indeed, NMD and *upf1* mutants cluster together, as do PB and the *lsm8*, *gemin5* and *snapc3* mutants (Fig. 5d). In a second confirmatory study, we found that similarly to *tbcb* and *unc45a* mutants, tunicamycin, an inhibitor of glycosylation in the ER^31^, and thapsigargin (THS)^32^, induced ER stress and shared transcriptional profiles at the level of pathway deregulation (Fig. 5e, f).

### Interconnected network of genes and pathways

**Fig. 6:**
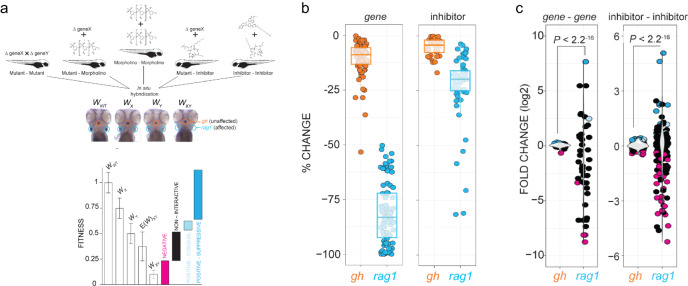
Analysis of interactions. **a** Schematic of the types of experiments underlying the interaction network. The integrated interaction screen consists of mutant–mutant, mutant–morpholino, morpholino–morpholino, mutant–inhibitor, and inhibitor–inhibitor interactions, where the numbers of T cells as determined by *rag1* gene expression relative to growth hormone (*gh*)-expressing somatotrophic epithelial cells is normalized to wild-type levels (*W*_WT_; fitness). Single mutant (*W*_x_, *W*_y_) and double mutant fitness (*W*_*xy*_) values were determined by normalization of their *rag1*/*gh* ratios to wild-type *rag1*/*gh* ratios. The expected double mutant fitness *E*(*W*_*xy*_) is the product of single mutant fitness values (*W*_*x*_ × *W*_*y*_). A non-interactive line is assigned to an observed double-mutant fitness value that is within the propagated error of expected double-mutant fitness. Negative interaction is called when an observed double-mutant fitness is significantly less than the expected double-mutant fitness minus the propagated error. Positive–coequal interaction is called when an observed double-mutant fitness is significantly greater than the expected double-mutant fitness plus the propagated error, but equivalent to the least fit single mutant. Positive–suppressive interaction is called when an observed double-mutant fitness is significantly greater than the expected double-mutant fitness plus propagated error and greater than the least fit single mutant. **b** Effect of gene mutations (mutants and morphants) and inhibitor treatments on two different tissues, pituitary gland (as determined by *gh* expression) and T cell (as determined by *rag1* expression). The changes in expression levels in percent relative to genetically wild-type (panel designated gene) or untreated controls (panel designated inhibitor) are given for *rag1* and *gh* hybridization signals (cf., Figs. 4 and  5). For both types of analyses, the differences between *gh* and *rag1* expression levels are significant at *P* < 0.001 (two-tailed Student´s *t*-test). **c** Effect of gene–gene genetic interactions (mutant–mutant) and inhibitor genetic interactions (inhibitor–inhibitor) on two different tissues, T cell (*rag1*) and pituitary gland (*gh*). Relative log_2_-fold changes between observed (*W*_*xy*_) and expected double mutant fitness *E*(*W*_*xy*_) values are given for T cells and growth hormone-producing somatotropic cells. Colours represent the interaction types (black, non-interactive; magenta, negative; light-blue, positive-coequal; dark blue, positive-suppressive). *P* values were determined by two-tailed Student´s *t*-test. Statistical tests for homogeneity of variances were performed using Bartlett’s test.

To substantiate the conclusion of cross-regulation more fully, and to establish the structure of the underlying genetic network, we performed pairwise interaction analyses for selected mutants and small inhibitors. We define fitness of a cell type as the strength of the RNA in situ hybridization signal; for T cells, *rag1* expression; for cells in the hypophysis, *gh* expression. Wild-type values are assigned a fitness value *W* of 1, whereas the values for the two experimental (mutant or inhibitor-treated) conditions are defined as *W*_*x*_ and *W*_*y*_, respectively. Under the multiplicative model (see the “Methods” section for details), we calculated an expected fitness, *E*(*W*)_*xy*_, for the combination of the two conditions by calculating the product of the two single fitness values, and compared this value to the observed fitness *W*_*xy*_ (Fig. 6a). The results are interpreted as follows. Non-interactive interactions are defined as an observed double-mutant fitness not significantly different from the expected fitness of the double-mutant (range of black values in the schematic of Fig. 6a); negative interactions are defined as an observed double-mutant fitness significantly less than the expected fitness of the double-mutant (range of red values in Fig. 6a); positive-coequal interaction is an observed double-mutant fitness significantly greater than the expected fitness of the double-mutant, but equivalent to the least fit single mutant (range of light blue values in Fig. 6a); positive-suppressive interaction is an observed double-mutant fitness significantly greater than the expected fitness for the double mutant and the least fit single mutant (range of dark blue values in Fig. 6a). As expected, the diminished individual fitness values of T cells of genetic variants and of inhibitor-treated animals are reflected in significant median signal intensity reductions for *rag1* expression (−80.9% and −19.8% [IC30 target], respectively), as opposed to *gh* signals produced by somatotrophic epithelial cells, which remain essentially unchanged (Fig. 6b).

In order to ascertain that the cell-type specificity is maintained under the conditions of gene–gene and inhibitor–inhibitor interactions, we calculated the fold changes of *gh* and *rag1* gene expression levels. As expected, the great majority of interactions between genes and small molecule inhibitors, respectively, had minimal effects on *gh-*expressing cells in the hypophysis. By contrast, the *rag1* expression levels varied widely, as a result of strong positive and negative interactions (Fig. 6c; Supplementary Data 1).

### Identification of off-target effects in interaction studies

**Fig. 7:**
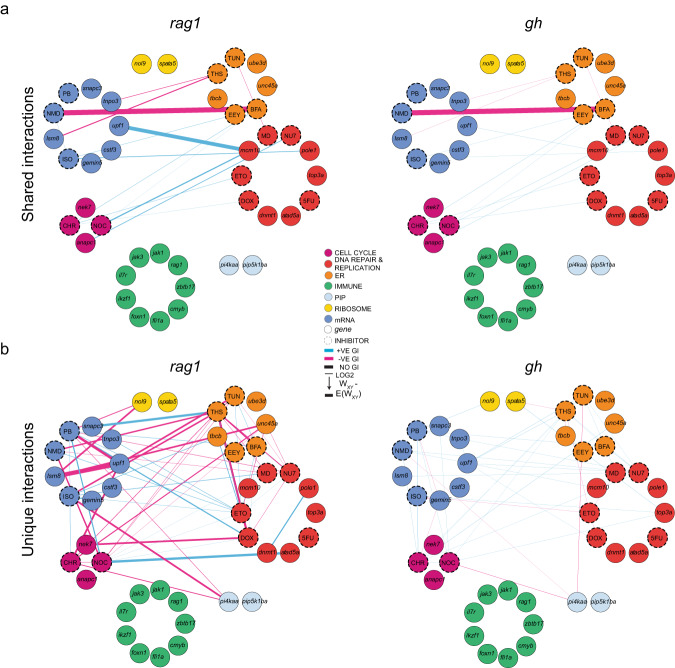
Common and tissue-specific genetic interaction networks. **a** Shared genetic interactions between mutant genes and inhibitors affecting fitness of both T cells (*rag1*) and growth hormone-producing somatotropic cells (*gh*). Line font size reflects relative log_2_-fold changes between observed and expected double mutant fitness values. Nodes are grouped by primary biological pathways that are affected by mutations or inhibitors. Solid or dashed circles around each node denote either genes or inhibitors, respectively. For this presentation, positive–suppressive and positive–coequal interactions are combined. Colours represent interaction types (magenta, negative; blue, positive; black, non-interactive). 5FU 5-fluorouracil), BFA brefeldin A, CHR Chr-6494, DOX doxorubicin, EEY eeyarestatin, ETO etoposide, ISO isoginkgetin, MD mitoxantrone dihydrochloride, NMD NMD14, NOC nocodazole, NU7 NU7026, PB pladienolide B, THS thapsigargin, and TUN tunicamycin. **b**, Cell type-specific genetic interactions between mutant genes and inhibitors specifically affecting fitness of T cells.

The separate analysis of *gh* and *rag1* expression levels readily reveals whether interactions between genes or inhibitors affect only T cells, or both T cells and growth hormone-producing cells in the hypophysis, the latter situation being indicative of potential off-target effects. For example, combining NMD14 + brefeldin A (BFA) inhibitors is toxic to not only T cells (Fig. 7a, left panel), but also to *gh*-producing somatotrophs (Fig. 7a, right panel), an outcome which was not expected from the individual effect of two inhibitors (Fig. 5a, b). Indeed, such off-target effects can only be detected in the context of the whole organism, illustrating the advantage of this approach over cell-based screens. Many combinations of genetic variants and/or drugs exhibited T cell-specific toxicity, such as seen with THS+ etoposide or etoposide + doxorubicin (Fig. 7b, left panel), while neither of these drug combinations exhibited off-target effects in *gh*-producing cells (Fig. 7b, right panel). Collectively, the transcriptional landscapes of mutants, the T cell expression signatures of mutant genes, and the tissue-restricted effects of genetic interactions provide further support for the notion that the development of T cells is controlled by several interconnected pathways.

### Multiparametric genetic interaction network

**Fig. 8:**
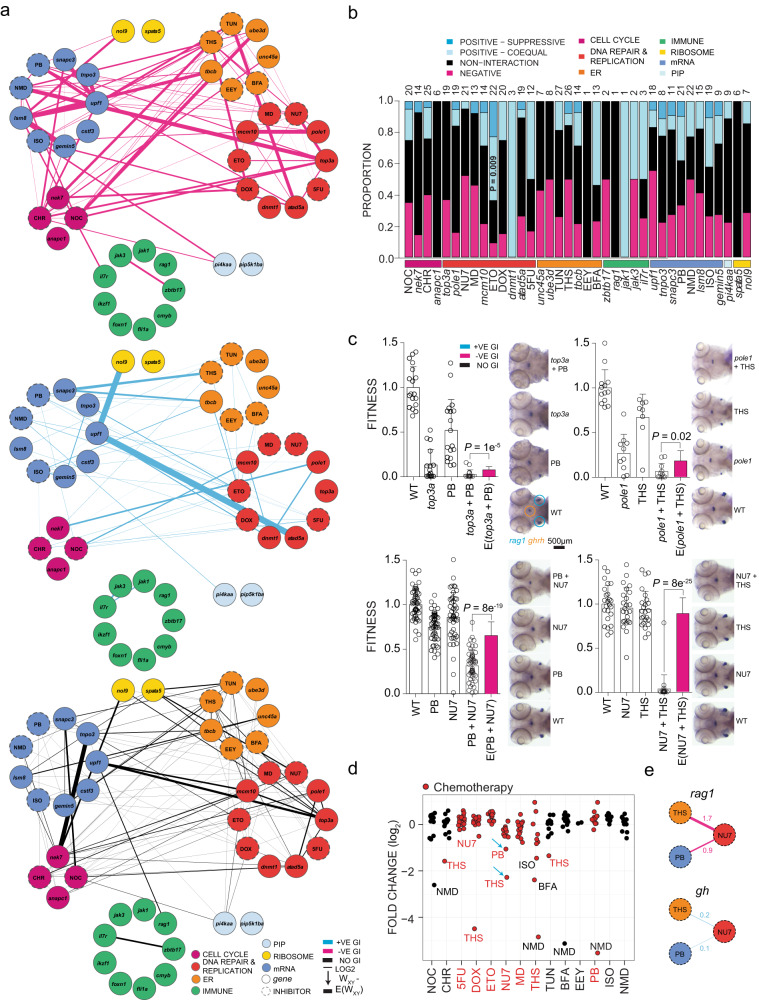
Genetic landscape of developing T cells in zebrafish. **a** Genetic interactions between genes and inhibitors affecting fitness of T cells (*rag1*) normalized to fitness of growth hormone-producing somatotropic cells (*gh*). Line font size reflects relative log_2_ fold-changes between observed and expected double-mutant fitness values for *rag1/gh* ratios. Nodes are grouped by primary biological pathways affected by mutation or inhibitor. Solid or dashed circles around each node denote either genes or inhibitors, respectively. For this presentation, positive–suppressive and positive–coequal interactions are combined. Colours represent interaction types (magenta, negative; blue, positive; black, non-interactive). **b** Proportions of interaction types by genes and inhibitors, grouped by biological functional categories. The number of interactions per node, including non-interaction, is depicted atop each bar. *P* values for genetic interaction type composition deviating from the average of all interactions were determined by Fisher’s exact test. **c** r*ag1*/*gh* ratios (fitness) normalized to wild-type for genetic variants and inhibitor treatments. The observed double mutant fitness values are compared to the expected double mutant fitness values (*E*); negative genetic interactions between ER–mRNA processing and ER–DNA are independent of experiment types (mutant–inhibitor or inhibitor–inhibitor). *P* values were determined by two-tailed Student’s *t*-test. **d** Genetic interaction between inhibitors affecting fitness of T cells. Relative log_2_-fold changes between observed and expected double mutant fitness values for *rag1/gh* ratios. Inhibitor combinations denoted with blue arrow were tested in adolescent fish and in the tumour model. **e** Discordant cell type-specific genetic interactions between inhibitors affecting fitness of T cells (*rag1*) and growth hormone-producing somatotropic cells (*gh*). The identification of cell type-specific strongly negative interactions informs choices for in vivo treatment of T-ALL. 5FU 5-fluorouracil, BFA brefeldin A, CHR Chr-6494, DOX doxorubicin, EEY eeyarestatin, ETO etoposide, ISO isoginkgetin, MD mitoxantrone dihydrochloride, NMD NMD14, NOC nocodazole, NU7 NU7026, PB pladienolide B, THS thapsigargin, and TUN tunicamycin.

In the next step, we expanded our analysis to all five types of interactions; the contributions of some mutants were replaced by knock-downs using anti-sense morpholino oligonucleotides to facilitate the analyses. The results from mutant–mutant, mutant–morphant, morphant–morphant, mutant–inhibitor, and inhibitor–inhibitor interactions were incorporated into a single network built from 284 pairwise interactions (Supplementary Data 1). In 47% of all pairs tested, no interaction could be detected; positive interactions were seen in 23.1% of cases, which comprised positive–coequal (18.3%) and positive–suppressive (4.8%) outcomes; negative interactions were found in 29.9% of all tests (Fig. 8a; Supplementary Data 1). Individual components of the DNA repair and replication ontology group were enriched for positive interactions, when compared against the overall average; for instance, for etoposide, 12 of 22 interactions were scored as positive (*P* = 0.009) (Fig. 8b). Despite their functional heterogeneity, an enrichment for positive interactions was detectable at the level of some ontology groups; for instance, between cell cycle and DNA repair/replication groups (27%, *P* = 0.29, *n* = 26 interactions) (Fig. 8b; Supplementary Fig. 5). Mechanistically, we attribute these alleviating outcomes to the improved error correction capacity when the cell cycle is slowed down, thereby avoiding genomic catastrophe^33,34^. Negative interactions were apparent between ER and cell cycle (47%, *P* = 0.1, *n* = 15) ontology groups (Fig. 8b, Supplementary Fig. 5), an outcome reflected in clinical observations that proteasome inhibitors sensitize cells to genotoxic agents^35^. In our present experiments, we tested only a subset of all possible two-way interactions; hence, when the interactions matrix is expanded in future studies, we expect that more biologically relevant interactions will be revealed between genes and drugs, both individually and at the level of functional groups.

### Interference with larval T cell development

In our attempt to design new types of T cell cancer therapies, we focused on the identification of inhibitor–inhibitor interactions that mimicked the outcome of gene–gene interactions. In order to exclude off-target effects on somatotrophs, we proceeded in a stepwise fashion, starting with gene–inhibitor interactions. By way of example, we treated the *top3a* DNA replication/repair mutant with the pre-mRNA processing modulator PB^36^. Using the *rag1/gh* ratio as a measure of fitness (i.e., thymopoietic capacity), this gene–inhibitor combination revealed a negative interaction affecting only T cells; note the reduced *rag1* signal and the unchanged signal for *gh* in the representative RNA in situ hybridization panels (Fig. 8c). We likewise treated the *pole1* DNA replication/repair mutant with the ER stressor THS^32^, and we again observed a negative interaction (Fig. 8c). We then replaced the contribution of each mutant in these combinations with an inhibitor of DNA-dependent protein kinase, NU7026 (NU7; ref. ^37^), a drug that targets the same ontology pathway as *top3a* and *pole1* (DNA replication/repair). Both combinations, PB + NU7 and NU7 + THS, respectively, elicited strong synthetic lethality (Fig. 8c) of developing T cells. When this type of drug–drug combination screen was carried out in a systematic fashion, a substantial number of strongly negative combinations were identified (Fig. 8d). Analysis of the effects of the two drug combinations on *rag1* and *gh* expression individually indicated a strong negative (toxic) effect on the former, whereas the latter was distinguished by a mild positive effect (Fig. 8e).

### Interference with T cell development in adolescent fish

**Fig. 9:**
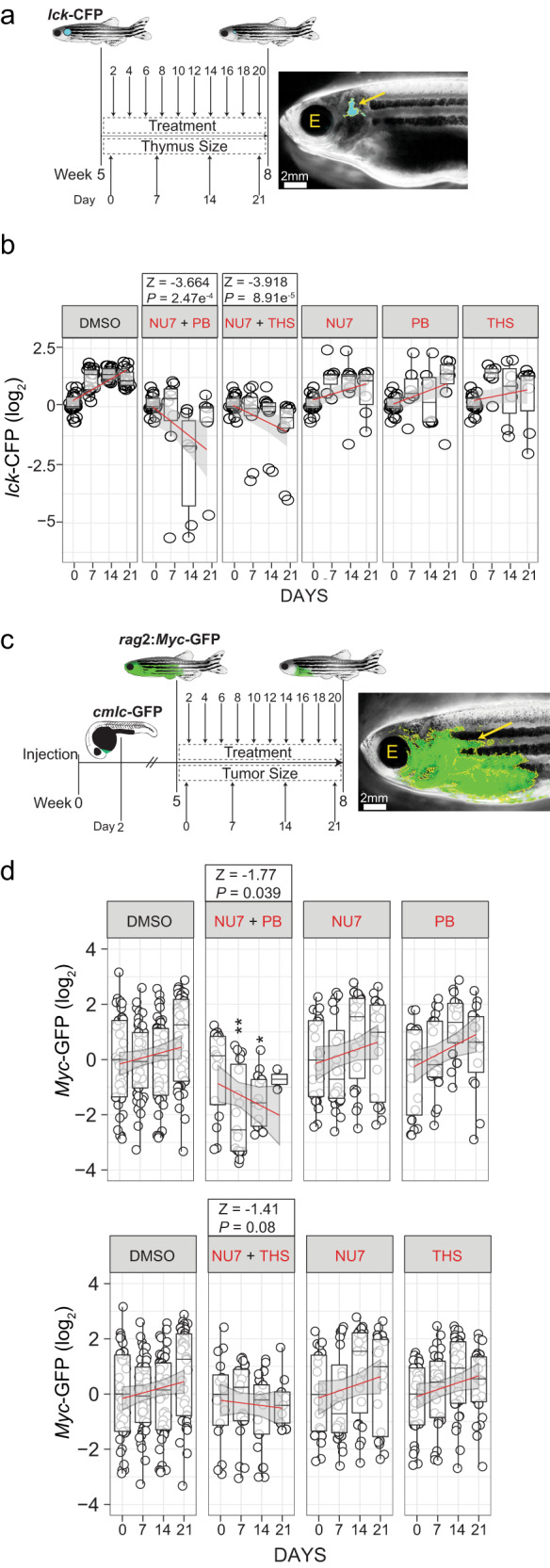
Exploiting synthetic lethality in T cells for T-ALL treatment. **a** Treatment schedule of adolescent *lck*-CFP zebrafish (5 weeks of age) with the indicated inhibitor combinations. The thymus size relative to fish size was measured weekly. Representative dark field image of a zebrafish with the *lck*-CFP fluorescent signal overlay (yellow arrow and outline). The position of the eye is labelled for orientation (E). **b** Thymus size during combination treatment of adolescent *lck*-CFP zebrafish. The sizes of thymi are expressed as log_2_-fold changes relative to the sizes at day 0 of treatment. The significance of differences between day 0 and days following treatment was determined by two-way ANOVA, for repeated measurements with Bonferroni post-test. A *z* statistic was used to compare regression coefficients between treated and untreated groups. See source data file for Fig. 9b in Supplementary Data 2. **c** Treatment schedule of adolescent *rag*2:*Myc-*GFP–injected zebrafish (5 weeks of age) with inhibitor combinations, following the onset of T-ALL. *Rag*2:*Myc-*GFP and *cmlc-*GFP containing plasmids were co-injected into wild-type fish at the one-cell stage and sorted for heart expression of the *cmlc*-GFP reporter at 2 d.p.f. as an indicator of successful transgenesis. Transgenic fish were grown until the age of 5 weeks and then sorted for onset of T-ALL using *rag2*:*Myc-*GFP. Tumor sizes relative to fish size were measured weekly. Representative dark field image of a 5 week old zebrafish with *rag*2:*Myc-*GFP fluorescent signal overlay (yellow arrow and outline). **d** T-ALL tumor sizes following combination inhibitor treatment of adolescent *rag*2:*Myc-*GFP. Tumor sizes are expressed as log_2_-fold changes relative to day 0 of treatment. *P < 0.05; **P < 0.01. See source data file for Fig. 9d in Supplementary Data 2.

Since we had integrated the effects and interactions of small molecule inhibitors into the genetic landscape of larval T cell development, it was important to verify the general properties of this network at later stages of development. To this end, we again evaluated the NU7 and PB, and NU7 and THS inhibitor combinations. NU7 and its derivatives have shown activity in in vivo tumour models^38^, whereas PB^39^ and THS (the latter in the form of its G-202 prodrug^40^) have already been used individually in clinical trials. However, to the best of our knowledge, the synthetic effects of these two drug combinations on T cell development have not yet been described. To confirm the activities of the inhibitors in adolescent fish, when the thymus has fully matured, we used a *lck:CFP* transgenic fish line^41^ to mark T cells with a fluorescent reporter throughout life (Fig. 9a). Although the relative efficiencies of the two combinations differed from the larval situation (Fig. 8c, d), a robust synthetic lethality was observed for both NU7 + PB and NU7 + THS combinations when compared to the appropriate single inhibitor controls (Fig. 9b).

### Synthetic lethality for T-ALL therapy

Next, we set out to explore the efficacy of these inhibitor combinations for the treatment of T-ALL, which arises from immature T cells^13,14^. To this end, we used an established zebrafish model of T-ALL, where tumour development is driven by the expression of the mouse *c-Myc* gene under the control of the zebrafish *rag2* promoter^42^ (Fig. 9c); this model was chosen, because the c-Myc gene is commonly upregulated in *Notch1*-dependent T-ALL^14^, which represents one of the major groups of human T-ALLs^43^. For this experiment, the concentrations of inhibitors were chosen such that they would not inhibit the growth of the tumours when used alone. However, when combinations were used, a significant degree of tumour regression was induced; this synthetic lethality is most pronounced in the NU7 + PB combination (Fig. 9d). Collectively, these experiments indicate that the effects of mutations and drugs on the progression of T-ALL can be predicted from their effects on normal T cell development at larval and adolescent stages of development.

## Discussion

Our study reports the results of large-scale genetic screens aimed at identifying important nodes in the genetic network governing T cell development in zebrafish larvae. This animal model was chosen for several reasons. First, a large body of work indicates that the general principles that underpin the haematopoietic and immune systems of fish and mammals are very similar^44^. Second, because of their high fecundity, and the low cost of maintenance, genetic screens in zebrafish are an attractive alternative to conducting genetic screens in mice, which require substantially larger infrastructures and man-power^45,46^. When a primary screen is subsequently extended to the complex mating schemes required to establish the structure of the genetic network(s) underlying a particular phenotypic trait, the advantage of using the zebrafish model becomes particularly relevant.

The present genetic screens had three main goals. The primary aim was the identification of genes that affect the development of larval T cells in a tissue-specific or a tissue-restricted fashion, and by way of design, specifically excluding genes with pleiotropic modes of action. Although we did not expect to identify the full complement of the genes exhibiting the desired characteristics, the screen was nonetheless of sufficient magnitude to uncover at least one of the components of each of the major pathways underlying T cell development. The fact that we recovered two alleles each for two of the 33 genes identified here indicates that we approached our primary goal. Upon further characterization of the 33 genes thus identified, 29 could be assigned to 7 distinct developmental and/or cell biological pathways. An unexpected outcome of our work was that mutations in several genes that are known for fundamental and ubiquitous cellular functions, such as DNA replication, give rise to tissue-restricted phenotypes. The observation that these variants predominantly affected the development of T progenitors, while largely sparing other cell types, might (at least partially) be explained by the fact that such variants often encoded hypomorphic rather null alleles, potentially affecting only a subset of functionalities in these multi-domain proteins. The sensitivity of lymphoid lineages and tissues to these genetic aberrations may additionally arise from the tissue-specific patterns of genes encoding co-factors, the functional redundancy arising from the plasticity of relevant protein complexes, and/or the disruption of dedicated signalling processes^47–50^. Our findings thus support the notion emerging from studies in mice and humans of unexpected immunological roles for numerous genes involved in core biological processes, many with no prior association with lymphopoiesis^51^. Our results thus support the widely held view that one of the main advantages of forward genetic screens lies in the discovery of subtly modified proteins, whose tissue-specific functions may be missed by complete or tissue-specific gene inactivation that are at the heart of reverse genetic approaches. This favourable outcome is illustrated by the identification of the *dnmt1*^t25501^ allele, which revealed a lymphoid lineage-specific function of maintenance methylation in both zebrafish^22^ and mouse^23^, whereas the null allele is embryonic lethal^52^.

A second goal of our screen was the identification of functional interdependencies among the individual genetic variants. An early indication that the identified variants may be part of a common network structure was revealed by the analysis of their transcriptomes, which identified a substantial overlap between differentially expressed genes. Substructures in this network often mirrored the presumed functional groups assigned to variants based on prior knowledge. Remarkably, we found it possible to pharmacologically mimic the inherent intolerance of T cells to defects in certain biological pathways, a result that emerged after the expansion of the interaction screen to include a select number of small molecule inhibitors. In this way, alleviating (positive) and synthetic (negative) interactions could be replicated using only a small number of well-established small molecule inhibitors. This finding set the stage to exploit synthetic lethal interdependencies in our network to specifically target developing T cells, and their malignant counterparts, the third aspect of our study goals.

The successful development of a synthetic lethal strategy applicable to the interference with T cell development in vivo critically depends on the exclusion of undesired off-target effects. Indeed, it is here, where the advantage of an organismal level genetic interaction screen becomes most relevant. Whereas screens based on cell lines allow high-throughput screens for secondary and even tertiary interactions, they invariably suffer from the problem that a particular cell line may represent only one type of a developmental pathway or tissue, and, by design, is agnostic to effects on other cell types. Synthetic lethality has been considered as an effective strategy to specifically target tumours carrying cancer-associated somatic mutations; however, the existence of genetically and phenotypically distinct subpopulations within each tumor undermines the effectiveness of these approaches^12^. Genes sharing synthetic lethality are often derived from a common genetic network of many cell lines^5^. Whilst this improves target confidence and potentially expands treatment utility to multiple cancer types, it could increase the prospect of unintended side effects. Moreover, despite the considerable breadth of synthetic lethality screens, few treatments targeting these interactions have proven clinically useful^11,53^; possible reasons for which include cell type differences, an inability to replicate in vitro findings in the context of an entire organism, and a failure to capture non-cell autonomous effects^54–56^. Although genetic interaction screens in vertebrates are not amenable to high-throughput procedures, they may yield useful information, when focused on a specific cell type. As illustrated here, the structure of genetic networks of normal cells established in the context of an entire organism suggests synthetic lethal drug combinations, whose efficacy against corresponding malignancies is independent of mutation status and thus overcomes the confounding effects of pervasive tumour heterogeneity.

In conclusion, the identification of key nodes in the genetic network of zebrafish T cell development will provide the basis for further studies both into the structure and function of sub-circuits regulating particular aspects of T cell differentiation, as well as investigations into the evolutionary conservation of network structure. Finally, it will be worthwhile to examine in pre-clinical studies some of the drug combinations that were found here to be useful in interfering with the progression of T-ALL.

## Methods

### Animals

The zebrafish (*Danio rerio*) strains Ekkwill (EKK), Tüpfel long fin (TL), wild-type-in-Kalkutta (WIK), AB, Assam (ASS), and Tubingen (TU) were maintained in the animal facility of the Max Planck Institute of Immunobiology and Epigenetics. The *ikzf1-GFP* transgenic zebrafish^27,28^, the *lck:CFP* transgenic zebrafish^41^ and the *rag1* mutant^24^ were described previously. The characterization of the *rag2*:*Myc*-GPF transient leukaemia model was also described previously^42^. All animal experiments were approved by the institute’s review committee and conducted under licenses from the local governments (Regierungspräsidium Freiburg [AZ 35-9185.81/G-19/69; AZ 35-9185.81/G-14/41; AZ 35-9185.81/G-17/79; AZ 35-9185.81/G-13/70]; Regierungspräsidium Tübingen [AZ AP1/02]).

### ENU mutagenesis

A detailed description of the forward genetic screen design, coverage, complementation analysis, and mutant identification by positional cloning and whole genome sequencing can be found in refs. ^19,20^. The functional relevance of the *ikzf1* mutation in the II032 mutant was confirmed by complementation analysis with a previously identified *ikzf1* mutation (t24980; ref. ^19^).

### Whole genome sequencing

For genomic libraries of mutant lines, 20–100 mutants at 5 d.p.f. embryos (as judged by reduced *rag1* signals after RNA in situ hybridisation) from 4 different mating pairs were pooled; the exact number of biological replicates per genotype is reported in the data files associated with NCBI Sequence Read Archive (SRA) project PRJNA622735. The combined analysis of at least 20 mutant embryos ensures that the signals emanating from an occasional mis-sorted animal does not affect the overall analysis for sequence polymorphisms. After purification of genomic DNA, 6 μg were sheared to ~300 bp fragment size using a Covaris S220 sonicator. Sonication was followed by a clean-up step by adding an equal volume of Agencourt AMPure XP magnetic beads (Beckman Coulter). Paired-end libraries were constructed with the NEXTflex PCR-free DNA Sequencing Kit (Bioo Scientific, Cat# NOVA-5142-02) according to the manufacturer’s instructions. Library quality controls included assessment of size distribution using an Agilent Bioanalyzer, and determination of DNA concentration using a KAPA Library Quant Illumina Kit (Peqlab, Cat# KAPBKK4854). Sequencing was carried out in the paired-end 100 bp run mode of an Illumina HiSeq 2500. Wild-type genomic libraries were made from the original adult males used in the generation of the ENU-mutagenised lines, as well as from five different in-house wild-type strains (TUE, WIK 7, WIK 11, ASS, TLEK). The genomic DNAs for the ENU-mutant libraries were purified from frozen somatic tissues of the original adult males. To generate the genomic libraries of the in-house wild-type strains, 25–50 embryos from each line were sequenced. Sequencing reads of each sample were mapped to the Zv9.70 reference genome using the Bowtie 2 programme^57^. Mapping duplicates were removed using Picard Mark Duplicates in Galaxy^58–61^. In order to identify candidates for the causal mutation, without previous knowledge of the linked region classically obtained by generating outcrosses and subsequent positional cloning after mapping of meiotic recombination events, mutant lines were subjected to a sequential filtering of genetic background and wild-type SNPs. Mutant SNPs were called using Mpileup version 1.1.1 from SAM tools. The Mpileup file was subjected to two sequential steps of SNP filtering using two bulk Mpileup files generated by pooling: the six wild-type lines (ENU, Tue, Wik7, Wik11, ASS, TLEK), and the respective mutant lines with unknown genetic lesions. Filtering constraints for SNPsift included, (DP > 5) & (DP < 100) & (QUAL > = 40) & (DP4[2] > 0) & (DP4[3] > 0)! (REF = ‘N’)! (ALT = ‘N’). The filtering was performed using the SNP Intersector/Substractor tool^62,63^ from MegaMapper^64^. The flagged nucleotide changes in the output file were evaluated for their functional effect using SNPEff^65^. From that list, only homozygous SNPs were kept, using Filter from Galaxy tools version 1.1.0. The resulting lists consisted of homozygous nonsense and missense mutations throughout the genome, not present in any of our wild-type lines or other mutant lines. The identified mutation was visualised on IGV (Integrative Genomic Viewer)^66,67^ using the bam files of mutants and wild-type strains in parallel.

### RNA in situ hybridization

Procedures for RNA in situ hybridisation probes and determination of *rag1*/*gh* ratios were described previously^19^. Briefly, the areas of *rag1* and *gh* signals were measured using ImageJ from photographs taken using the Leica MZFLIII stereomicroscope with the Leica DFC300 FX camera. An average of *rag1* signals from the two thymic lobes was calculated and normalised to the *gh* signal to obtain the *rag1*/*gh* ratio as a measure of thymopoietic capacity. RNA in situ hybridisations were also performed on embryos from an in-cross of heterozygous carriers for detection of defects in other haematopoietic lineages including, thymocytes (5 d.p.f.—*ikzf1*) and thymic epithelial cell (5 d.p.f.—*foxn1*), haematopoietic stem cell (36 h.p.f.—*c-myb*, *runx1*), lymphoid (24 h.p.f.—*ikzf1*), myeloid (24 h.p.f.—*spi1b*), erythrocyte (24 h.p.f.—*gata1*), neutrophil (24 h.p.f.—*mpx*), macrophage (24 h.p.f.—*lcp1*)^24,28,68,69^. Images of whole mounts were taken using the Zeiss Axioplan2 microscope with the AxioCam MRc5 camera. Z section images were captured using Zeiss’ Zen Software and focused stacked in Adobe Photoshop CS6.

### Morphants

Morpholino antisense oligonucleotides (morpholinos) targeting the sequences of either initiation codons (to block translation of both maternal and zygotic mRNAs), or splice donor and/or acceptor sites (to block processing of zygotic mRNAs; leaving processed maternal mRNAs intact) of target gene mRNAs were designed by and sourced from GeneTools, LLC (Supplementary Table 1). Lyophilised morpholinos were resuspended in nuclease-free water at a concentration of 1 mM and stored at 4 °C. Morpholinos diluted in 1× Danieau buffer (58 mM NaCl, 0.7 mM KCl, 0.4 mM MgSO_4_, 0.6 mM CaCl_2_, 5 mM HEPES, pH 7.6) were titrated and injected in a volume of 1–2 nL into wild-type embryos at the 1-cell stage as described previously^20^. The phenotypes of morphants were determined by RNA in situ hybridisation, comparing the *rag1*/*gh* ratio of injected versus un-injected fish at 5 d.p.f.

### Phenotypic rescue

Recombinant clones containing full-length cDNAs or bacterial artificial chromosomes (BAC) encompassing the genes of interest (Supplementary Table 2) were obtained from Source BioScience. Clones were cultured in antibiotic-containing Luria-Bertani (LB) broth specific to each vector at 37 °C overnight. Plasmids were subsequently extracted from bacteria using Plasmid Midi Kit (QIAGEN, Cat# 12143). Preparation of mRNA from cDNA clones was performed by in vitro transcription using mMESSAGE mMACHINE T7, T3 and SP6 Kits (ThermoFisher Scientific, Cat# AM1344, AM1348, AM1340) using 1 μg of plasmid DNA, linearised using vector-specific restriction enzymes at positions distal to the relevant promoter sequence. Transcribed mRNA was precipitated using LiCl and EtOH, resuspended in nuclease-free water and stored at −80 °C. BACs were also grown from clones cultured in antibiotic-containing LB broth, but plasmids were extracted using Large Construct Kits (QIAGEN, Cat# 12462). Purified mRNAs and BACs were titrated (50–400 ng/μL) and injected in a volume of 1–2 nL into 1-cell stage embryos resulting from an in-cross of heterozygous mutant carriers as described above. The phenotypes of morphants were determined by performing RNA in situ hybridisation, comparing the *rag1*/*gh* ratio of injected versus uninjected mutant fish at 5 d.p.f.

### CRISPR mutants

CRISPR guide RNAs were created by incubating overlapping primers (Supplementary Table 3) (5 μg/primer, 100 mM MgCl_2_, 0.1 M Tris pH 7.5) at 95 °C for 5 min and cooling to RT. Annealed primers were ligated into BsaI-digested pDR274 vector (50 ng annealed primers, 10 ng BsaI-digested pDR274, 5 U T4 ligase, 1× T4 ligase Buffer) for 2 h at 22 °C, with the reaction inactivated at 65 °C for 10 min. The ligation mixture was dialyzed and transformed into *E.coli* DH5α by electroporation. Culture of transformants, plasmid extraction and in vitro transcription of guide RNAs were carried out as described above. Purified CRISPR guide RNAs were tested for specificity by in vitro digestion of target DNA (80 ng PCR amplicon containing target sequence, 600 ng Cas9 protein from *Streptococcus pyogenes* [PNA Bio], 300 ng guide RNA, 1× CutSmart buffer [New England Biolabs]) at 37 °C for 1 h. Guide RNA was removed by adding 4 μg of RNAse A to the reaction for 15 min at 37 °C prior to visualization of cleavage products by agarose gel electrophoresis. The extent of in vitro digestion of target DNA was compared between reactions with and without addition of guide RNA. A single guide RNA was designed and used for each target gene to create a frameshift genetic lesion. CRISPRs were titrated and injected in a volume of 1–2 nL into wild-type embryos at the 1-cell stage in a solution containing (250 ng/μL guide RNA, 500 ng/μL Cas9 protein, 1% phenol red, Danieau buffer). Carriers of genetic lesions were outcrossed to wild-type fish, and stable carrier lines were created from suitable frameshift mutations. RNA in situ hybridisation was performed on an incross of heterozygous carriers for the genetic lesion to compare *rag1*/*gh* ratio of homozygous mutant versus homozygous wild-type fish at 5 d.p.f. The detailed characterization of the *ikzf1* and *foxn1* crispants will be described elsewhere.

### RNA extraction and cDNA synthesis

Individual zebrafish embryos (5 d.p.f.) from an in-cross of heterozygous carriers were homogenised in 100 μL of Tri Reagent (Sigma, Cat#93289) and transferred to 2 mL deep 96-well plates containing an additional 400 μL of Tri Reagent. The RNA-containing aqueous phase was stored at −80 °C until genotyping was completed. DNA was extracted from the interphase and organic phase according to the manufacturer’s instructions and genotyping was performed using the primers listed in Supplementary Table 4. Following genotyping, RNA was extracted from homozygous mutants and homozygous wildtype siblings. DNA was removed from RNA extraction using TURBO DNA-free kit (Invitrogen, Cat#AM1907). RNA was quantified using the Qubit RNA HS Assay Kit (ThermoFisherScientific, Cat#Q32852) and the Qubit 4 Fluorometer (ThermoFisherScientific, Q33226). RNA quality was checked by determining the 18S/28S rRNA ratio using the Fragment Analyzer RNA Kit (ThermoScientific, Cat#DNF-471-0500) and the 5200 Fragment Analyzer System (ThermoScientific, Cat#M5310AA). cDNA libraries were prepared from 1 μg of mRNA following poly-A selection using TruSeq stranded mRNA Library Prep (Illumina, Cat#20020595) according to manufacturer’s instructions.

### RNA sequencing and computational analysis of RNA-seq data

RNA-Seq was performed using mutant and wild-type siblings from each zebrafish line (*n* = 2–6; the exact number of biological replicates is reported in the files associated with NCBI Gene Expression Omnibus (GEO) project GSE147555). The libraries were sequenced in paired-end 75 bp mode at a depth of 25 million reads on an Illumina HiSeq 2500/3000 instrument. Reads were aligned to the reference genome with STAR 2.5.2b-1 (ref. ^70^) and the reference annotation from Ensembl (Zv10.85, http://www.ensembl.org/info/data/ftp/index.html). The resulting alignments were quantified at the gene level with featureCounts version 1.6.0.1 (ref. ^71^) and differential expression performed using DESeq2 version 2.11.40.1 (ref. ^72^). The analysis was orchestrated on the in-house version of the Galaxy server based on the Galaxy platform^58^. All tools were used with default parameters unless otherwise stated.

### Pathway enrichment analysis

Pathway enrichment analysis of differentially expressed genes was performed using ClusterProfiler^73^. A detailed description of data processing can be found at https://github.com/connoromeara/OMeara_et_al_2021. Briefly, KEGG enrichment analysis was performed on the top 1500 differential expressed genes (DEG) (FDR ≤ 0.05) from each genetic variant. Genes regulating T cell development and differentiation (T cell receptor—mmu04660, primary immunodeficiency—mmu05340, notch signalling—mmu04330), DNA synthesis (DNA replication—dre03030, MMR—dre03430, BER—dre03410, HR—dre03440, p53 signalling—dre04115), cell cycle (apoptosis—dre04210, cell cycle—dre04110, cellular senescence—dre04218), mRNA processing (spliceosome—dre03040, mRNA surveillance—dre03015, nonsense-mediated decay—dre03015), ribosome function (ribosome biogenesis—dre03008, ribosome—dre03010) and ER function (protein processing in ER—dre04141, proteasome—dre03015) were defined by KEGG pathway IDs extracted from enriched pathways using KEGGREST^74^. All pathway enrichment analyses and heat-maps were clustered by Ward’s method using Euclidean distance.

### Functional characterization of mutant fish

Mutant lines were out-crossed to *ikzf1*-GFP transgenic fish, sorted for GFP expression at 24 h.p.f., and for carrier detection by genotyping fin clips at 3 weeks of age. Embryos from an in-cross of heterozygous carriers for the mutation and *ikzf1*-GFP transgene were sorted at 5 d.p.f. into fish with (+/+; +/−) and without (−/−) GFP expression in the thymus. These embryos were used in alcian blue staining, cell cycle analysis, TUNEL staining, and protein lysate preparation for Western blots.

### Alcian blue staining

Embryos were fixed with 4% w/v PFA and stored in 100% MeOH at −20 °C until use. Embryos were rehydrated in a decreasing gradient of MeOH to PBST and incubated in a solution containing H_2_O_2_ (10% v/v) and KOH (0.5% w/v) for 3-4 h at RT until bubbles disappeared. Embryos were washed with PBST and incubated in Alcian blue staining solution (0.01% w/v Alcian blue 8 GX [Sigma, Cat#A5268], 1% v/v HCl and 70% v/v EtOH) at RT overnight. Background staining was removed by incubation in a clearing solution (5% v/v HCl and 70% v/v EtOH) at RT overnight. Embryos were dehydrated in an increasing EtOH gradient and imaged in 80% v/v Glycerol.

### Cell cycle analysis

The yolk sac was removed from embryos by gentle aspiration in 0.5× Ginzburg Fish Ringer solution (55 mM NaCl, 1.8 mM KCl, 1.25 mM NaHCO_3_, without Ca^2+^). Embryos were digested in CO_2_-independent media (Life Technologies, Cat# 18045088) containing 3 mg/mL of collagenase II (Santa Cruz, Cat#sc-506177) at 37 °C under gentle rotation. The cell suspension was passed through a 70 μm cell strainer (BD, Cat#352350), washed with PBS and pelleted at 2800×*g*. Cells were stained for viability using ZombieRed Fixable Viability Kit (Biolegend, Cat# 423110) for 20 min at RT, protected from light. Cells were washed with FACS buffer (PBST, 1% BSA w/v), fixed and permeabilized with the FoxP3/TF Staining Buffer Set (eBioscience, Cat#00-5523-00) according to the manufacturer’s instructions. Cells were stained with 1 μg/mL Hoechst 33258 (Thermo Fisher Scientific, Cat# 33258) at 4 °C for 15 min and analysed using the BD Fortessa II and FACSDiva Software on a linear scale. Wild-type fish treated with 0.1 μg/mL nocodazole or 3 μM etoposide were used as G2/M and S phase inhibitor controls, respectively.

### Western blotting

Embryos were mechanically disrupted in 10 μL/embryo of lysis buffer (1× RIPA lysis buffer [Millipore, Cat#20-188], 0.1% SDS, 1 tablet/10 mL complete mini protease inhibitor cocktail [Roche, Cat#11836170001]). Lysates were centrifuged at 18,000 × *g* for 5 min and the resulting supernatants transferred to a fresh tube prior to snap freezing. Protein concentrations were determined using the BCA Protein Assay Kit (ThermoFisherScientific, Cat#23225). Protein lysates (10 μg) were incubated with 2× loading buffer (5× = 10% SDS w/w, 10 mM DTT, 20% Glycine, 0.05% bromophenol blue w/w, 0.2 M Tris, pH 6.8) at 95 °C for 10 min. Heat-denatured protein samples were separated in precast Mini-Protean TGX Gels (Biorad, Cat#456-8093) by SDS–PAGE and transferred to Immuno-Blot PVDF membranes (Biorad, Cat#1620177) in transfer buffer (25 mM Tris, 192 mM Glycine, 20% MeOH v/v) for 40 min at 100 V. Membranes were stained with amido black (Sigma, Cat#A8181-1EA) according to the manufacturer’s instructions to quantify protein loading. Membranes were blocked using 5% BSA and blotted for rabbit α-GRP78/HSP5 (1:2000, Cat#PA524963, Life Technologies), rabbit α-GADD153/CHOP (1:2000, Cat#G6916, Sigma-Aldrich), rabbit α-eIF2a (1:1000, Cat# PA5-41916, Thermo Fisher Scientific), and mouse α-vertebrate-β-actin (1:2000, Cat# A2066, Sigma-Aldrich) overnight at 4 °C. Membranes were probed with goat α-rabbit-HRP secondary (1:2000, Cat# P0448, Sigma-Aldrich), goat α-mouse-HRP secondary (1:2000, Cat# P0447, Sigma-Aldrich) with ECL Prime Western Blotting Detection Reagent (Amersham, Cat# RPN2232) and CX-BL + X-ray film (AGFA Healthcare) were used for detection. Contrast and brightness were globally adjusted using Adobe Photoshop CS6.

### TUNEL assay

For TUNEL staining, mutant embryos sorted from an in-cross of heterozygous carriers were dechorionated and fixed at 32 h.p.f. with 4% PFA w/v. Embryos were treated with 10 μg/mL of proteinase K for 40 min at RT and post-fixed with 4% PFA w/v at RT for 20 min. Embryos were permeabilized for 1 h with PBS containing Triton X-100 (0.1% v/v). TUNEL staining was performed using the DeadEnd Fluorometric TUNEL System (Promega, Cat# G3250) according to the manufacturer’s instructions. Stained embryos were imaged in 80% v/v Glycerol.

### Alternative splicing analysis

Detection of differential splicing events was performed using rMATS v.4.0.2 (ref. ^75^) on bam files downloaded from the Galaxy history. The following parameters were specified: read length (100 nucleotides), read type (paired), cutoff splicing difference (0.0001), analysis type (unpaired), library type (fr-firststrand). Mutant bam files were submitted as group 1 (-b1), and wild type as group 2 (-b2). Alternative splicing events supported by reads spanning splice junctions were reported as counts of significant events of alternative splicing (skipped and retained intron) relative to wild-type siblings (read covering exon boundary) (FDR ≤ 0.05, |inclusion level difference| ≥ 0.250).

### Ribosome riogenesis

Defects in ribosome biogenesis were determined by comparison of mutant 18S/28S rRNA ratios^76^ using BioAnalyzer. Log_2_ fold-changes of ribosomal RNA 18S/28S ratios were calculated relative to the values of corresponding wild-type siblings.

### Chemical inhibition

Inhibitors^32,36,77–88^ (Supplementary Table 5) were resuspended in DMSO or water at the manufacturers’ specified concentration and stored at −20 °C. Inhibitors were titrated and depicted at concentrations that showed no or minimal gross morphological defects (Fig. 3). IC30 values were determined using a three parameter log-logistic function. IC30 (equivalent to a fitness value of 0.7) were chosen for the analysis of genetic interactions, since the expected fitness of the inhibitor combinations corresponds to IC50 (0.7 × 0.7 fitness by the multiplicative method). A concentration eliciting a 50% reduction in *rag1*/*gh* ratio relative to untreated fish affords the best sensitivity for detecting positive and negative effects on T cell development. Zebrafish were grown in 35 mm dishes from 3 to 5 d.p.f. in 2.5 mL of E3 medium with or without inhibitor. RNA in situ hybridisation and *rag1*/*gh* ratios were compared between inhibitor-treated and control groups of an equivalent DMSO concentration at 5 d.p.f.; in all cases, DMSO concentrations were kept below 0.5% (v/v).

### Genetic interaction determination and nomenclature

T cell fitness values for single mutants (${W}_{x}$ and ${W}_{y}$) and double mutants (${W}_{{xy}}$) were calculated by normalizing mutant *rag1*/*gh* values to the corresponding wild-type *rag1*/*gh* values. Raw data for the genetic interaction network are listed in Supplementary Data 1. The multiplicative model was used to calculate expected fitness ($E({W}_{{xy}})$) as this model was the most accurate in predicting observed fitness as determined by the residual mean squared error^10^. Expected fitness values were also determined using other methods (Additive, Log and Minimum) for comparative purposes, without material differences in outcome.

Multiplicative

1 $$E({W}_{xy})={W}_{x}\times {W}_{y}$$

Additive

2 $$E({W}_{{xy}})={W}_{x}+(1-{W}_{y})$$

Log

3 $$E({W}_{{xy}})={\log}2\left[(2^{{W}_{x}}-1)\times (2^{{W}_{y}}-1)+1\right]$$

and minimum

4 $$E(W_{xy})=\min(W_{x},W_{y})$$

Additive propagated error for the expected fitness (${\epsilon }_{E\left({W}_{{xy}}\right)}$) was determined using standard deviations (${\delta }_{{W}_{x}}$ and ${\delta }_{{W}_{y}}$) and fitness values (${W}_{x}$ and ${W}_{y}$) of the single mutants in the following equation:

Propagated error

5 $${\epsilon }_{E\left({W}_{{xy}}\right)}=\sqrt{{\left(\frac{{\delta }_{{W}_{x}}}{{Wx}}\right)}^{2}+{\left(\frac{{\delta }_{{W}_{y}}}{{Wy}}\right)}^{2}}$$

The degree of genetic interaction was determined as the log_2_ fold-change between observed ${W}_{{xy}}$ and expected $E({W}_{{xy}})$ fitness values.

Genetic interactions were categorised into four types, non-interactive, negative, positive-co-equal and positive-suppressive. Non-interactive interactions were defined as an observed double-mutant fitness not significantly (*H*_0_) different from the expected fitness of the double-mutant.

Non-interactive

6 $$H_{0}{:}{W}_{{xy}}=E({W}_{{xy}})$$

Negative interactions were defined as an observed double-mutant fitness significantly (*H*_A_) less than the expected fitness of the double-mutant.

Negative interaction

7 $$H_{{{{{\mathrm{A}}}}}}{:}{W}_{{xy}} \, < \, E({W}_{{xy}})$$

Positive–coequal interaction is an observed double-mutant fitness significantly greater than the expected fitness of the double-mutant, but equivalent to the least fit single mutant.

Positive–coequal interaction

8 $${min}({W}_{x},{W}_{y})+{\delta }_{min (W_{x},W_{y})} \, > \, H_{A}{:}W_{xy} \, > \, E(W_{xy})$$

Positive–suppressive interaction is said to occur when an observed double-mutant fitness is significantly greater than the expected fitness for the double mutant and the least fit single mutant.

Positive–suppressive interaction

9 $${H}_{A}{:}W_{xy} \, > \, \min({W}_{x},{W}_{y})+{\delta }_{\min({W}_{x},{W}_{y})}$$

Genetic interactions were considered valid and retained in the network if, (i) the fitness values of both single mutants were less than that of the wildtype (${W}_{x}$ and ${W}_{y}$ < 1), and (ii) the type of genetic interaction as determined by the multiplicative method was concordant with the majority vote of the three other methods (additive, log and minimum).

*P* value estimates for between-pathway interactions were obtained by bootstrapping. Genetic interaction types (non-interactive, negative, and positive) were resampled from the proportion of genetic interaction types in the network (*P*_non-interactive_ = 0.47, *P*_negative_ = 0.299, *P*_positive_ = 0.231 [*P*_positive–coequal_ = 0.183_,_ *P*_positive-suppressive_ = 0.048]). The observed between-pathway proportion was then compared to the sampling distribution to determine a *P* value estimate.

### Transient leukaemia model

Wild-type zebrafish were injected with *SceI*-flanked *rag2*:*Myc*-GFP and *cmlc*:GFP vectors at the 1-cell stage^24^. The injection solution consisted of 1 μg/μL of combined plasmids, 0.5× CutSmart Buffer (New England BioLabs—NEB, Cat#B7204S), 0.3 U/μL I-SceI (NEB, Cat#R0694S) in Danieau buffer. Fish were sorted at 2 d.p.i. for GFP expression in the heart (*cmlc*), as this indicates successful incorporation of both transgenes in the majority of positive fish. Transgenic fish were sorted again at 5 weeks post-injection to remove those without *Myc*-GFP expression in the thymus. As the injected fish develop leukaemia at different time points (5–30 weeks of age), fish were partitioned appropriately to ensure an even split of leukaemia progression in each treatment group. Combinations of inhibitors (Supplementary Table 6) were initially titrated in 5-week old adolescent *lck*-CFP fish^41^. Combination treatments were compared to single inhibitor treatments carried out at the same concentrations to examine the presence of synthetic lethality. All drug solutions were kept to a consistent volume (50 μL) and compared to an equivalent quantity of DMSO solution (untreated). Fish were kept in tanks with 600 mL of water containing inhibitors, which was changed every two days over 3 weeks. Images were taken on days 0, 7, 14, and 21, using *Myc*-GFP expression to monitor tumour remission/progression. Leukaemia progression was quantified by measuring the GFP-positive pixel area relative to size of the fish using ImageJ software; the results were normalised to fluorescence observed at day 0. A *z* statistic was used for comparing regression slope coefficients (*b*) of tumour progression.

*z* statistic

10 $$z=\frac{{b}_{1}-{b}_{2}}{\sqrt{{{SE}{b}_{1}}^{2}+{{SE}{b}_{2}}^{2}}}$$

### Tissue-specific expression of genes

Expression of genes identified from the ENU forward genetic screen with their mouse orthologs was determined for each tissue from the genome wide BioGPS microarray datasets (GNF1M and MOE430, http://biogps.org/dataset/). Consisting of 93 tissue types and cell lines of mouse origin, the dataset was partitioned into several categories. The category *T cell* refers to CD4+ T cells, CD8+ T cells, FoxP3+ T cells, CD4+CD8+ DP thymocytes, CD4+ SP thymocytes, CD8+ SP thymocytes and thymus. The category *non-T immune cell* refers to B cells, follicular B cells, marginal zone B cells NK cells, granulocyte, dendritic cells, plasmacytoid DCs, macrophage, mast cells, GMP, CMP, MEP, HSC. The category *Immune* combines the categories *T cell* and *non-T immune cell*. The category *Non-immune* refers to adipose tissue, adrenal gland, brain, bladder, bone, eye, heart, liver, lung, mammary gland, ovary, pancreas, placenta, prostate, muscle, stomach, testis and uterus. Lymphoid organs containing a mixture of immune cells (spleen, bone marrow and lymph nodes), tissues with ancient immune function (kidney), T cell enriched tissues (intestines), and all cell lines were excluded from the analysis. Relative expression levels (log_2_) for each gene were determined between the mean expression levels for relevant categories. The tissue expression levels of genes identified in the ENU screens (*ENU* genes) were compared to those of known T cell genes (T cell receptor–KEGG pathway mmu04660), p53 genes (p53 signalling–KEGG pathway dre04115) and a random selection of genes from the genome (1000 replicates of the same numbers of genes that were identified in the ENU screens). Genes were defined as tissue-specific, if their expression was significantly greater than background tissue expression (*z* score ≥ 1.96). The proportions of tissue-specific genes were determined for genes identified in the ENU screen (*ENU* genes), T cell-related genes, p53-pathway components, and a random selection of genes from the genome after a normalization step considering the numbers of genes in different categories.

### Statistics and reproducibility

All data analysis and plotting was performed using R version 3.3.3 and R studio version 1.1.153 or GraphPad Prism 5. R scripts used for analysis are included in the GitHub repository (https://github.com/connoromeara/OMeara_et_al_2021). The numbers of biological replicates are indicated in the files associated with the NCBI Gene Expression Omnibus (GEO) (GSE147555) and NCBI Sequence Read Archive (SRA) (PRJNA622735) depositions, in Supplementary Data 1 and Supplementary Data 2 (comprising source files), and in the figure legends, where appropriate, as are the statistical tests used for the evaluation of experimental outcomes.

### Data repositories and image processing

Paired-end raw RNA-Seq reads from mutant lines, normalised counts and differential gene expression output from DESeq2 can be found at NCBI Gene Expression Omnibus (GEO) (GSE147555). Paired-end raw WGS-seq reads from mutant lines can be found in NCBI Sequence Read Archive (SRA) (PRJNA622735). Contrast, brightness and colour balance were globally adjusted for in situ, fluorescent and Western blot images using Adobe Photoshop CS6. Uncropped Western blots are provided as Supplementary Fig. 6.

### Reporting summary

Further information on research design is available in the Nature Research Reporting Summary linked to this article.

### Supplementary information

Supplementary Information

Description of Additional Supplementary Files

Supplementary Data 1

Supplementary Data 2

Reporting Summary

## Data Availability

R scripts, raw data and annotation files are included in the GitHub repository (https://github.com/connoromeara/OMeara_et_al_2021), are listed in Supplementary Data 1, and reported in additional source data files provided in Supplementary Data 2. Paired-end raw RNA-Seq reads from mutant lines, normalised counts and differential gene expression output from DESeq2 can be found at NCBI Gene Expression Omnibus (GEO) (GSE147555). Paired-end raw WGS-seq reads from mutant lines can be found in NCBI Sequence Read Archive (SRA) (PRJNA622735).

## References

[1] Typas A (2008). High-throughput, quantitative analyses of genetic interactions in *E. coli*. Nat. Methods.

[2] Costanzo, M. et al. A global genetic interaction network maps a wiring diagram of cellular function. *Science* **353**, 10.1126/science.aaf1420 (2016). 10.1126/science.aaf1420 PMC5661885 27708008

[3] Roguev A (2008). Conservation and rewiring of functional modules revealed by an epistasis map in fission yeast. Science.

[4] Horn T (2011). Mapping of signaling networks through synthetic genetic interaction analysis by RNAi. Nat. Methods.

[5] Horlbeck MA (2018). Mapping the genetic landscape of human cells. Cell.

[6] Najm FJ (2108). Orthologous CRISPR-Cas9 enzymes for combinatorial genetic screens. Nat. Biotechnol..

[7] Wong AS (2016). Multiplexed barcoded CRISPR-Cas9 screening enabled by CombiGEM. Proc. Natl. Acad. Sci. USA.

[8] Lehner B, Crombie C, Tischler J, Fortunato A, Fraser AG (2006). Systematic mapping of genetic interactions in *Caenorhabditis elegans* identifies common modifiers of diverse signaling pathways. Nat. Genet..

[9] Manguso RT (2017). In vivo CRISPR screening identifies Ptpn2 as a cancer immunotherapy target. Nature.

[10] Mani R, Onge RPS, Hartman JL, Giaever G, Roth FP (2008). Defining genetic interaction. Proc. Natl. Acad. Sci. USA.

[11] Lord CJ, Ashworth A (2017). PARP inhibitors: synthetic lethality in the clinic. Science.

[12] Huang A, Garraway LA, Ashworth A, Weber B (2020). Synthetic lethality as an engine for cancer drug target discovery. Nat. Rev. Drug Discov..

[13] Rabbitts TH (2009). Commonality but diversity in cancer gene fusions. Cell.

[14] Belver L, Ferrando A (2016). The genetics and mechanisms of T cell acute lymphoblastic leukaemia. Nat. Rev. Cancer.

[15] Degryse S (2018). Mutant JAK3 phosphoproteomic profiling predicts synergism between JAK3 inhibitors and MEK/BCL2 inhibitors for the treatment of T-cell acute lymphoblastic leukemia. Leukemia.

[16] Maude SL (2015). Efficacy of JAK/STAT pathway inhibition in murine xenograft models of early T-cell precursor (ETP) acute lymphoblastic leukemia. Blood.

[17] Peirs S (2014). ABT-199 mediated inhibition of BCL-2 as a novel therapeutic strategy in T-cell acute lymphoblastic leukemia. Blood.

[18] Boehm T, Bleul CC, Schorpp M (2003). Genetic dissection of thymus development in mouse and zebrafish. Immunol. Rev..

[19] Schorpp M (2006). Conserved functions of Ikaros in vertebrate lymphocyte development: genetic evidence for distinct larval and adult phases of T cell development and two lineages of B cells in zebrafish. J. Immunol..

[20] Iwanami N (2016). Forward genetic screens in zebrafish identify pre-mRNA-processing pathways regulating early T cell development. Cell Rep..

[21] Iwanami N (2011). Genetic evidence for an evolutionarily conserved role of IL-7 signaling in T cell development of zebrafish. J. Immunol..

[22] Iwanami, N. et al. Transgenerational inheritance of impaired larval T cell development in zebrafish. *Nat. Commun*. **11**, 10.1038/s41467-020-18289-9 (2020). 10.1038/s41467-020-18289-9 PMC7481223 32908148

[23] Iwanami N (2020). Epigenetic protection of vertebrate lymphoid progenitor cells by Dnmt1. iScience.

[24] Wienholds E, Schulte-Merker S, Walderich B, Plasterk RH (2002). Target-selected inactivation of the zebrafish rag1 gene. Science.

[25] Gudkov AV, Komarova EA (2003). The role of p53 in determining sensitivity to radiotherapy. Nat. Rev. Cancer.

[26] Paulsen RD (2009). A genome-wide siRNA screen reveals diverse cellular processes and pathways that mediate genome stability. Mol. Cell.

[27] Hess I, Boehm T (2012). Intra-vital imaging of thymopoiesis reveals dynamic lympho-epithelial interactions. Immunity.

[28] Bajoghli B (2009). Evolution of genetic networks underlying the emergence of thymopoiesis in vertebrates. Cell.

[29] Cretu C (2018). Structural basis of splicing modulation by antitumor macrolide compounds. Mol. Cell.

[30] Martin L (2014). Identification and characterization of small molecules that inhibit nonsense-mediated RNA decay and suppress nonsense p53 mutations. Cancer Res..

[31] Prescher JA, Bertozzi CR (2006). Chemical technologies for probing glycans. Cell.

[32] Rasmussen U, Broogger Christensen S, Sandberg F (1978). Thapsigargine and thapsigargicine, two new histamine liberators from *Thapsia garganica* L. Acta Pharm. Suec..

[33] Li R, Murray AW (1991). Feedback control of mitosis in budding yeast. Cell.

[34] Vinton PJ, Weinert T (2017). A slowed cell cycle stabilizes the budding yeast genome. Genetics.

[35] Manasanch EE, Orlowski RZ (2017). Proteasome inhibitors in cancer therapy. Nat. Rev. Clin. Oncol..

[36] Kotake Y (2007). Splicing factor SF3b as a target of the antitumor natural product pladienolide. Nat. Chem. Biol..

[37] Nutley BP (2005). Preclinical pharmacokinetics and metabolism of a novel prototype DNA-PK inhibitor NU7026. Br. J. Cancer.

[38] Willmore E (2004). A novel DNA-dependent protein kinase inhibitor, NU7026, potentiates the cytotoxicity of topoisomerase II poisons used in the treatment of leukemia. Blood.

[39] Mizui Y (2004). Pladienolides, new substances from culture of Streptomyces platensis Mer-11107. III. In vitro and in vivo antitumor activities. J. Antibiot..

[40] Andersen TB, Lopez CQ, Manczak T, Martinez K, Simonsen HT (2015). Thapsigargin–from Thapsia L. to mipsagargin. Molecules.

[41] Giorgetti OB (2021). Antigen receptor repertoires of one of the smallest known vertebrates. Sci. Adv..

[42] Gutierrez A (2011). Pten mediates Myc oncogene dependence in a conditional zebrafish model of T cell acute lymphoblastic leukemia. J. Exp. Med..

[43] Palomero T (2007). Mutational loss of PTEN induces resistance to NOTCH1 inhibition in T-cell leukemia. Nat. Med..

[44] Jagannathan-Bogdan M, Zon LI (2013). Hematopoiesis. Development.

[45] Papathanasiou P, Goodnow CC (2005). Connecting mammalian genome with phenome by ENU mouse mutagenesis: gene combinations specifying the immune system. Annu. Rev. Genet..

[46] Abeler-Dörner L (2020). High-throughput phenotyping reveals expansive genetic and structural underpinnings of immune variation. Nat. Immunol..

[47] Hu MG (2011). CDK6 kinase activity is required for thymocyte development. Blood.

[48] Malumbres M (2004). Mammalian cells cycle without the D-type cyclin-dependent kinases Cdk4 and Cdk6. Cell.

[49] Pagano M, Jackson PK (2004). Wagging the dogma; tissue-specific cell cycle control in the mouse embryo. Cell.

[50] Siamishi, I. et al. Lymphocyte-specific function of the DNA polymerase epsilon subunit POLE3 revealed by neomorphic alleles. *Cell Rep*. **31**, 10.1016/j.celrep.2020.107756 (2020). 10.1016/j.celrep.2020.107756 32553171

[51] White JK (2013). Genome-wide generation and systematic phenotyping of knockout mice reveals new roles for many genes. Cell.

[52] Li E, Bestor TH, Jaenisch R (1992). Targeted mutation of the DNA methyltransferase gene results in embryonic lethality. Cell.

[53] Pan R (2017). Synthetic lethality of combined Bcl-2 inhibition and p53 activation in AML: mechanisms and superior antileukemic efficacy. Cancer Cell.

[54] Gao S, Lai L (2018). Synthetic lethality in drug development: the dawn is coming. Future Med. Chem..

[55] Magen A (2019). Beyond synthetic lethality: charting the landscape of pairwise gene expression states associated with survival in cancer. Cell Rep..

[56] Possik PA (2014). Parallel in vivo and in vitro melanoma RNAi dropout screens reveal synthetic lethality between hypoxia and DNA damage response inhibition. Cell Rep..

[57] Langmead B, Salzberg SL (2012). Fast gapped-read alignment with Bowtie 2. Nat. Methods.

[58] Afgan E (2018). The Galaxy platform for accessible, reproducible and collaborative biomedical analyses: 2018 update. Nucl. Acids Res..

[59] Blankenberg D (2010). Manipulation of FASTQ data with Galaxy. Bioinformatics.

[60] Giardine B (2005). Galaxy: a platform for interactive large-scale genome analysis. Genome Res..

[61] Goecks J, Nekrutenko A, Taylor J (2010). Galaxy Team, Galaxy: a comprehensive approach for supporting accessible, reproducible, and transparent computational research in the life sciences. Genome Biol..

[62] Dale RK, Pedersen BS, Quinlan AR (2011). Pybedtools: a flexible Python library for manipulating genomic datasets and annotations. Bioinformatics.

[63] Quinlan AR, Hall IM (2010). BEDTools: a flexible suite of utilities for comparing genomic features. Bioinformatics.

[64] Obholzer N (2012). Rapid positional cloning of zebrafish mutations by linkage and homozygosity mapping using whole-genome sequencing. Development.

[65] Cingolani P (2012). A program for annotating and predicting the effects of single nucleotide polymorphisms, SnpEff: SNPs in the genome of *Drosophila melanogaster* strain w1118; iso-2; iso-3. Fly.

[66] Robinson JT (2011). Integrative genomics viewer. Nat. Biotechnol..

[67] Thorvaldsdottir H, Robinson JT, Mesirov JP (2013). Integrative Genomics Viewer (IGV): high-performance genomics data visualization and exploration. Brief Bioinform..

[68] Soza-Ried C, Hess I, Netuschil N, Schorpp M, Boehm T (2010). Essential role of c-myb in definitive hematopoiesis is evolutionarily conserved. Proc. Natl. Acad. Sci. USA.

[69] Herzog W (2003). Adenohypophysis formation in the zebrafish and its dependence on sonic hedgehog. Dev. Biol..

[70] Dobin A (2013). STAR: ultrafast universal RNA-seq aligner. Bioinformatics.

[71] Liao Y, Smyth GK, Shi W (2014). featureCounts: an efficient general purpose program for assigning sequence reads to genomic features. Bioinformatics.

[72] Love MI, Huber W, Anders S (2014). Moderated estimation of fold change and dispersion for RNA-seq data with DESeq2. Genome Biol..

[73] Yu G, Wang LG, Han Y, He QY (2012). clusterProfiler: an R package for comparing biological themes among gene clusters. OMICS.

[74] Tenenbau, D. KEGGREST: Client-side REST access to KEGG. R package version 1.16.1 (Bioconductor) (2017).

[75] Shen S (2014). rMATS: robust and flexible detection of differential alternative splicing from replicate RNA-Seq data. Proc. Natl. Acad. Sci. USA.

[76] Quarello P (2016). Ribosomal RNA analysis in the diagnosis of Diamond-Blackfan anaemia. Br. J. Haematol..

[77] Grieder A, Maurer R, Stahelin H (1974). Effect of an epipodophyllotoxin derivative (VP 16-213) on macromolecular synthesis and mitosis in mastocytoma cells in vitro. Cancer Res..

[78] Di Marco A, Gaetani M, Scarpinato B (1969). Adriamycin (NSC-123,127): a new antibiotic with antitumor activity. Cancer Chemother. Rep..

[79] Wallace RE, Murdock KC, Angier RB, Durr FE (1979). Activity of a novel anthracenedione, 1,4-dihydroxy-5,8-bis(((2-[(2-hydroxyethyl)amino]ethyl)amino])-9,10-anthracenedione dihydrochloride, against experimental tumors in mice. Cancer Res..

[80] Handschumacher RE, Welch AD (1956). Microbial studies of 6-azauracil, an antagonist of uracil. Cancer Res..

[81] De Brabander MJ, Van de Veire RM, Aerts FE, Borgers M, Janssen PA (1976). The effects of methyl (5-(2-thienylcarbonyl)-1H-benzimidazol-2-yl) carbamate, (R 17934; NSC 238159), a new synthetic antitumoral drug interfering with microtubules, on mammalian cells cultured in vitro. Cancer Res..

[82] Huertas D (2012). Antitumor activity of a small-molecule inhibitor of the histone kinase Haspin. Oncogene.

[83] Yoon SO, Shin S, Lee HJ, Chun HK, Chung AS (2006). Isoginkgetin inhibits tumor cell invasion by regulating phosphatidylinositol 3-kinase/Akt-dependent matrix metalloproteinase-9 expression. Mol. Cancer Ther..

[84] Martin L (2014). Identification and characterization of small molecules that inhibit nonsense-mediated RNA decay and suppress nonsense p53 mutations. Cancer Res..

[85] Schneider EG, Nguyen HT, Lennarz WJ (1978). The effect of tunicamycin, an inhibitor of protein glycosylation, on embryonic development in the sea urchin. J. Biol. Chem..

[86] Fiebiger E (2004). Dissection of the dislocation pathway for type I membrane proteins with a new small molecule inhibitor, eeyarestatin. Mol. Biol. Cell.

[87] Perkel VS, Miura Y, Magner JA (1989). Brefeldin A inhibits oligosaccharide processing of glycoproteins in mouse hypothyroid pituitary tissue at several subcellular sites. Proc. Soc. Exp. Biol. Med..

[88] Sato M (2014). High antitumor activity of pladienolide B and its derivative in gastric cancer. Cancer Sci..

[89] Lawir DF, Iwanami N, Schorpp M, Boehm T (2017). A missense mutation in zbtb17 blocks the earliest steps of T cell differentiation in zebrafish. Sci. Rep..

[90] Mönnich M (2010). Developing T lymphocytes are uniquely sensitive to a lack of topoisomerase III alpha. Eur. J. Immunol..

[91] Lawir DF, Sikora K, O’Meara CP, Schorpp M, Boehm T (2020). Pervasive changes of mRNA splicing in upf1-deficient zebrafish identify rpl10a as a regulator of T cell development. Proc. Natl Acad. Sci. USA.

[92] den Dunnen JT, Antonarakis SE (2000). Mutation nomenclature extensions and suggestions to describe complex mutations: a discussion. Hum. Mutat..

