# Author Correction: Genetic landscape of T cells identifies synthetic lethality for T-ALL

**DOI:** 10.1038/s42003-024-05841-2

**Published:** 2024-02-14

**Authors:** Connor P. O’Meara, Lucia Guerri, Divine-Fondzenyuy Lawir, Fernando Mateos, Mary Iconomou, Norimasa Iwanami, Cristian Soza-Ried, Katarzyna Sikora, Iliana Siamishi, Orlando Giorgetti, Sarah Peter, Michael Schorpp, Thomas Boehm

**Affiliations:** 1 Department of Developmental Immunology, Max Planck Institute of Immunobiology and Epigenetics, 79108 Freiburg, Germany; 2 Bioinformatics Unit, Max Planck Institute of Immunobiology and Epigenetics, 79108 Freiburg, Germany; 3 Faculty of Medicine, University of Freiburg, Freiburg, Germany; 4 Present Address: Laboratory of Neurogenetics, National Institute of Alcohol Abuse and Alcoholism (NIAAA), National Institutes of Health (NIH), Bethesda, MD USA; 5 Present Address: Institute of Zoology, Developmental Biology Unit, University of Cologne, Cologne, Germany; 6 Present Address: Center for Bioscience Research and Education, Utsunomiya University, Utsunomiya, Japan; 7 Present Address: Fundacion Oncoloop & Center for Nuclear Medicine, Santiago, Chile; 8 Present Address: Luxembourg Centre for Systems Biomedicine, University of Luxembourg, Esch-sur-Alzette, Luxembourg

**Keywords:** Genetics, Immunology

Correction to: *Communications Biology* (2021) **4**:1201; 10.1038/s42003-021-02694-x, published online 20 October 2021.

The original version of the Supplementary information associated with this Article contained errors in the oligonucleotide sequences of the morpholinos ZF_PI4KAA_ATG and ZF_POLE_ATG in Supplementary Table 1. There were also sequencing errors in the forward and reverse sequences of primers for *mat2aa, nol9* and *eif5* in Supplementary Table 4; the tables should have appeared as shown below. The original file has been corrected.

**Supplementary Table 1 | Antisense morpholino oligonucleotides used in this study**.

**Table:** 

Mutant	Affected Gene	ENSEMBL Gene ID^a^	Name^b^	Sequence	Reference
JM087	*anapc1*	ENSDARG00000075687	ZF_ANAPC1_ATG	TGTACCACTTGACCAGCACTTTCAT	This study
JM087	*anapc1*	ENSDARG00000075687	ZF_ANAPC1_ACC5	CTTCATAGACGTGCGACATGAGTTC	This study
HU319	*atad5a*	ENSDARG00000070568	ZF_ATAD5A_ATG	GGCAATGCAACAACCCCAGCCATCT	This study
HU319	*atad5a*	ENSDARG00000070568	ZF_ATAD5A_DON3	AGAAGTGTTGATCTTACCTGATAGG	This study
JM052	*fcf1*	ENSDARG00000102333	ZF_FCF1_DON2	AAGACTCAAACTTACATTTCTTGTT	This study
JM052	*fcf1*	ENSDARG00000102333	ZF_FCF1_ATG	ATGACGGCTGAATTTGTTCGAGATT	This study
JZ061	*fli1a*	ENSDARG00000054632	ZF_FLI1A_ATG	CGCCTCCTTAATAGTTCCGTCCATT	This study
JZ061	*fli1a*	ENSDARG00000054632	ZF_FLI1A_DON7	TTGGAGAGCCTGAGAAATGGAAAGA	This study
KL069	*gemin5*	ENSDARG00000079257	GEMIN5ATG	GATGTCTTTCGTGCATTATATACCG	20
KL069	*gemin5*	ENSDARG00000079257	ZF GEMIN5 DON4	GCACAAAACCTCTAGTTTACCTGCA	20
KL069	*gemin5*	ENSDARG00000079257	ZF GEMIN5 ACC9	ATGCCAACTGTAAGAAAAGTGTGGA	20
18_10	*lsm8*	ENSDARG00000091656	ZF_LSM8 ACC4	CGATCACAGCCCTTAAACACAAAAT	20
18_10	*lsm8*	ENSDARG00000091656	ZF_LSM8 ACC3	CGTCCCCTAAAAACAGCACAAGTCA	20
HY062	*mat2aa*	ENSDARG00000040334	ZF_MAT2AA_ATG	AGCCGTTCAGTTGTCCGTTCATATT	This study
HY062	*mat2aa*	ENSDARG00000040334	ZF_MAT2AA_ACC4	ACCCTTAAAGTACAACACAGGGATT	This study
IG335	*mcm10*	ENSDARG00000045815	ZF_MCM10 ACC4	TCTGAAGAGGCTGATTTACATAAGA	This study
JI073	*naa50*	ENSDARG00000027825	ZF_NAA50_ACC2	CCGGCTACTAGAACAAAAGCAGAAT	This study
JI073	*naa50*	ENSDARG00000027825	ZF_NAA50_DON2	AGCGTTGTTACATACCTAGCTTGGC	This study
IT429	*nek7*	ENSDARG00000056966	ZF_NEK7_DON8	GATGGGTTTCTATACCTTACCTCAT	This study
IT429	*nek7*	ENSDARG00000056966	ZF_NEK7_ATG	CGTCCATTGTGACAGCAGCAGTCGC	This study
HP327	*nol9*	ENSDARG00000077751	ZF_NOL9 ACC4	AGCACTATATTTACCGAGTTGAGGC	This study
HP327	*nol9*	ENSDARG00000077751	ZF_NOL9 ATG (3RD_1)	GCTGACCCCCAACGAGACTATAAAC	This study
HG002	*pi4kaa*	ENSDARG00000076724	ZF_PI4KAA_ACC22	AGCTCAGCCTGGAAACAGCAAATGT	This study
HG002	*pi4kaa*	ENSDARG00000076724	ZF_PI4KAA_ATG	ACGTCCCTCTCGACGACATTATTCA	This study
IG447	*pip5k1ba*	ENSDARG00000044295	ZF_PIP5K1BA_DON5	TTGTGGATTGTGTGGCTCACCATGT	This study
IG447	*pip5k1ba*	ENSDARG00000044295	ZF_PIP5K1BA_ATG	GCTCATCTGCCGTTGCACTCATCTT	This study
JI065	*pnrc1*	ENSDARG00000043904	ZF_PNRC1_ACC2	GGCTGCTTTAGACAAACATGAAACA	This study
JI065	*pnrc1*	ENSDARG00000043904	ZF_PNRC1_ATG	GACGACCAAAAGCATCGCCCAACAT	This study
HG010	*pole1*	ENSDARG00000058532	ZF_POLE_ATG	GTCTGAAGACTTTCAAATCAGTTAC	20
HG010	*pole1*	ENSDARG00000058533	ZF_POLE_ACC17	ATCACACACCTGAAACAGGAAAAAT	20
HG010	*pole1*	ENSDARG00000058533	ZF_POLE_DON13	GATGAAAATTAGACCTGTGGTTCT	20
KW059	*snapc3*	ENSDARG00000101474	ZF_SNAPC3_ATG	TCTTTGCGTATCTCCGCCATAATTC	20
KW059	*snapc3*	ENSDARG00000101474	ZF_SNAPC3_ACC7	ATTACCCTTCAGCAAGAACACATAT	20
KH025	*eif5*	ENSDARG00000003681	ZF_EIF5_DON4	AATTTAATACTCACATGTCGGAGGC	This study
IG438	*spata5*	ENSDARG00000104869	ZF_SPATA5_ACC16	GACCACCAATATCACTCCACTTCAC	This study
IG438	*spata5*	ENSDARG00000104869	ZF_SPATA5_ATG	CTTTTCTTACTGGATGACATGATGC	This study
HI020	*tbcb*	ENSDARG00000068404	ZF_TBCB_ATG	GATTGTCACACTCCCGTCCATCTTC	This study
HI020	*tbcb*	ENSDARG00000068404	ZF_TBCB_ACC6	GCCGTACCTGAAAACAATAGAAGCA	This study
HA343	*tnpo3*	ENSDARG00000045680	TNPO3 ATG	GGTTTCCCGCCTTCCATGGTGCTCT	20
HA343	*tnpo3*	ENSDARG00000045680	TNPO3 SPLICE ACC6	TCATCCCTCTGCTTCAATGACGAGT	20
IM087	*ube3d*	ENSDARG00000026178	ZF_UBE3D_ACC9	CAACACTACACATCAGGGAAAAACA	This study
IM087	*ube3d*	ENSDARG00000026178	ZF_UBE3D_ATG	TCGCAGTCTCTTCCATTGGTATTTC	This study
IL015	*unc45a*	ENSDARG00000103643	ZF_UNC45A_ACC2/ATG	CTGGGACATCTACACAGTCAGAAAA	This study
HJ028	*upf1*	ENSDARG00000016302	ZF_UPF1_ACC2	GTTCACCTGAAAACAAGATGAGCAA	91
JZ007	*yeats2*	ENSDARG00000078767	ZF_YEATS2_DON25	AGAAACTGGCACACACTTACCTGGT	This study
JZ007	*yeats2*	ENSDARG00000078767	ZF_YEATS2_ACC23	CCGTGCTGAGGGAGATTGATAATAA	This study

^a^ Zv10

^b^ Morpholinos target translation initiation codon (ATG) or splice sites (*DON* donor, *ACC* acceptor, numbers refer to exons)

**Supplementary Table 4 | Genotyping primers used in this study**.

**Table:** 

Affected Gene	Primer	Type
*fli1a*	ATTTCTCAGGCTCTCCAACAG	Forward
	TAGCAAGTCGACTGCTGGTG	Reverse
*pole*	GTCTGTGGACATTTGATGCTTG	Forward
	GACTCCAGCTTGGACCCAC	Reverse
*tbcb*	ATGAGGAAGAGAGGGCCAAG	Forward
	CCACTGCTGTTAGGTACATC	Reverse
*unc45a*	GGGAGCCAAATAGTATTCAAG	Forward
	GCGGTACAGGACTGCACTCT	Reverse
*pnrc1*	CATAGACAAGACATCACCTG	Forward
	TGCTTCAGGATGTTTTCTGG	Reverse
*ube3d*	TGGATGTGTGGGAGAAGGAC	Forward
	TCAGAGTGGTGTGTGACCTG	Reverse
*naa50*	TGCACTGCTGGTTTACGGTG	Mutant
	TTAGGCTCTGTGTTGCATGTG	Mutant
	GTCAGTTCACAGCTAGTTGAC	Wildtype
	GTTGAGTTACGGCTTTGTTGTG	Wildtype
*yeats2*	GTCAAAGTAGAACAGGGC	Forward
	ATTCCCTCTGATTGTCCC	Reverse
*atad5a*	GACAGGCTCTTCAGTGTTGTC	Forward
	CAGCTTCAAGAGCAAGTCCTG	Reverse
*anapc1*	CAGCAGGGCGACTCATTTTG	Forward
	CTGAACTGGGCTGTCGACTG	Reverse
*nek7*	CAATTGAACCACCCCAATGT	Forward
	AATGGGCATGTGTCCTTACC	Reverse
*spata5*	GTCCGCAGGGTCCAGAGTTAC	Forward
	TGACGGAGCAACAGTTCTGG	Reverse
*mat2aa*	CCCAACTAACCAAGCCAAGTT	Forward
	AGTCTCGCTAGTGGCATAAC	Reverse
*nol9*	CCAACAGTGTTCTTCAGAACG	Forward
	ATGTGGATTGGACCTGGAAAC	Reverse
*eif5*	GCTCTAAATAGGCCTCCGACA	Forward
	CAGTGCATCAAGGGTACACAG	Reverse
*pi4kaa*	AAGGTGGAGTGTTGCTTTAAG	Forward
	CGTGACAGTGTCGTTCTTCAG	Reverse
*pip5k1ba*	ACTGAAACACAATCAAGCAAGTG	Forward
	CTGTTGCTAAAGACATGTTGTG	Reverse

### Supplementary information

Supplementary_Table_1_CORRECTED

Supplementary_Table_4_CORRECTED

