# Supplementary material for: Genetic landscape of T cells identifies synthetic lethality for T-ALL
Source: Commun Biol. 2021 Oct 20;4:1201. doi: 10.1038/s42003-021-02694-x (PMC8528931; doi:10.1038/s42003-021-02694-x)
Supplement: Supplementary file 1 — Supplementary Information [file 42003_2021_2694_MOESM1_ESM.pdf]

Supplementary Information

for

Genetic landscape of T cells identifies synthetic lethality for T-ALL

Connor P. O'Meara, Lucia Guerri, Divine-Fondzenyuy Lawir, Fernando Mateos, Mary Iconomou, Norimasa Iwanami, Cristian Soza-Ried, Katarzyna Sikora, Iliana Siamishi, Orlando Giorgetti, Sarah Peter, Michael Schorpp, and Thomas Boehm

Supplementary Figures 1-6

Supplementary Tables 1-6

Provided separately:

Supplementary Data 1

Supplementary Data 2 (Source Data files for Figures 5b, 9b, 9d and Supplementary Figures 1, 9b, 9d)

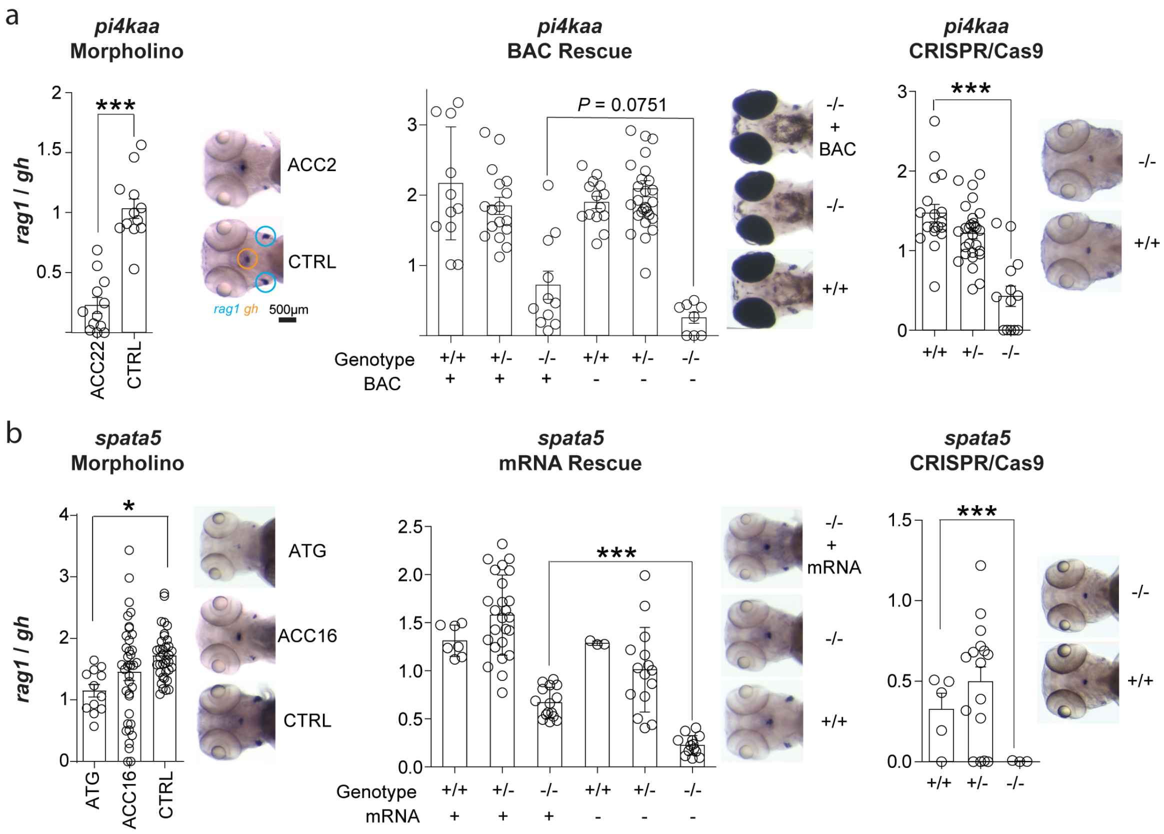
a
pi4kaa
Morpholino
rag1 / gh
***
ACC22
CTRL
ACC2
CTRL
rag1 gh
500µm
pi4kaa
BAC Rescue
P = 0.0751
Genotype +/+ +/- -/- +/+ +/- -/-
BAC + + + - - -
-/- + BAC
-/-
+/+
pi4kaa
CRISPR/Cas9
***
+/+ +/- -/-
-/-
+/+
b
spata5
Morpholino
rag1 / gh
*
ATG
ACC16
CTRL
spata5
mRNA Rescue
***
Genotype +/+ +/- -/- +/+ +/- -/-
mRNA + + + - - -
-/- + mRNA
-/-
+/+
spata5
CRISPR/Cas9
***
+/+ +/- -/-
-/-
+/+

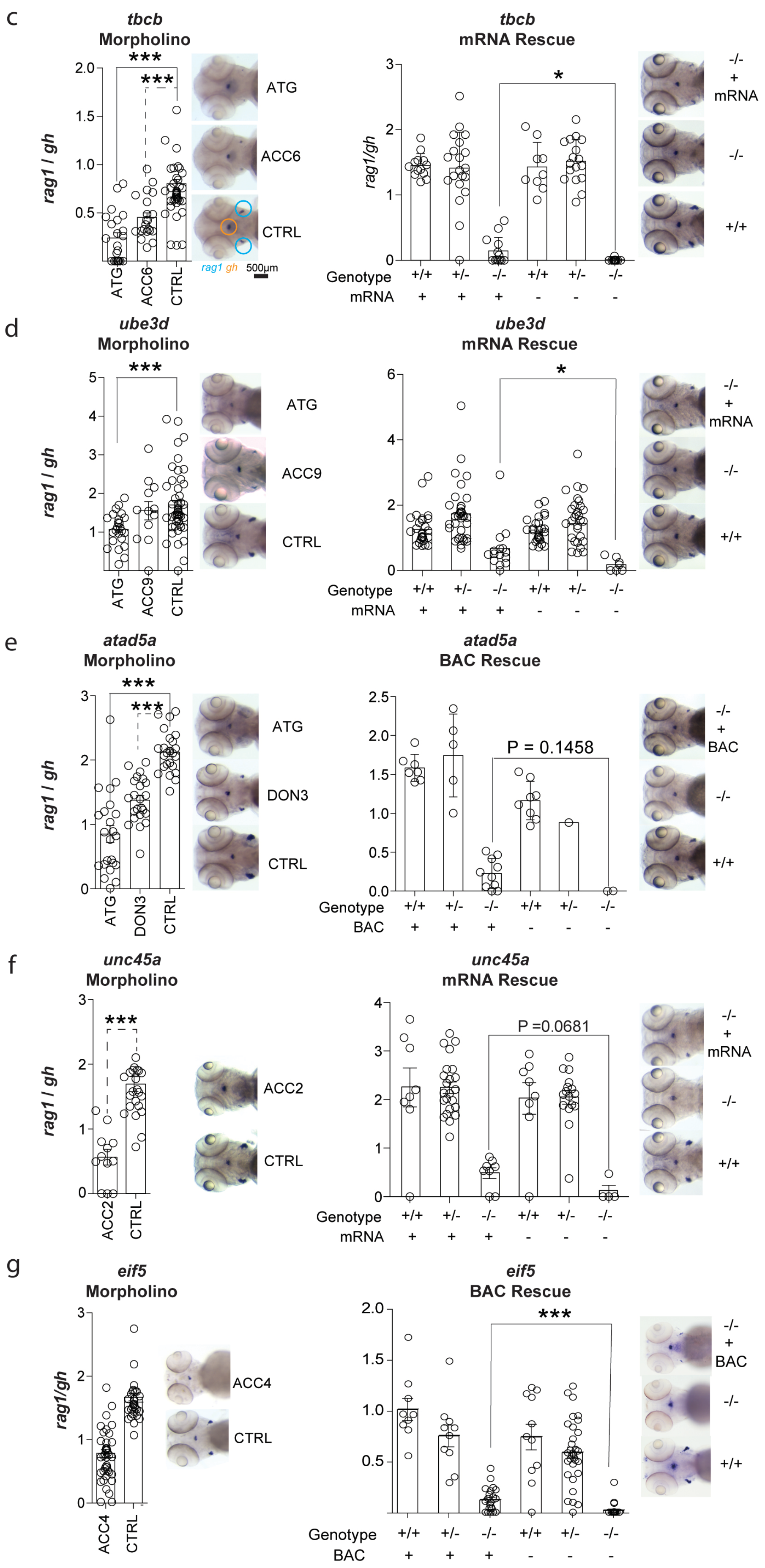

c
tbcb
Morpholino
***
***
rag1 / gh
2.0
1.5
1.0
0.5
ATG
ACC6
CTRL
rag1 gh
500µm
tbcb
mRNA Rescue
*
rag1/gh
3
2
1
0
Genotype +/+ +/- -/- +/+ +/- -/-
mRNA + + + - - -
-/-
+
mRNA
-/-
+/+
d
ube3d
Morpholino
***
rag1 / gh
5
4
3
2
1
ATG
ACC9
CTRL
ube3d
mRNA Rescue
*
6
4
2
0
Genotype +/+ +/- -/- +/+ +/- -/-
mRNA + + + - - -
-/-
+
mRNA
-/-
+/+
e
atad5a
Morpholino
***
***
rag1 / gh
3
2
1
0
ATG
DON3
CTRL
atad5a
BAC Rescue
P = 0.1458
2.5
2.0
1.5
1.0
0.5
0.0
Genotype +/+ +/- -/- +/+ +/- -/-
BAC + + + - - -
-/-
+
BAC
-/-
+/+
f
unc45a
Morpholino
***
rag1 / gh
3
2
1
0
ACC2
CTRL
unc45a
mRNA Rescue
P =0.0681
4
3
2
1
0
Genotype +/+ +/- -/- +/+ +/- -/-
mRNA + + + - - -
-/-
+
mRNA
-/-
+/+
g
eif5
Morpholino
rag1/gh
3
2
1
0
ACC4
CTRL
eif5
BAC Rescue
***
2.0
1.5
1.0
0.5
Genotype +/+ +/- -/- +/+ +/- -/-
BAC + + + - - -
-/-
+
BAC
-/-
+/+

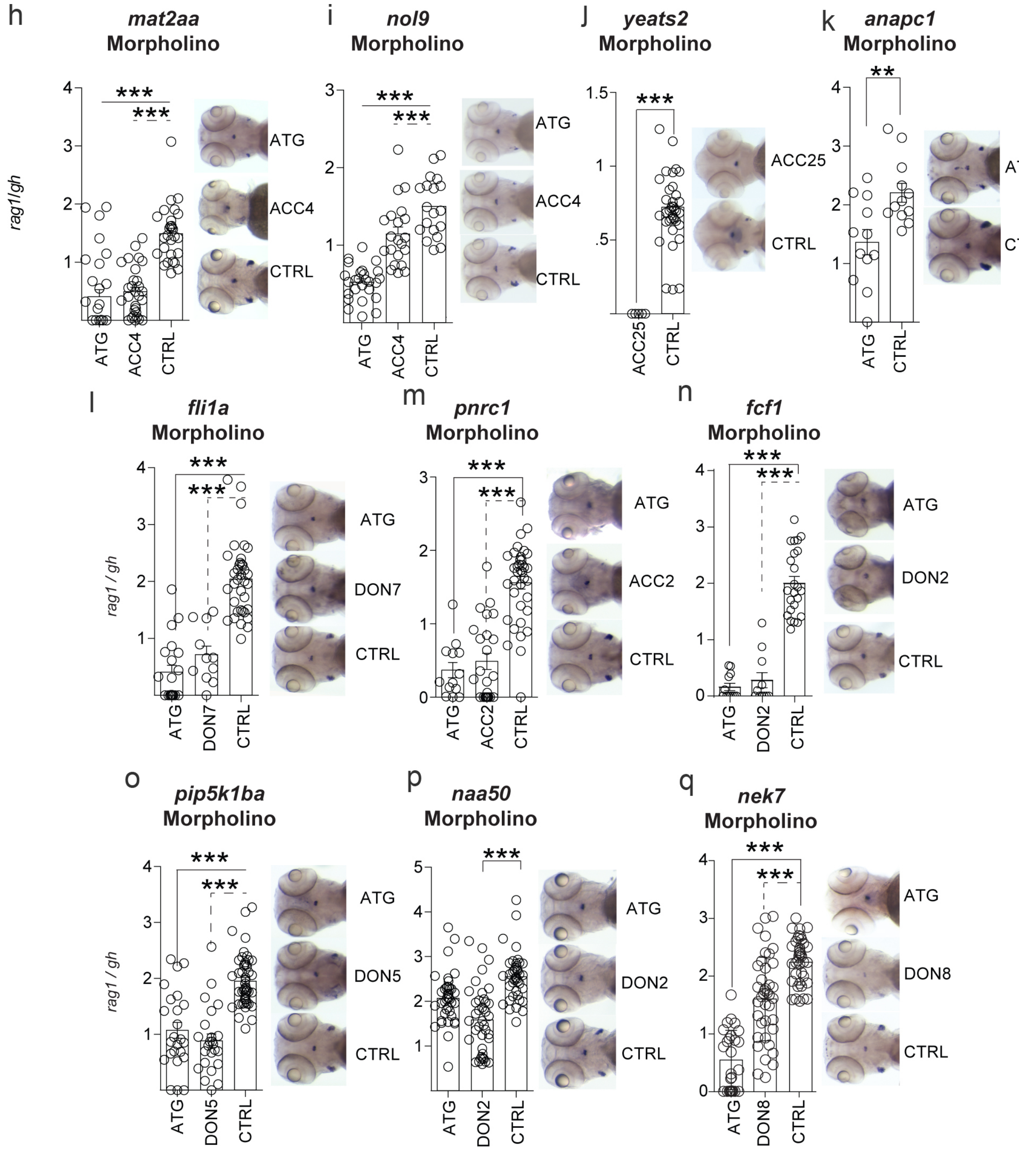

Supplementary Figure 1 | Validation of candidate genes. The roles of candidate genes in regulating T cell development were validated using morpholino-induced knock-down, mRNA/BAC-mediated rescue of phenotypes, and/or CRISPR/Cas9-mediated confirmatory gene disruption. **a**, *pi4kaa*. **b**, *spata5*. **c**, *tbcb*. **d**, *ubed3*. **e**, *atad5a*. **f**, *unc45a*. **g**, *eif5*. **h**, *mat2aa*. **i**, *nol9*. **j**, *yeats2*. **k**, *anapc1*. **l**, *fli1a*. **m**, *pnrc1*. **n**, *fcf1*. **o**, *pip5k1ba*. **p**, *naa50*. **q**, *nek7*.

The *rag1/gh* values for the individual genotypes/conditions are given; each data point represents one embryo. Morpholinos targeting initiation codons (ATG) or splice sites (donor splice site, DON; acceptor splice site, ACC; relevant exons are indicated) were injected into wild-type embryos and the *rag1/gh* ratios were compared to an un-injected control (CTRL) using a one-way ANOVA with a Dunnett's post-test. For mRNA/BAC-mediated rescue, fish from an in-cross of heterozygous parental fish were injected with wild-type mRNA of the

gene candidate or bacterial artificial chromosome (BAC) spanning the locus of the gene candidate. The presence of a rescue of the mutant phenotype was determined by comparison to un-injected mutant fish using two-tailed Student's *t*-test. Guide RNAs targeting the candidate genes were injected as RNPs in complex with Cas9 protein into wild-type fish. The resulting crispants were outcrossed to establish stable fish lines and selected for those that exhibited deleterious frame-shift mutations; the T cell phenotypes of fish homozygous for the induced genetic defects were compared to their genotypically wild-type siblings using two-tailed Student's *t*-test. Representative images approximating the mean values from each experiment are depicted. *, $P < 0.05$; **, $P < 0.01$; ***, $P < 0.001$. See Source Data for Supplementary Figure 1.

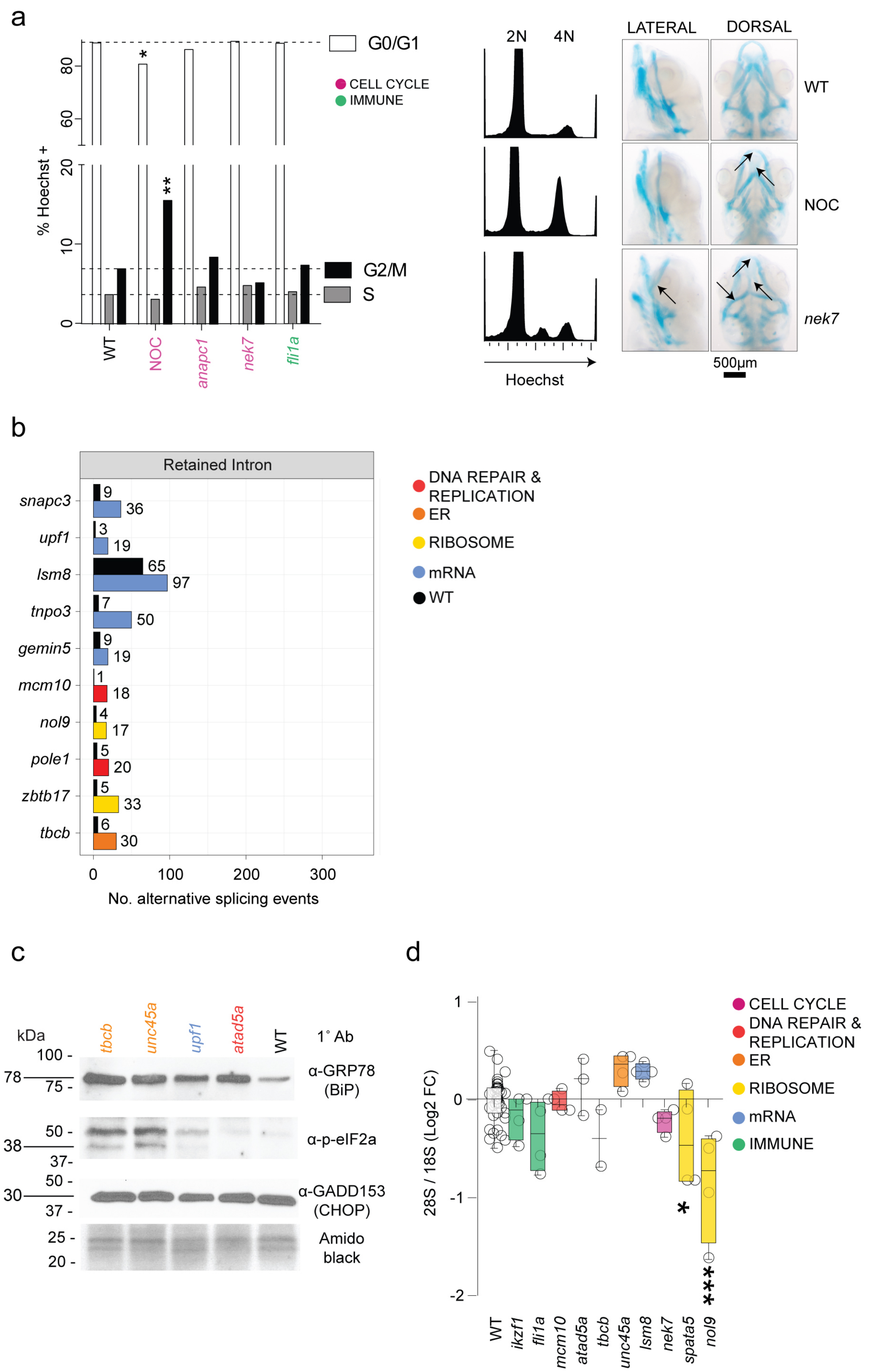
a
G0/G1
CELL CYCLE
IMMUNE
G2/M
S
% Hoechst +
0
10
20
60
80
*
**
WT
NOC
anapc1
nek7
fli1a
2N
4N
Hoechst
LATERAL
DORSAL
WT
NOC
nek7
500µm
b
Retained Intron
snapc3
upf1
lsm8
tnpo3
gemin5
mcm10
nol9
pole1
zbtb17
tbcb
9
36
3
19
65
97
7
50
9
19
1
18
4
17
5
20
5
33
6
30
0
100
200
300
No. alternative splicing events
DNA REPAIR &
REPLICATION
ER
RIBOSOME
mRNA
WT
c
kDa
tbcb
unc45a
upf1
atad5a
WT
1° Ab
100 -
78
75 -
α-GRP78
(BiP)
50 -
38
37-
α-p-eIF2a
50 -
30
37 -
α-GADD153
(CHOP)
25 -
20 -
Amido
black
d
28S / 18S (Log2 FC)
1
0
-1
-2
*
***
WT
ikzf1
fli1a
mcm10
atad5a
tbcb
unc45a
lsm8
nek7
spata5
nol9
CELL CYCLE
DNA REPAIR &
REPLICATION
ER
RIBOSOME
mRNA
IMMUNE

Supplementary Figure 2 | Validation of defects in biological functions. a, Hoechst stain-based cell cycle analysis of bulk cell suspensions from 5 d.p.f. zebrafish mutants (for each conditions at least 50 embryos were pooled and analysed together), indicating the proportions of cells in G0/G1, S and G2/M phases (left panels). Nocodazole (NOC)-treated wild-type fish were included as an example of G2/M phase inhibition. Representative flow cytometry plots of normal cell cycle (wild-type [WT]; top), S phase block (NOC; middle) and G2/M block (*nek*; bottom) are shown (middle panels). Significance was determined by Chi-square analysis; *, P <0.05; **, P <0.01. Alcian blue staining of cartilage in mutants with cell cycle defects reveals malformation of rapidly dividing cartilage cells (arrows), indicative of abnormal neural crest development (right panels). **b**, Numbers of significant events (depicted atop each bar; FDR ≤ 0.05, |Inclusion Level Difference| ≥ 0.250) showing alternative splicing (skipped and retained intron as determined by reads covering exon boundaries) of pre-mRNA in mutants relative to wild-type siblings. **c**, Western blot analysis of mutant zebrafish lysates resolved for ER stress-related components with α-GRP78, α-CHOP and α-P-eIF2 antibodies. Amido black staining of total protein was used as loading control. Uncropped versions of the Western blost are shown in Supplementary Figure 6. Size markers are indicated in kDa. **d**, 28S/18S rRNA ratios (log_2 changes) for genetic variants compared to wild-type siblings as a measure of ribosome biogenesis defects. Significance was determined by one-way ANOVA with Dunnett's post-test. *, P <0.05; **, P <0.01; ***, P <0.001. See Source Data for Supplementary Figure 2.

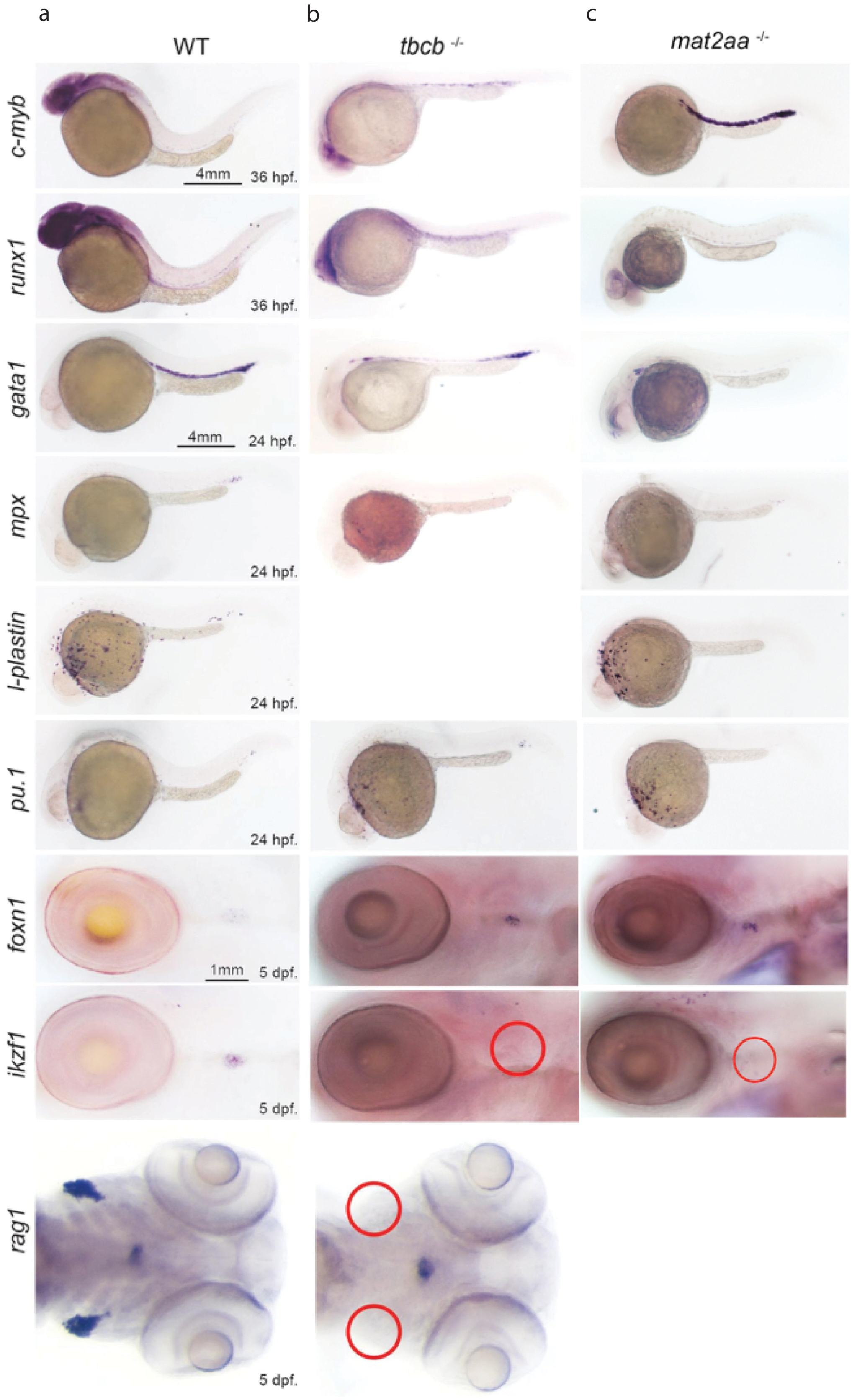

a
b
c
WT
tbcb -/-
mat2aa -/-
c-myb
runx1
gata1
mpx
l-plastin
pu.1
foxn1
ikzf1
rag1
4mm
36 hpf.
36 hpf.
4mm
24 hpf.
24 hpf.
24 hpf.
24 hpf.
1mm
5 dpf.
5 dpf.
5 dpf.

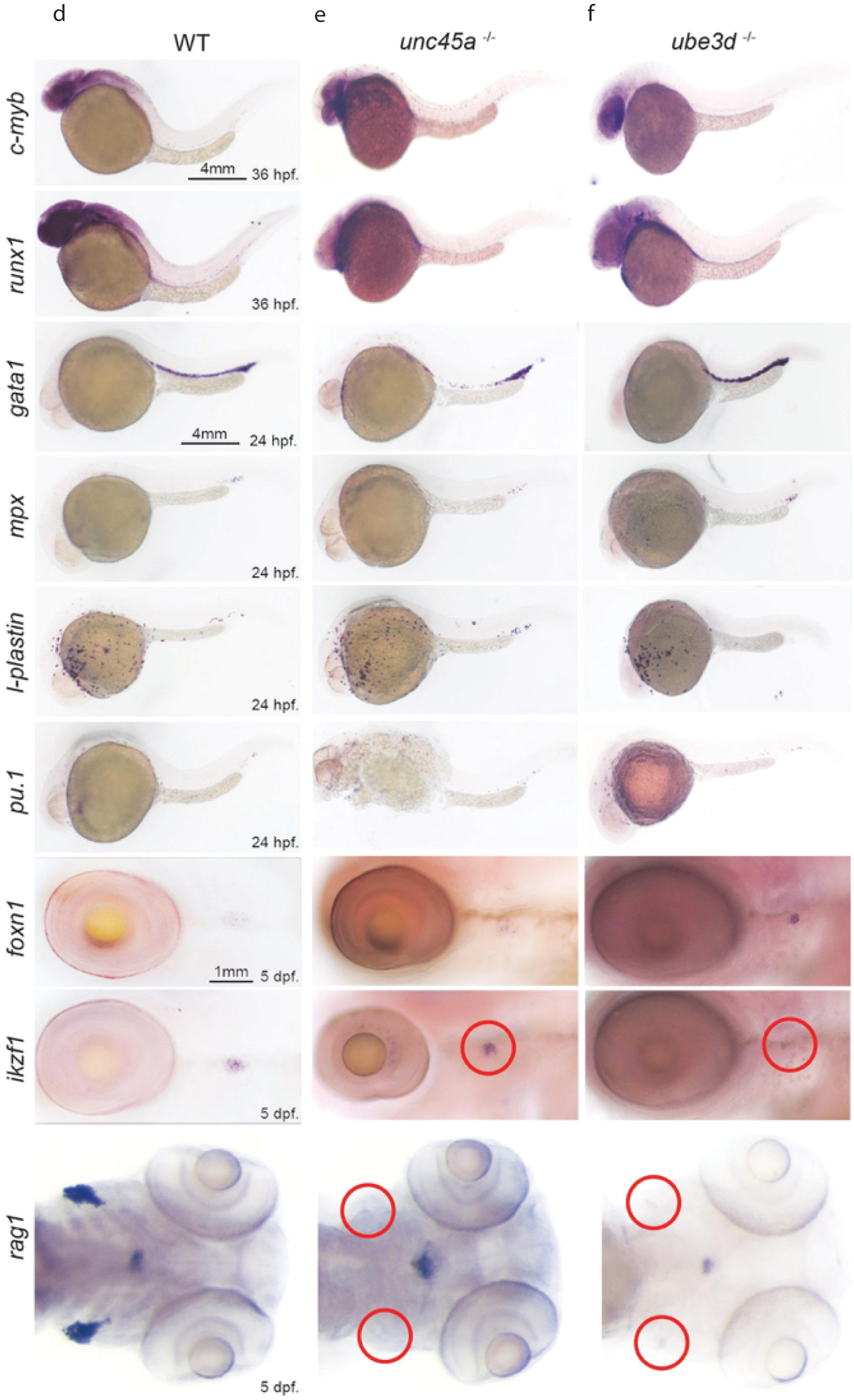
d
e
f
WT
unc45a -/-
ube3d -/-
c-myb
runx1
gata1
mpx
l-plastin
pu.1
foxn1
ikzf1
rag1
4mm
36 hpf.
36 hpf.
4mm
24 hpf.
24 hpf.
24 hpf.
24 hpf.
1mm
5 dpf.
5 dpf.
5 dpf.

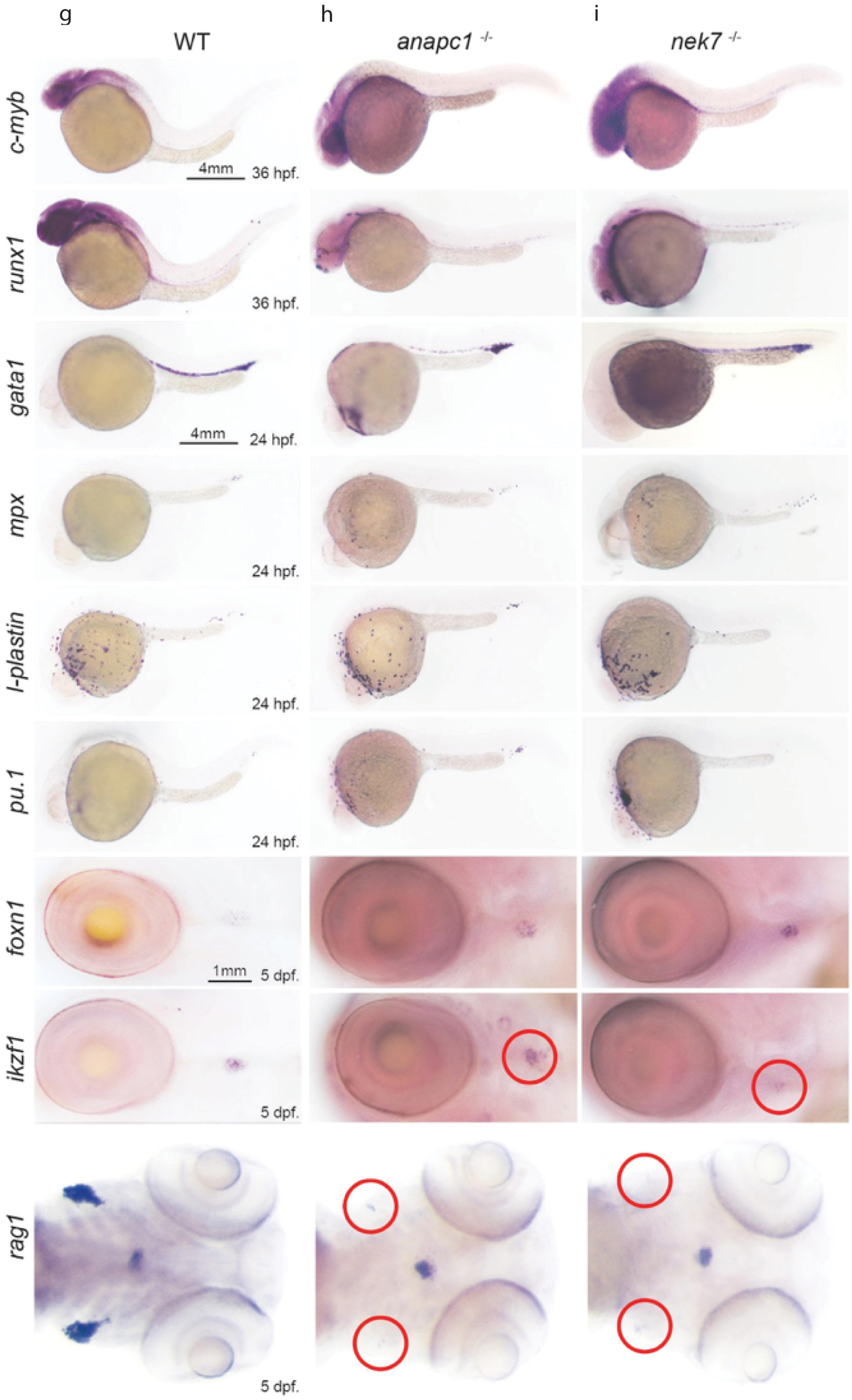
g
h
i
WT
anapc1 -/-
nek7 -/-
c-myb
runx1
gata1
mpx
l-plastin
pu.1
foxn1
ikzf1
rag1
4mm
36 hpf.
36 hpf.
4mm
24 hpf.
24 hpf.
24 hpf.
24 hpf.
1mm
5 dpf.
5 dpf.
5 dpf.

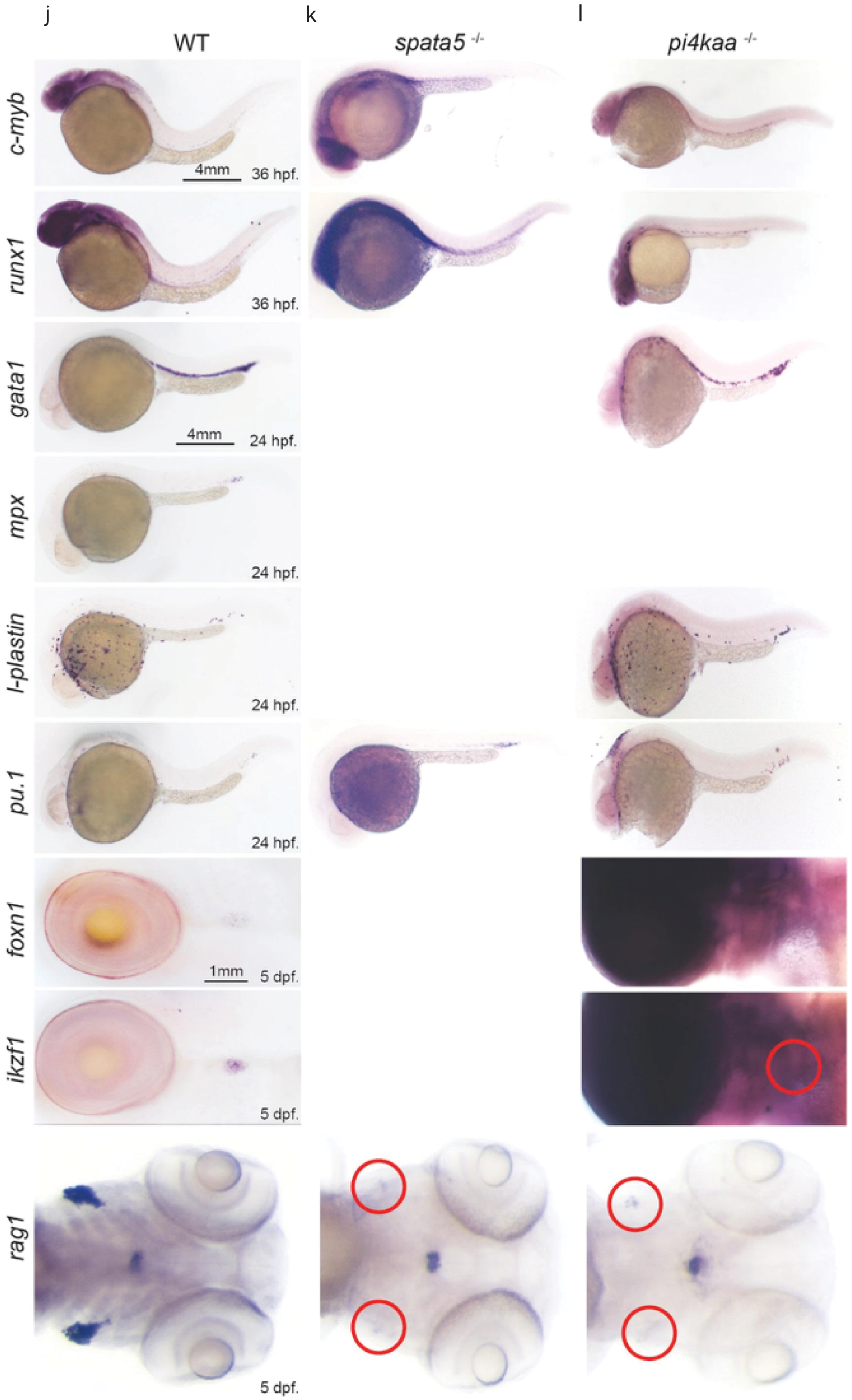
j
k
l
WT
spata5 -/-
pi4kaa -/-
c-myb
runx1
gata1
mpx
l-plastin
pu.1
foxn1
ikzf1
rag1
4mm
36 hpf.
36 hpf.
4mm
24 hpf.
24 hpf.
24 hpf.
24 hpf.
1mm
5 dpf.
5 dpf.
5 dpf.

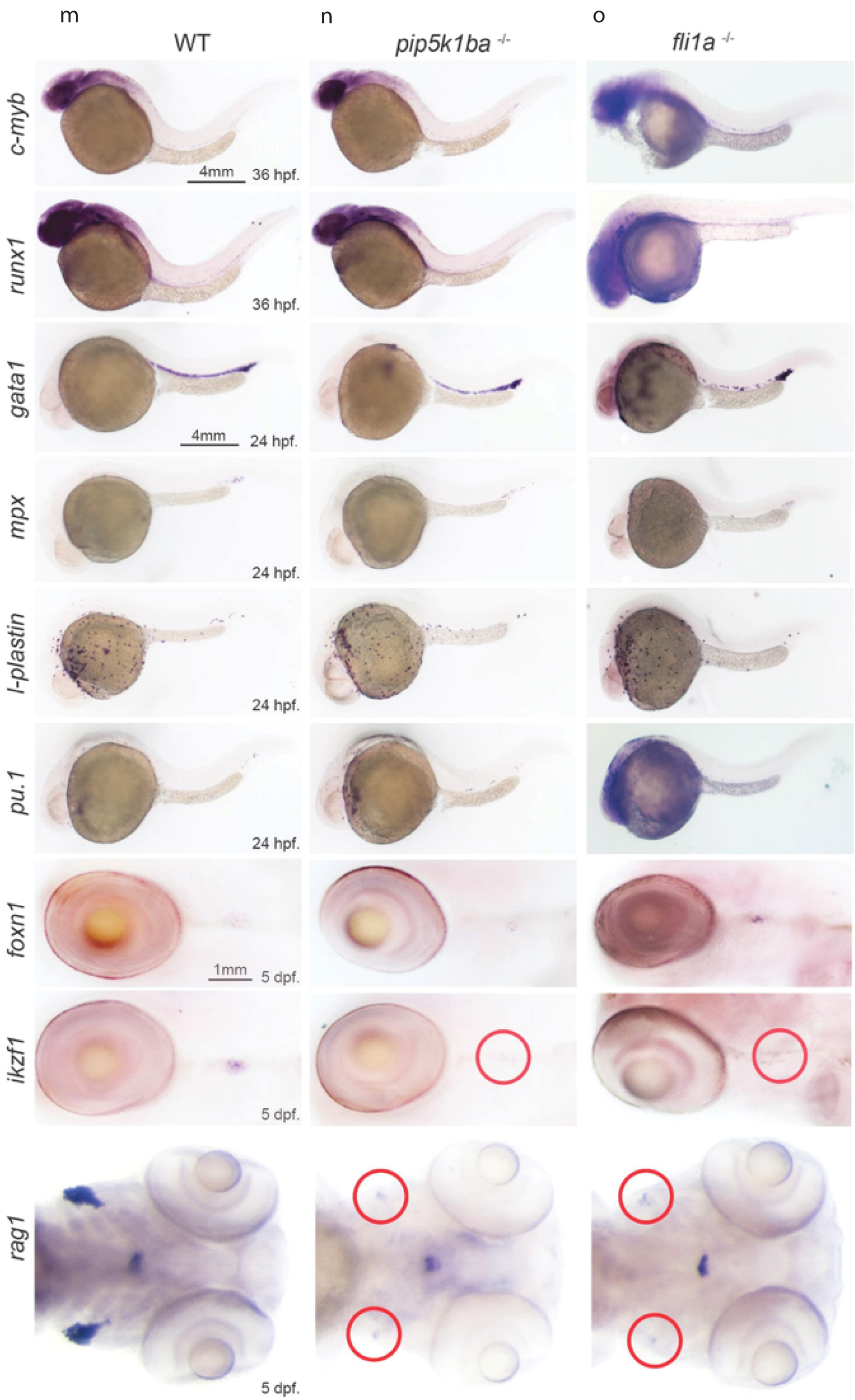

m
WT
n
pip5k1ba -/-
o
fli1a -/-
c-myb
4mm
36 hpf.
runx1
36 hpf.
gata1
4mm
24 hpf.
mpx
24 hpf.
l-plastin
24 hpf.
pu.1
24 hpf.
foxn1
1mm
5 dpf.
ikzf1
5 dpf.
rag1
5 dpf.

Supplementary Figure 3 | Whole mount RNA *in situ* hybridization and expression of genes regulating haematopoiesis. Characterization of selected mutants by RNA *in situ* hybridizations for markers of haematopoiesis, including haematopoietic stem cells (36 h.p.f – *c-myb*, *runx1*), thymic epithelial cells (5 d.p.f – *foxn1*), lymphoid cells (5 d.p.f – *ikzf1*), myeloid cells (24 h.p.f – *pu.1* [*spi1b*]), erythrocytes (24 h.p.f – *gata1*), neutrophils (24 h.p.f – *mpx*), and macrophages (24 d.p.f – *l-plastin* [*lcp1*]). **a**, **d**, **g**, **j**, **m**, wild-type (WT) embryos. **b**, *tbcb*. **c**, *mat2aa*. **e**, *unc45a*. **f**, *ube3d*. **h**, *anapc1*. **i**, *nek7*. **k**, *spata5a*. **l**, *pi4kaa*. **n**, *pip5k1ba*. **o**, *fli1a*. Haematopoietic defects indicated by abnormal hybridization patterns are highlighted by red circles. Panels are representative of >10 embryos per genotype.

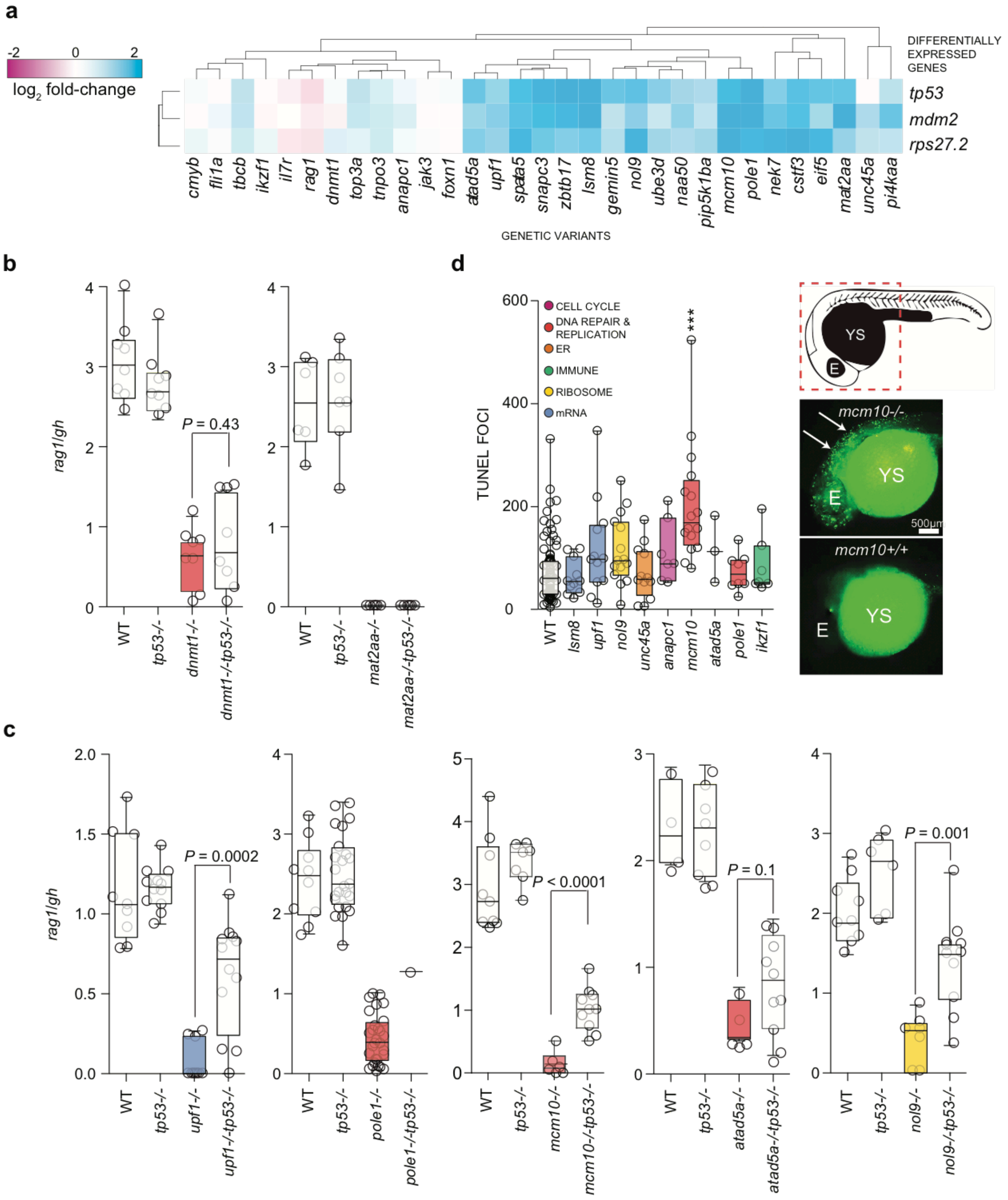
a
-2
0
2
log2 fold-change
DIFFERENTIALLY EXPRESSED GENES
tp53
mdm2
rps27.2
cmyb
fli1a
tbcb
ikzf1
il7r
rag1
dnmt1
top3a
tnpo3
anapc1
jak3
foxn1
atad5a
upf1
spata5
snapc3
zbtb17
lsm8
gemin5
nol9
ube3d
naa50
pip5k1ba
mcm10
pole1
nek7
cstf3
eif5
mat2aa
unc45a
pi4kaa
GENETIC VARIANTS
b
rag1/gh
P = 0.43
WT
tp53-/-
dnmt1-/-
dnmt1-/-tp53-/-
WT
tp53-/-
mat2aa-/-
mat2aa-/-tp53-/-
d
TUNEL FOCI
CELL CYCLE
DNA REPAIR & REPLICATION
ER
IMMUNE
RIBOSOME
mRNA
***
WT
lsm8
upf1
nol9
unc45a
anapc1
mcm10
atad5a
pole1
ikzf1
YS
E
mcm10-/-
500µm
mcm10+/+
c
rag1/gh
P = 0.0002
WT
tp53-/-
upf1-/-
upf1-/-tp53-/-
WT
tp53-/-
pole1-/-
pole1-/-tp53-/-
P < 0.0001
WT
tp53-/-
mcm10-/-
mcm10-/-tp53-/-
P = 0.1
WT
tp53-/-
atad5a-/-
atad5a-/-tp53-/-
P = 0.001
WT
tp53-/-
nol9-/-
nol9-/-tp53-/-

Supplementary Figure 4 | p53 dependency and sensitivity of T cell phenotype of genetic variants. a, Expression of p53 signalling pathway signature genes for all genetic variants. Genetic variants are depicted in columns and components of the p53 pathway are in rows. **b**, Apoptosis determined by the numbers of TUNEL foci from 32 h.p.f. zebrafish mutants. Significance was determined by one-way ANOVA with Dunnett's post-test; ***, P <0.001. Representative images compare a *mcm10* mutant fish embryo displaying neuronal apoptosis (white arrow) compared to an unaffected *mcm10* wild-type sibling. Yolk sack (YS) and eye (E) are labeled to facilitate anatomical orientation of fluorescent image. **c**, No rescue of thymopoietic activity (expressed in *rag1*/*gh* ratio) in *dnmt1* and *mat2aa* genetic variants in the *tp53*-deficient background. P values were determined by two-tailed Student's t-test. **d**, Rescue of failing T cell development (*rag1*/*gh* ratio) in *upf1*, *pole1*, *mcm10*, *atad5a* and *nol9* genetic variants in the *tp53*-deficient background. P values were determined by two-tailed Student's t-test. Note that *pole1* and *tp53* genes, encoding the catalytic subunit of DNA polymerase epsilon and the p53 protein respectively, are situated on the same chromosome in zebrafish (chromosome 5), and are about 12 Mb apart; hence, the number of double mutants arising from double-heterozygous parents is smaller than expected, since meiotic recombination is required to position the two mutant alleles onto the same chromosome. However, when *p53* function is reduced by use of anti-sense morpholino knock-down, the rescue of the *pole1*-induced phenotype is robustly observed. See Source Data for Supplementary Figure 4.

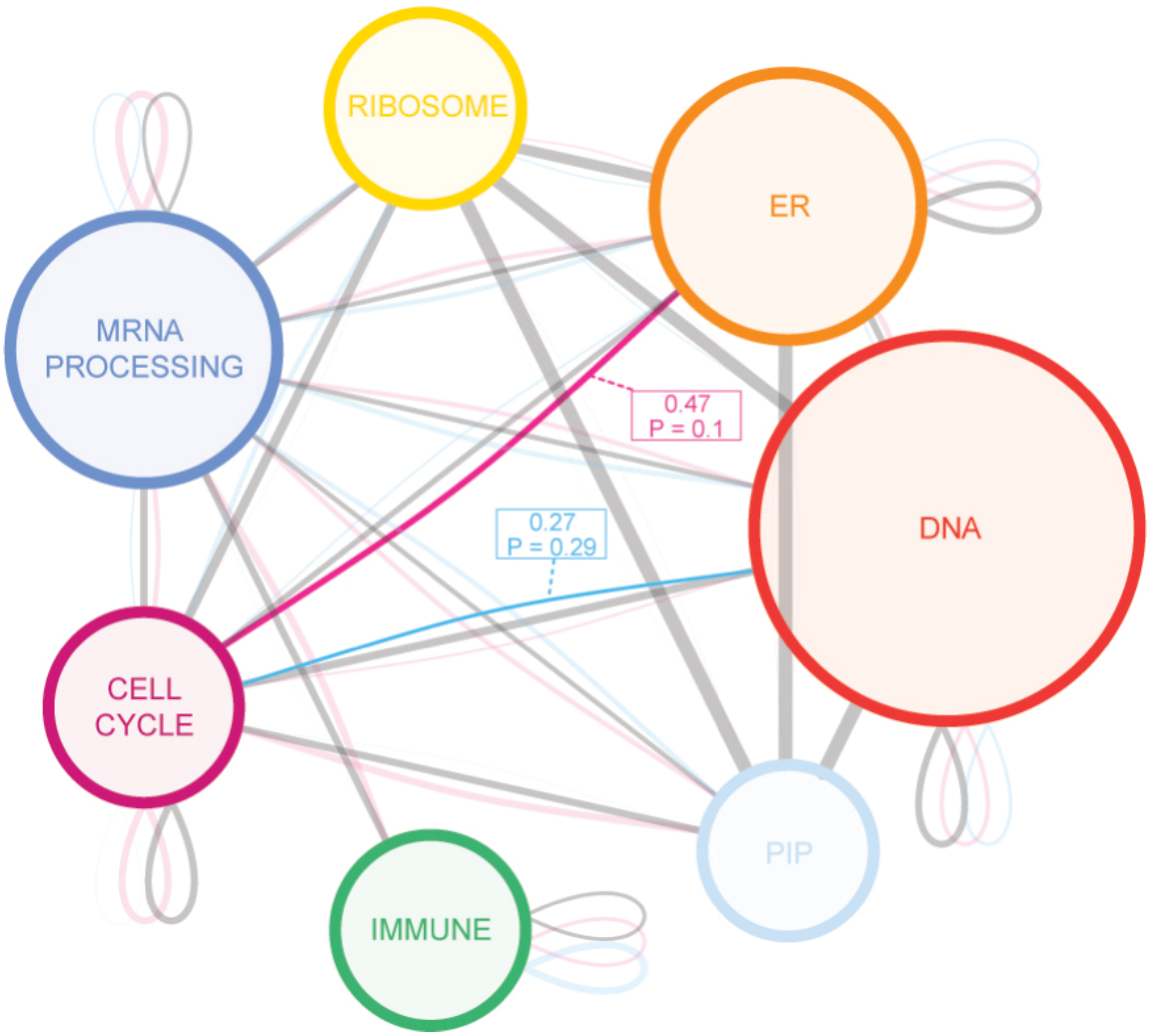

Supplementary Figure 5 | Structure of interaction network and proportion of interactions. Proportions of interactions between individual categories. Nodes are grouped by primary biological pathways affected by mutation or inhibitor. Node size is relative to the number of genes and inhibitors within each biological functional category. For this presentation, positive–suppressive and positive–coequal interactions are combined. Edge thickness is relative to proportion of interaction. *P* values for proportions were obtained by bootstrapping analysis.

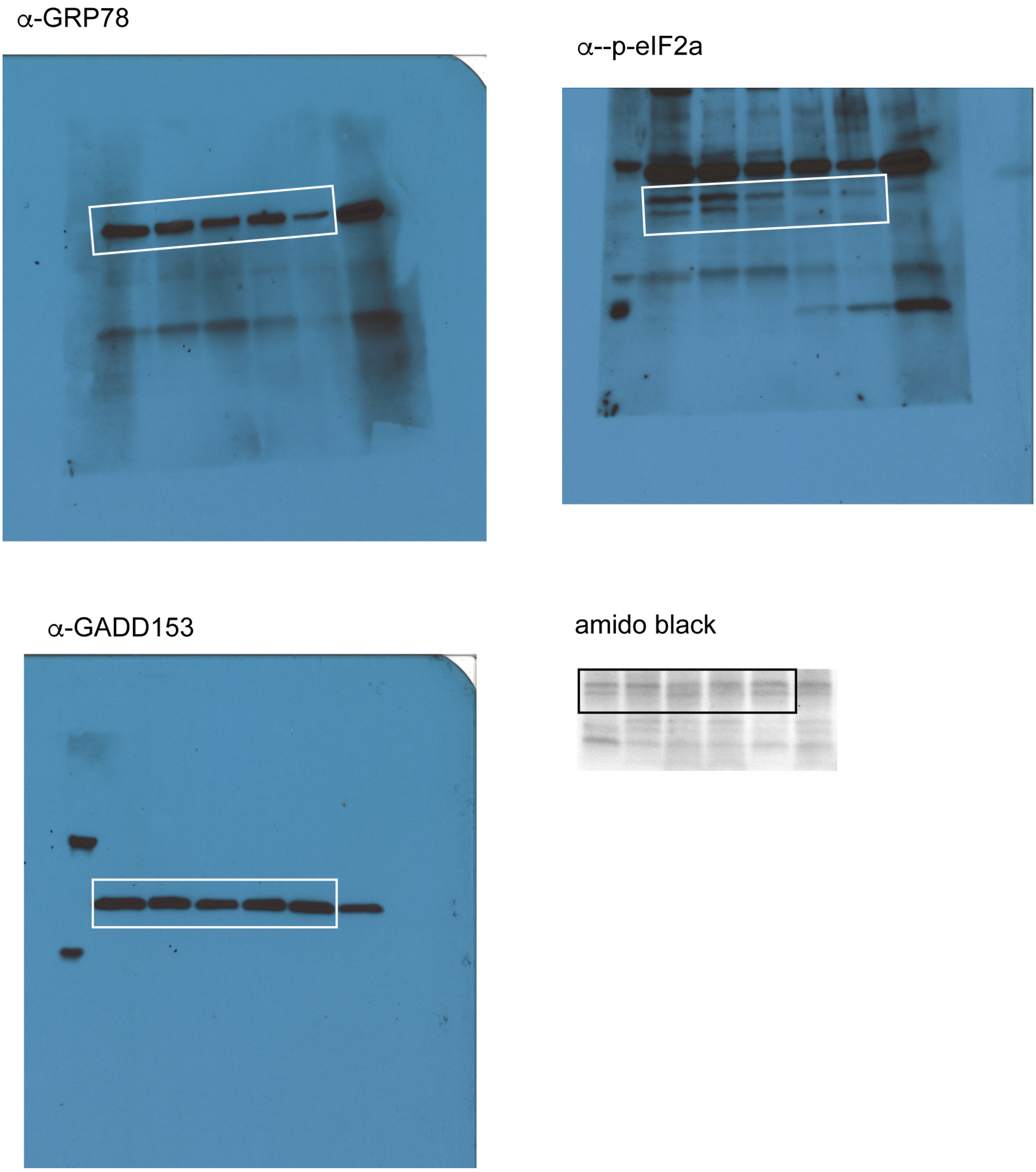

Supplementary Figure 6 | Uncropped Western blots. The cropped areas are shown in Supplementary Figure 2c.

Supplementary Table 1 | Antisense morpholino oligonucleotides used in this study.

Mutant	Affected Gene	ENSEMBL Gene ID[a]	Name[b]	Sequence	Reference
JM087	*anapc1*	ENSDARG00000075687	ZF_ANAPC1_ATG	TGTACCACTTGACCAGCACTTTCAT	This study
JM087	*anapc1*	ENSDARG00000075687	ZF_ANAPC1_ACC5	CTTCATAGACGTGCGACATGAGTTC	This study
HU319	*atad5a*	ENSDARG00000070568	ZF_ATAD5A_ATG	GGCAATGCAACAACCCCAGCCATCT	This study
HU319	*atad5a*	ENSDARG00000070568	ZF_ATAD5A_DON3	AGAAGTGTTGATCTTACCTGATAGG	This study
JM052	*fcf1*	ENSDARG00000102333	ZF_FCF1_DON2	AAGACTCAAACTTACATTTCTTGTT	This study
JM052	*fcf1*	ENSDARG00000102333	ZF_FCF1_ATG	ATGACGGCTGAATTTGTTCGAGATT	This study
JZ061	*fli1a*	ENSDARG00000054632	ZF_FLI1A_ATG	CGCCTCCTTAATAGTTCCGTCCATT	This study
JZ061	*fli1a*	ENSDARG00000054632	ZF_FLI1A_DON7	TTGGAGAGCCTGAGAAATGGAAAGA	This study
KL069	*gemin5*	ENSDARG00000079257	GEMIN5ATG	GATGTCTTTCGTGCATTATATACCG	20
KL069	*gemin5*	ENSDARG00000079257	ZF GEMIN5 DON4	GCACAAAACCTCTAGTTTACCTGCA	20
KL069	*gemin5*	ENSDARG00000079257	ZF GEMIN5 ACC9	ATGCCAACTGTAAGAAAAGTGTGGA	20
18_10	*lsm8*	ENSDARG00000091656	ZF_LSM8 ACC4	CGATCACAGCCCTTAAACACAAAAT	20
18_10	*lsm8*	ENSDARG00000091656	ZF_LSM8 ACC3	CGTCCCCTAAAAACAGCACAAGTCA	20
HY062	*mat2aa*	ENSDARG00000040334	ZF_MAT2AA_ATG	AGCCGTTCAGTTGTCCGTTCATATT	This study
HY062	*mat2aa*	ENSDARG00000040334	ZF_MAT2AA_ACC4	ACCCTTAAAGTACAACACAGGGATT	This study
IG335	*mcm10*	ENSDARG00000045815	ZF_MCM10 ACC4	TCTGAAGAGGCTGATTTACATAAGA	This study
JI073	*naa50*	ENSDARG00000027825	ZF_NAA50_ACC2	CCGGCTACTAGAACAAAAGCAGAAT	This study
JI073	*naa50*	ENSDARG00000027825	ZF_NAA50_DON2	AGCGTTGTTACATACCTAGCTTGGC	This study
IT429	*nek7*	ENSDARG00000056966	ZF_NEK7_DON8	GATGGGTTTCTATACCTTACCTCAT	This study
IT429	*nek7*	ENSDARG00000056966	ZF_NEK7_ATG	CGTCCATTGTGACAGCAGCAGTCGC	This study
HP327	*nol9*	ENSDARG00000077751	ZF_NOL9 ACC4	AGCACTATATTTACCGAGTTGAGGC	This study
HP327	*nol9*	ENSDARG00000077751	ZF_NOL9 ATG (3RD_1)	GCTGACCCCCAACGAGACTATAAAC	This study
HG002	*pi4kaa*	ENSDARG00000076724	ZF_PI4KAA_ACC22	AGCTCAGCCTGGAAACAGCAAATGT	This study
HG002	*pi4kaa*	ENSDARG00000076724	ZF_PI4KAA_ATG	ACGTCCCTCTCGACGACATTATTCA	This study
IG447	*pip5k1ba*	ENSDARG00000044295	ZF_PIP5K1BA_DON5	TTGTGGATTGTGTGGCTCACCATGT	This study
IG447	*pip5k1ba*	ENSDARG00000044295	ZF_PIP5K1BA_ATG	GCTCATCTGCCGTTGCACTCATCTT	This study
JI065	*pnrc1*	ENSDARG00000043904	ZF_PNRC1_ACC2	GGCTGCTTTAGACAAACATGAAACA	This study
JI065	*pnrc1*	ENSDARG00000043904	ZF_PNRC1_ATG	GACGACCAAAAGCATCGCCCAACAT	This study
HG010	*pole1*	ENSDARG00000058532	ZF_POLE_ATG	GTCTGAAGACTTTCAAATCAGTTAC	20
HG010	*pole1*	ENSDARG00000058533	ZF_POLE_ACC17	ATCACACACCTGAAACAGGAAAAAT	20

HG010	*pole1*	ENSDARG00000058533	ZF_POLE_DON13	GATGAAAATTAGACCTGTGGTTCT	20
KW059	*snapc3*	ENSDARG00000101474	ZF_SNAPC3_ATG	TCTTTGCGTATCTCCGCCATAATTC	20
KW059	*snapc3*	ENSDARG00000101474	ZF_SNAPC3_ACC7	ATTACCCTTCAGCAAGAACACATAT	20
KH025	*eif5*	ENSDARG00000003681	ZF_EIF5_DON4	AATTTAATACTCACATGTCGGAGGC	This study
IG438	*spata5*	ENSDARG00000104869	ZF_SPATA5_ACC16	GACCACCAATATCACTCCACTTCAC	This study
IG438	*spata5*	ENSDARG00000104869	ZF_SPATA5_ATG	CTTTTCTTACTGGATGACATGATGC	This study
HI020	*tbcb*	ENSDARG00000068404	ZF_TBCB_ATG	GATTGTCACACTCCCGTCCATCTTC	This study
HI020	*tbcb*	ENSDARG00000068404	ZF_TBCB_ACC6	GCCGTACCTGAAAACAATAGAAGCA	This study
HA343	*tnpo3*	ENSDARG00000045680	TNPO3 ATG	GGTTTCCCGCCTTCCATGGTGCTCT	20
HA343	*tnpo3*	ENSDARG00000045680	TNPO3 SPLICE ACC6	TCATCCCTCTGCTTCAATGACGAGT	20
IM087	*ube3d*	ENSDARG00000026178	ZF_UBE3D_ACC9	CAACACTACACATCAGGGAAAAACA	This study
IM087	*ube3d*	ENSDARG00000026178	ZF_UBE3D_ATG	TCGCAGTCTCTTCCATTGGTATTTC	This study
IL015	*unc45a*	ENSDARG00000103643	ZF_UNC45A_ACC2/ATG	CTGGGACATCTACACAGTCAGAAAA	This study
HJ028	*upf1*	ENSDARG00000016302	ZF_UPF1_ACC2	GTTCACCTGAAAACAAGATGAGCAA	91
JZ007	*yeats2*	ENSDARG00000078767	ZF_YEATS2_DON25	AGAAACTGGCACACACTTACCTGGT	This study
JZ007	*yeats2*	ENSDARG00000078767	ZF_YEATS2_ACC23	CCGTGCTGAGGGAGATTGATAATAA	This study

[a] Zv10

[b] Morpholinos target translation initiation codon (ATG) or splice sites (DON – donor, ACC – acceptor, numbers refer to exons)

Supplementary Table 2 | BAC constructs and mRNA sources used for phenotypic rescue experiments.

Mutant	Affected Gene	ENSEMBL Gene ID	Rescue type	Clone	Species	Accession
HU319	*Atad5a*	ENSMUSG00000017550	BAC	RP13-753N3	Mouse	AC130324
HG002	*Pi4ka*	ENSMUSG00000041720	BAC	RP23-322A15	Mouse	AC110573
IG438	*Spata5*	ENSMUSG00000027722	mRNA	RIKEN 2510048F20	Mouse	AK011111
HI020	*Tbcb*	ENSMUSG00000006095	mRNA	IMAGE 2648112	Mouse	BC010684
KH025	*Eif5*	ENSMUSG00000021282	BAC	RIKEN 2810029C07	Mouse	AC163357
IM087	*Ube3d*	ENSRNOG00000010802	mRNA	IMAGE 7935691	Rat	BC101916
IL015	*Unc45a*	ENSMUSG00000030533	mRNA	IMAGE 35882116	Mouse	BC004717

Supplementary Table 3 | CRISPR/Cas9 guide RNAs used in this study to generate gene-specific mutations.

Affected Gene[a]	ENSEMBL Gene ID	Forward primer[b]	Reverse primer[b]	Molecular Defect[c]	Mutation type
spata5	ENSDARG00000104869	**TAGG**TTACTGAGTAAATATGT	**AAAC**ACATATTTACTCAGTAA	g.11570-11840del	frameshift deletion mutation
pi4kaa	ENSDARG00000076724	**TAGG**CGTGAAGGCCAGCTCCA	**AAAC**TGGAGCTGGCCTTCACG	p.S630-M632del	frameshift deletion mutation
foxn1	ENSDARG00000011879	**TAGG**TTGGAGGAACAATCCTCT	**AAAC**AGAGGATTGTTCCTCCAA	p.D130-A279del.AATCGCCins	frameshift deletion mutation
	ENSDARG00000011879	**TAGG**CCCTGAACCCAGCGAAGG	**AAAC**CCTTCGCTGGGTTCAGGG		
ikzf1	ENSDARG00000013539	**TAGG**TGCTTCATTCACTCAGAA	**AAAC**TTCTGAGTGAATGAAGCA	p.F171-R274del	frameshift deletion mutation
	ENSDARG00000013539	**TAGG**ACATGCCTGCATCTGAGA	**AAAC**TCTCAGATGCAGGCATGT		

[a] One (*spata5*, *pi4kaa*) or two (*foxn1*, *ikzf1*) guide RNAs were used in generating CRISPR mutants
[b] The pDR274 vector was cut with *Dra*I; the overlap at restriction site is in bold letters
[c] Nomenclature according to Ref. 92.

Supplementary Table 4 | Genotyping primers used in this study.

Affected Gene	Primer	Type
fli1a	ATTTCTCAGGCTCTCCAACAG	Forward
	TAGCAAGTCGACTGCTGGTG	Reverse
pole	GTCTGTGGACATTTGATGCTTG	Forward
	GACTCCAGCTTGGACCCAC	Reverse
tbcb	ATGAGGAAGAGAGGGCCAAG	Forward
	CCACTGCTGTTAGGTACATC	Reverse
unc45a	GGGAGCCAAATAGTATTCAAG	Forward
	GCGGTACAGGACTGCACTCT	Reverse
pnrc1	CATAGACAAGACATCACCTG	Forward
	TGCTTCAGGATGTTTTCTGG	Reverse
ube3d	TGGATGTGTGGGAGAAGGAC	Forward
	TCAGAGTGGTGTGTGACCTG	Reverse
naa50	TGCACTGCTGGTTTACGGTG	Mutant
	TTAGGCTCTGTGTTGCATGTG	Mutant
	GTCAGTTCACAGCTAGTTGAC	Wildtype
	GTTGAGTTACGGCTTTGTTGTG	Wildtype
yeats2	GTCAAAGTAGAACAGGGC	Forward
	ATTCCCTCTGATTGTCCC	Reverse
atad5a	GACAGGCTCTTCAGTGTTGTC	Forward
	CAGCTTCAAGAGCAAGTCCTG	Reverse
anapc1	CAGCAGGGCGACTCATTTTG	Forward
	CTGAACTGGGCTGTCGACTG	Reverse
nek7	CAATTGAACCACCCCAATGT	Forward
	AATGGGCATGTGTCCTTACC	Reverse
spata5	GTCCGCAGGGTCCAGAGTTAC	Forward
	TGACGGAGCAACAGTTCTGG	Reverse
mat2aa	CCCAACTAACCAAGCCAAGTT	Forward
	AGTCTCGCTAGTGGCATAAC	Reverse
nol9	CCAACAGTGTTCTTCAGAACG	Forward
	ATGTGGATTGGACCTGGAAAC	Reverse
eif5	GCTCTAAATAGGCCTCCGACA	Forward

	CAGTGCATCAAGGGTACACAG	Reverse
pi4kaa	AAGGTGGAGTGTTGCTTTAAG	Forward
	CGTGACAGTGTCGTTCTTCAG	Reverse
pip5k1ba	ACTGAAACACAATCAAGCAAGTG	Forward
	CTGTTGCTAAAGACATGTTGTG	Reverse

Supplementary Table 5 | Small molecule inhibitors used in this study.

Inhibitor	Abbrev.	Cat#	Target	Reference	IC30 ± SE[a]
**DNA Replication/Repair**					
NU7026	NU7	SEL-S2893	DNA-PK	37	6.56 ± 1.14 μM
Etoposide	ETO	AG-CR1-3572	DNA topoisomerase II	77	0.95 ± 0.08 μM
Doxorubicin	DOX	1527-5	DNA topoisomerase II	78	0.53 ± 0.08 μM
Mitoxantrone Dihydrochloride	MD	sc-203136	DNA topoisomerase II	79	1.91 ± 0.4 μM
5-Fluorouracil	5FU	F6627	Aminoisobutyrate-pyruvate aminotransferase	80	207.82 ± 35.93 μM
**Cell Cycle Regulation**					
Nocodazole	NOC	10762633	Tubulin	81	0.2 ± 0.03 μM
Chr-6494	CHR	372040	Hapsin histone kinase	82	0.38 ± 0.09 μM
**mRNA Processing**					
Pladienolide B	PB	5301960001	SF3b	36	0.07 ± 0.01 μM
Isoginkgetin	ISO	416154	U4/U5/U6 tri-small nuclear ribonucleoprotein	83	175.52 ± 23.49 μM
NMD Inhibitor 14	NMD	5308380001	SMG7-UPF1	84	13.01 ± 3.65 μM
**Chaperone & Protein Transport**					
Thapsigargin	THS	sc-24017A	Ca2+ ATPase	32	0.16 ± 0.02 μM
Tunicamycin	TUN	T7765	N-linked glycosylation	85	1.13 ± 0.25 μM
Eeyarestatin I	EEY	324521	SEC61	86	787.08 ± 59.03 μM
Brefeldin A	BFA	BML-G405	HDL-mediated cholesterol efflux	87	0.51 ± 0.11μM

[a] Treatment period: 72 - 120 h.p.f

Supplementary Table 6 | Inhibitor concentration for treatment of adolescent fish and treatment outcomes.

Abbreviation	Inhibitor	Dosage	Inhibitor 2	Dosage	log2 Δ *lck* ± SD (n)[a]	log2 Δ *Myc* ± SD (n)[b]
DMSO	-	0.00083%	-	-	1.27 ± 0.21 (23)	2.23 ± 3.63 (43)
THS	Thapsigargin[c]	25 nM	-	-	-	1.21 ± 0.16 (22)
PB	Pladienolide B[d]	66.7 nM	-	-	-	2.96 ± 4.12 (22)
NU7	NU7026[e]	899.2 nM	-	-	-	1.4 ± 0.45 (24)
PB + NU7	Pladienolde B	66.7 nM	NU7026	366.7 nM	-1.46 ± 0.43 (23)	-1.26 ± 0.45 (23)
THS + NU7	Thapsigargin	25 nM	NU7026	899.2 nM	-0.27 ± 0.03 (23)	-0.24 ± 0.72 (22)

[a] % Δ *lck* refers to the $\log_2$-fold change (mean ± standard deviation) of *lck*-CFP signal between D0 and D21; n= number of fish
[b] % Δ *Myc* refers to the $\log_2$-fold change (mean ± standard deviation) of *Myc*-CFP signal between D0 and D21; n= number of fish
[c] Administered to human patients (clinical trial # NCT01056029).
[d] Previously used for *in vitro* and *in vivo* treatment of tumour cell lines (Ref. 36, 88)
[e] Administered to human patients (clinical trial # NCT02316197).
- , not done
