# Supplementary material for: Genetic landscape of T cells identifies synthetic lethality for T-ALL
Source: Commun Biol. 2021 Oct 20;4:1201. doi: 10.1038/s42003-021-02694-x (PMC8528931; doi:10.1038/s42003-021-02694-x)
Supplement: Supplementary file 2 — Description of Additional Supplementary Files [file 42003_2021_2694_MOESM2_ESM.pdf]

Description of Additional Supplementary Files

File name: Supplementary Data 1.
Description: R scripts, raw data and annotation files.

File name: Supplementary Data 2.
Description: Source data.
