# Supplementary material for: Genetic landscape of T cells identifies synthetic lethality for T-ALL
Source: Commun Biol. 2021 Oct 20;4:1201. doi: 10.1038/s42003-021-02694-x (PMC8528931; doi:10.1038/s42003-021-02694-x)
Supplement: Supplementary file 5 — Reporting Summary [file 42003_2021_2694_MOESM5_ESM.pdf]

nature research

Corresponding author(s): Thomas Boehm

Last updated by author(s): Aug 13, 2021

Reporting Summary

Nature Research wishes to improve the reproducibility of the work that we publish. This form provides structure for consistency and transparency in reporting. For further information on Nature Research policies, see our Editorial Policies and the Editorial Policy Checklist.

Statistics

For all statistical analyses, confirm that the following items are present in the figure legend, table legend, main text, or Methods section.

n/a	Confirmed	
☐	☒	The exact sample size (*n*) for each experimental group/condition, given as a discrete number and unit of measurement
☐	☒	A statement on whether measurements were taken from distinct samples or whether the same sample was measured repeatedly
☐	☒	The statistical test(s) used AND whether they are one- or two-sided<br>*Only common tests should be described solely by name; describe more complex techniques in the Methods section.*
☒	☐	A description of all covariates tested
☐	☒	A description of any assumptions or corrections, such as tests of normality and adjustment for multiple comparisons
☐	☒	A full description of the statistical parameters including central tendency (e.g. means) or other basic estimates (e.g. regression coefficient) AND variation (e.g. standard deviation) or associated estimates of uncertainty (e.g. confidence intervals)
☐	☒	For null hypothesis testing, the test statistic (e.g. *F*, *t*, *r*) with confidence intervals, effect sizes, degrees of freedom and *P* value noted<br>*Give P values as exact values whenever suitable.*
☒	☐	For Bayesian analysis, information on the choice of priors and Markov chain Monte Carlo settings
☒	☐	For hierarchical and complex designs, identification of the appropriate level for tests and full reporting of outcomes
☒	☐	Estimates of effect sizes (e.g. Cohen's *d*, Pearson's *r*), indicating how they were calculated

Our web collection on statistics for biologists contains articles on many of the points above.

Software and code

Policy information about availability of computer code

Data collection	No software used for data collection
Data analysis	For a description of software/codes used for the present study, see https://github.com/connoromeara/OMeara_et_al_2021.

For manuscripts utilizing custom algorithms or software that are central to the research but not yet described in published literature, software must be made available to editors and reviewers. We strongly encourage code deposition in a community repository (e.g. GitHub). See the Nature Research guidelines for submitting code & software for further information.

Data

Policy information about availability of data

All manuscripts must include a data availability statement. This statement should provide the following information, where applicable:

- Accession codes, unique identifiers, or web links for publicly available datasets
- A list of figures that have associated raw data
- A description of any restrictions on data availability

R scripts, raw data and annotation files are included in the GitHub repository (https://github.com/connoromeara/OMeara_et_al_2021). Paired-end raw RNA-Seq reads from mutant lines, normalised counts and differential gene expression output from DESeq2 can be found at NCBI Gene Expression Omnibus (GEO) (GSE147555). Paired-end raw WGS-seq reads from mutant lines can be found in NCBI Sequence Read Archive (SRA) (PRJNA622735). Uncropped Western blots are provided as Supplementary Figure 6.

Field-specific reporting

Please select the one below that is the best fit for your research. If you are not sure, read the appropriate sections before making your selection.

☒ Life sciences ☐ Behavioural & social sciences ☐ Ecological, evolutionary & environmental sciences

For a reference copy of the document with all sections, see nature.com/documents/nr-reporting-summary-flat.pdf

Life sciences study design

All studies must disclose on these points even when the disclosure is negative.

Sample size	Sample sizes were chosen based on community standards.
Data exclusions	No data were excluded.
Replication	Several biological replicates were included in the study design.
Randomization	Not relevant
Blinding	Samples were coded, and un-blinded after analysis.

Reporting for specific materials, systems and methods

We require information from authors about some types of materials, experimental systems and methods used in many studies. Here, indicate whether each material, system or method listed is relevant to your study. If you are not sure if a list item applies to your research, read the appropriate section before selecting a response.

Materials & experimental systems

n/a	Involved in the study	
☐	☒	Antibodies
☒	☐	Eukaryotic cell lines
☒	☐	Palaeontology and archaeology
☐	☒	Animals and other organisms
☒	☐	Human research participants
☒	☐	Clinical data
☒	☐	Dual use research of concern

Methods

n/a	Involved in the study	
☒	☐	ChIP-seq
☐	☒	Flow cytometry
☒	☐	MRI-based neuroimaging

Antibodies

Antibodies used	rabbit α-GRP78/HSP5 (1:2000, Cat#PA524963, Life Technologies), rabbit α-GADD153/CHOP (1:2000, Cat#G6916, Sigma-Aldrich), rabbit α-eIF2a (1:1000, Cat# PA5-41916, ThermoFisherScientific), mouse α-vertebrate-β-actin (1:2000, Cat#A2066, Sigma-Aldrich) goat α-rabbit-HRP secondary (1:2000, Cat#P0448, ThermoFisherScientific), goat α-mouse-HRP secondary (1:2000, Cat# P0447, ThermoFisherScientific)
Validation	see information associated with catalog numbers; https://www.thermofisher.com; https://www.sigmaaldrich.com

Animals and other organisms

Policy information about studies involving animals; ARRIVE guidelines recommended for reporting animal research

Laboratory animals	zebrafish (Danio rerio) strains Ekkwill (EKK), Tüpfel long fin (TL), wild-type-in-Kalkutta (WIK), AB, Assam (ASS), and Tubingen (TU); ikzf1-GFP transgenic zebrafish (B. Bajoghli et al., Evolution of genetic networks underlying the emergence of thymopoiesis in vertebrates. Cell 138, 186-197 (2009)); lck:CFP transgenic zebrafish (O. B. Giorgetti et al., Antigen receptor repertoires of one of the smallest known vertebrates. Sci. Adv. 7, abd8180 DOI: 10.1126/sciadv.abd8180 (2021); rag1 mutant (E. Wienholds, S. Schulte-Merker, B. Walderich, R. H. Plasterk, Target-selected inactivation of the zebrafish rag1 gene. Science 297, 99-102 (2002)). rag2:Myc-GPF transient leukaemia model (A. Gutierrez et al., Pten mediates Myc oncogene dependence in a conditional zebrafish model of T cell acute lymphoblastic leukemia. J. Exp. Med. 208, 1595-1603 (2011)).
Wild animals	not applicable
Field-collected samples	not applicable

Ethics oversight

All animal experiments were approved by the institute's review committee and conducted under licenses from the local governments (Regierungspräsidium Freiburg [AZ 35-9185.81/G-19/69; AZ 35-9185.81/G-14/41; AZ 35-9185.81/G-17/79; AZ 35-9185.81/G-13/70]; Regierungspräsidium Tübingen [AZ AP1/02]).

Note that full information on the approval of the study protocol must also be provided in the manuscript.

Flow Cytometry

Plots

Confirm that:

☒ The axis labels state the marker and fluorochrome used (e.g. CD4-FITC).

☐ The axis scales are clearly visible. Include numbers along axes only for bottom left plot of group (a 'group' is an analysis of identical markers).

☐ All plots are contour plots with outliers or pseudocolor plots.

☐ A numerical value for number of cells or percentage (with statistics) is provided.

Methodology

Sample preparation

The yolk sac was removed from embryos by gently aspirating in 0.5X Ginzburg Fish Ringer (55 mM NaCl, 1.8 mM KCl, 1.25 mM $NaHCO_3$, without Ca^{2+}). Embryos were digested in CO_2-independent media (Life Technologies, Cat# 18045088) containing 3 mg/mL of Collagenase II (Santa Cruz, Cat#sc-506177) at 37°C under gentle rotation. Cell suspension was passed through a 70 µm cell strainer (BD, Cat#352350), washed with PBS and pelleted at 2,800xg. Cells were stained for viability using ZombieRed Fixable Viability Kit (Biolegend, Cat# 423110) for 20 min at RT protected from light. Cells were washed with FACS buffer (PBST, 1% BSA w/v), fixed and permeabilised with FoxP3/TF Staining Buffer Set (eBioscience, Cat#00-5523-00) according to the manufacturer's instructions. Cells were stained with 1µg/mL Hoechst 33258 (ThermoFisherScientific, Cat#33258) at 4°C for 15 min and analysed using the BD Fortessa II and FACSDiva Software on a linear scale. Wild-type fish treated with 0.1 µg/mL Nocodazole or 3 µM Etoposide were used as G2/M and S phase inhibitor controls, respectively.

Instrument

BD Fortessa II

Software

FACSDiva Software on a linear scale.

Cell population abundance

not applicable

Gating strategy

see Supplementary Figure 2a

☒ Tick this box to confirm that a figure exemplifying the gating strategy is provided in the Supplementary Information.
