# Supplementary material for: Author Correction: Genetic landscape of T cells identifies synthetic lethality for T-ALL
Source: Commun Biol. 2024 Feb 14;7:182. doi: 10.1038/s42003-024-05841-2 (PMC10866859; doi:10.1038/s42003-024-05841-2)
Supplement: Supplementary file 1 — Supplementary_Table_1_CORRECTED [file 42003_2024_5841_MOESM1_ESM.docx]

| **Supplementary Table 1 \| Antisense morpholino oligonucleotides used in this study.** | | | | | |
| --- | --- | --- | --- | --- | --- |
| **Mutant** | **Affected Gene** | **ENSEMBL Gene ID^a^** | **Name^b^** | **Sequence** | **Reference** |
| JM087 | *anapc1* | ENSDARG00000075687 | ZF_ANAPC1_ATG | TGTACCACTTGACCAGCACTTTCAT | This study |
| JM087 | *anapc1* | ENSDARG00000075687 | ZF_ANAPC1_ACC5 | CTTCATAGACGTGCGACATGAGTTC | This study |
| HU319 | *atad5a* | ENSDARG00000070568 | ZF_ATAD5A_ATG | GGCAATGCAACAACCCCAGCCATCT | This study |
| HU319 | *atad5a* | ENSDARG00000070568 | ZF_ATAD5A_DON3 | AGAAGTGTTGATCTTACCTGATAGG | This study |
| JM052 | *fcf1* | ENSDARG00000102333 | ZF_FCF1_DON2 | AAGACTCAAACTTACATTTCTTGTT | This study |
| JM052 | *fcf1* | ENSDARG00000102333 | ZF_FCF1_ATG | ATGACGGCTGAATTTGTTCGAGATT | This study |
| JZ061 | *fli1a* | ENSDARG00000054632 | ZF_FLI1A_ATG | CGCCTCCTTAATAGTTCCGTCCATT | This study |
| JZ061 | *fli1a* | ENSDARG00000054632 | ZF_FLI1A_DON7 | TTGGAGAGCCTGAGAAATGGAAAGA | This study |
| KL069 | *gemin5* | ENSDARG00000079257 | GEMIN5ATG | GATGTCTTTCGTGCATTATATACCG | 20 |
| KL069 | *gemin5* | ENSDARG00000079257 | ZF GEMIN5 DON4 | GCACAAAACCTCTAGTTTACCTGCA | 20 |
| KL069 | *gemin5* | ENSDARG00000079257 | ZF GEMIN5 ACC9 | ATGCCAACTGTAAGAAAAGTGTGGA | 20 |
| 18_10 | *lsm8* | ENSDARG00000091656 | ZF_LSM8 ACC4 | CGATCACAGCCCTTAAACACAAAAT | 20 |
| 18_10 | *lsm8* | ENSDARG00000091656 | ZF_LSM8 ACC3 | CGTCCCCTAAAAACAGCACAAGTCA | 20 |
| HY062 | *mat2aa* | ENSDARG00000040334 | ZF_MAT2AA_ATG | AGCCGTTCAGTTGTCCGTTCATATT | This study |
| HY062 | *mat2aa* | ENSDARG00000040334 | ZF_MAT2AA_ACC4 | ACCCTTAAAGTACAACACAGGGATT | This study |
| IG335 | *mcm10* | ENSDARG00000045815 | ZF_MCM10 ACC4 | TCTGAAGAGGCTGATTTACATAAGA | This study |
| JI073 | *naa50* | ENSDARG00000027825 | ZF_NAA50_ACC2 | CCGGCTACTAGAACAAAAGCAGAAT | This study |
| JI073 | *naa50* | ENSDARG00000027825 | ZF_NAA50_DON2 | AGCGTTGTTACATACCTAGCTTGGC | This study |
| IT429 | *nek7* | ENSDARG00000056966 | ZF_NEK7_DON8 | GATGGGTTTCTATACCTTACCTCAT | This study |
| IT429 | *nek7* | ENSDARG00000056966 | ZF_NEK7_ATG | CGTCCATTGTGACAGCAGCAGTCGC | This study |
| HP327 | *nol9* | ENSDARG00000077751 | ZF_NOL9 ACC4 | AGCACTATATTTACCGAGTTGAGGC | This study |
| HP327 | *nol9* | ENSDARG00000077751 | ZF_NOL9 ATG (3RD_1) | GCTGACCCCCAACGAGACTATAAAC | This study |
| HG002 | *pi4kaa* | ENSDARG00000076724 | ZF_PI4KAA_ACC22 | AGCTCAGCCTGGAAACAGCAAATGT | This study |
| HG002 | *pi4kaa* | ENSDARG00000076724 | ZF_PI4KAA_ATG | ACGTCCCTCTCGACGACATTATTCA | This study |
| IG447 | *pip5k1ba* | ENSDARG00000044295 | ZF_PIP5K1BA_DON5 | TTGTGGATTGTGTGGCTCACCATGT | This study |
| IG447 | *pip5k1ba* | ENSDARG00000044295 | ZF_PIP5K1BA_ATG | GCTCATCTGCCGTTGCACTCATCTT | This study |
| JI065 | *pnrc1* | ENSDARG00000043904 | ZF_PNRC1_ACC2 | GGCTGCTTTAGACAAACATGAAACA | This study |
| JI065 | *pnrc1* | ENSDARG00000043904 | ZF_PNRC1_ATG | GACGACCAAAAGCATCGCCCAACAT | This study |
| HG010 | *pole1* | ENSDARG00000058532 | ZF_POLE_ATG | GTCTGAAGACTTTCAAATCAGTTAC | 20 |
| HG010 | *pole1* | ENSDARG00000058533 | ZF_POLE_ACC17 | ATCACACACCTGAAACAGGAAAAAT | 20 |
| HG010 | *pole1* | ENSDARG00000058533 | ZF_POLE_DON13 | GATGAAAATTAGACCTGTGGTTCT | 20 |
| KW059 | *snapc3* | ENSDARG00000101474 | ZF_SNAPC3_ATG | TCTTTGCGTATCTCCGCCATAATTC | 20 |
| KW059 | *snapc3* | ENSDARG00000101474 | ZF_SNAPC3_ACC7 | ATTACCCTTCAGCAAGAACACATAT | 20 |
| KH025 | *eif5* | ENSDARG00000003681 | ZF_EIF5_DON4 | AATTTAATACTCACATGTCGGAGGC | This study |
| IG438 | *spata5* | ENSDARG00000104869 | ZF_SPATA5_ACC16 | GACCACCAATATCACTCCACTTCAC | This study |
| IG438 | *spata5* | ENSDARG00000104869 | ZF_SPATA5_ATG | CTTTTCTTACTGGATGACATGATGC | This study |
| HI020 | *tbcb* | ENSDARG00000068404 | ZF_TBCB_ATG | GATTGTCACACTCCCGTCCATCTTC | This study |
| HI020 | *tbcb* | ENSDARG00000068404 | ZF_TBCB_ACC6 | GCCGTACCTGAAAACAATAGAAGCA | This study |
| HA343 | *tnpo3* | ENSDARG00000045680 | TNPO3 ATG | GGTTTCCCGCCTTCCATGGTGCTCT | 20 |
| HA343 | *tnpo3* | ENSDARG00000045680 | TNPO3 SPLICE ACC6 | TCATCCCTCTGCTTCAATGACGAGT | 20 |
| IM087 | *ube3d* | ENSDARG00000026178 | ZF_UBE3D_ACC9 | CAACACTACACATCAGGGAAAAACA | This study |
| IM087 | *ube3d* | ENSDARG00000026178 | ZF_UBE3D_ATG | TCGCAGTCTCTTCCATTGGTATTTC | This study |
| IL015 | *unc45a* | ENSDARG00000103643 | ZF_UNC45A_ACC2/ATG | CTGGGACATCTACACAGTCAGAAAA | This study |
| HJ028 | *upf1* | ENSDARG00000016302 | ZF_UPF1_ACC2 | GTTCACCTGAAAACAAGATGAGCAA | 91 |
| JZ007 | *yeats2* | ENSDARG00000078767 | ZF_YEATS2_DON25 | AGAAACTGGCACACACTTACCTGGT | This study |
| JZ007 | *yeats2* | ENSDARG00000078767 | ZF_YEATS2_ACC23 | CCGTGCTGAGGGAGATTGATAATAA | This study |

^a^ Zv10

^b^ Morpholinos target translation initiation codon (ATG) or splice sites (DON – donor, ACC – acceptor, numbers refer to exons)
