# Supplementary material for: Author Correction: Genetic landscape of T cells identifies synthetic lethality for T-ALL
Source: Commun Biol. 2024 Feb 14;7:182. doi: 10.1038/s42003-024-05841-2 (PMC10866859; doi:10.1038/s42003-024-05841-2)
Supplement: Supplementary file 2 — Supplementary_Table_4_CORRECTED [file 42003_2024_5841_MOESM2_ESM.docx]

| **Supplementary Table 4 \| Genotyping primers used in this study.** | | |
| --- | --- | --- |
| **Affected Gene** | **Primer** | **Type** |
| *fli1a* | ATTTCTCAGGCTCTCCAACAG | Forward |
|  | TAGCAAGTCGACTGCTGGTG | Reverse |
| *pole* | GTCTGTGGACATTTGATGCTTG | Forward |
|  | GACTCCAGCTTGGACCCAC | Reverse |
| *tbcb* | ATGAGGAAGAGAGGGCCAAG | Forward |
|  | CCACTGCTGTTAGGTACATC | Reverse |
| *unc45a* | GGGAGCCAAATAGTATTCAAG | Forward |
|  | GCGGTACAGGACTGCACTCT | Reverse |
| *pnrc1* | CATAGACAAGACATCACCTG | Forward |
|  | TGCTTCAGGATGTTTTCTGG | Reverse |
| *ube3d* | TGGATGTGTGGGAGAAGGAC | Forward |
|  | TCAGAGTGGTGTGTGACCTG | Reverse |
| *naa50* | TGCACTGCTGGTTTACGGTG | Mutant |
|  | TTAGGCTCTGTGTTGCATGTG | Mutant |
|  | GTCAGTTCACAGCTAGTTGAC | Wildtype |
|  | GTTGAGTTACGGCTTTGTTGTG | Wildtype |
| *yeats2* | GTCAAAGTAGAACAGGGC | Forward |
|  | ATTCCCTCTGATTGTCCC | Reverse |
| *atad5a* | GACAGGCTCTTCAGTGTTGTC | Forward |
|  | CAGCTTCAAGAGCAAGTCCTG | Reverse |
| *anapc1* | CAGCAGGGCGACTCATTTTG | Forward |
|  | CTGAACTGGGCTGTCGACTG | Reverse |
| *nek7* | CAATTGAACCACCCCAATGT | Forward |
|  | AATGGGCATGTGTCCTTACC | Reverse |
| *spata5* | GTCCGCAGGGTCCAGAGTTAC | Forward |
|  | TGACGGAGCAACAGTTCTGG | Reverse |
| *mat2aa* | CCCAACTAACCAAGCCAAGTT | Forward |
|  | AGTCTCGCTAGTGGCATAAC | Reverse |
| *nol9* | CCAACAGTGTTCTTCAGAACG | Forward |
|  | ATGTGGATTGGACCTGGAAAC | Reverse |
| *eif5* | GCTCTAAATAGGCCTCCGACA | Forward |
|  | CAGTGCATCAAGGGTACACAG | Reverse |
| *pi4kaa* | AAGGTGGAGTGTTGCTTTAAG | Forward |
|  | CGTGACAGTGTCGTTCTTCAG | Reverse |
| *pip5k1ba* | ACTGAAACACAATCAAGCAAGTG | Forward |
|  | CTGTTGCTAAAGACATGTTGTG | Reverse |
